# JP-1302 inhibits RNA polymerase I transcription and induces nucleolar stress in cancer cell lines

**DOI:** 10.1038/s41420-026-03381-8

**Published:** 2026-09-30

**Authors:** Sheetanshu Saproo, Styliani Papadaki, Johana Fernandez-Martinez, Pinky Sultana, Dimitris C. Kanellis, Jiri Bartek, Mikael S. Lindström

**Affiliations:** 1 Science for Life Laboratory, Division of Genome Biology, Department of Medical Biochemistry and Biophysics, Karolinska Institutet, Stockholm, Sweden; 2 Institute of Organic Chemistry and Biochemistry of the Czech Academy of Sciences, Prague, Czech Republic; 3 Department of Physiology, Faculty of Science, Charles University, Prague, Czech Republic; 4 Danish Cancer Institute, Danish Cancer Society, Copenhagen, Denmark

**Keywords:** Drug development, Drug discovery, Chromatin

## Abstract

Cancer cells upregulate ribosomal RNA (rRNA) synthesis to sustain the elevated ribosome production that fuels rapid proliferation, creating a dependency that renders RNA polymerase I (Pol I) transcription a pharmacological vulnerability. JP-1302 is a blood-brain barrier-penetrant 9-anilinoacridine derivative developed as an α_2C_-adrenergic receptor antagonist, but also recently identified as an RNA Pol II inhibitor at micromolar concentrations (~5–10 μM). Here we show that JP-1302 also suppresses Pol I-driven 47S pre-rRNA synthesis at submicromolar-to-low-micromolar concentrations, evidenced by loss of nascent EU-labelled rRNA and reduced activity of an rDNA promoter-driven luciferase reporter. This was accompanied by preferential proteasome-dependent degradation of the Pol I catalytic subunit POLR1A, independent of α_2C_-adrenergic receptor signalling, displacement of POLR1A from the rDNA promoter and progressive disruption of nucleolar morphology. Growth inhibitory IC_50_ values across a panel of normal and cancer cell lines, including glioblastoma, ranged from submicromolar to low micromolar. Nucleolar stress stabilised p53 and induced p21 expression at low-to-intermediate concentrations, without evidence of DNA damage at these doses (no γH2AX increase at concentrations sufficient to abrogate Pol I transcription). Interestingly, p53-null and p53-wild type cells showed comparable S-phase depletion in response to JP-1302. At higher concentrations, JP-1302 additionally caused covalent trapping of TOP2A/TOP2B, accompanied by delayed γH2AX induction, and enrichment of the FACT subunits SSRP1 and SPT16 on chromatin. Together, these findings identify JP-1302 as a pharmacologically distinct inhibitor of ribosome biogenesis with broader, dose-dependent chromatin-disruptive activity.

## Introduction

Cancer cells upregulate ribosomal RNA (rRNA) synthesis to sustain the ribosome production rates that help fuel rapid growth and proliferation [1], making RNA polymerase I (Pol I), dedicatedly transcribing rRNA [2], a rational therapeutic target. Suppressing ribosome biogenesis through Pol I inhibition triggers nucleolar stress and activates p53-dependent and independent checkpoints, leading to cell cycle arrest or death [3]. Many chemotherapeutics engage this pathway. For example, oxaliplatin disrupts ribosome biogenesis rather than inducing DNA damage in colorectal cancer [4–6]. Other drugs including topoisomerase poisons e.g., camptothecin, nucleotide analogues e.g., 5-FU, and DNA intercalators e.g., actinomycin D (ActD) each perturb ribosome biogenesis as part of their pleiotropic mechanisms [7]. Compounds that inhibit the activity of Pol I are often DNA intercalators [8]. Anthracyclines, such as doxorubicin and daunorubicin, which act as topoisomerase inhibitors frequently inhibit Pol I transcription and at concentrations lower than those required to induce widespread DNA breaks [7]. Aclarubicin, a catalytic topoisomerase inhibitor, potently disrupts nucleolar structure and Pol I activity [9, 10].

Several dedicated Pol I inhibitors have been developed, most extensively tested are CX-5461 and BMH-21 [11, 12], yet even these lack absolute specificity and can cause collateral DNA or chromatin damage [13]. BMH-21 is a planar acridine-like molecule that binds GC-rich ribosomal DNA (rDNA) [12, 14, 15], and represses Pol I transcription and tumour cell growth, without activating a canonical DNA damage response, instead it induces nucleolar stress and proteasome-dependent degradation of Pol I subunit POLR1A (RPA194) [15]. Notably, proteasome inhibition rescues POLR1A levels in BMH-21 treated cells but fails to restore 47S pre-rRNA transcription. CX-5461 was characterised as a selective Pol I inhibitor with anticancer activity in clinical trials [11, 16], but was later found to also target TOP2B and stabilise G4 quadruplex structures [17–19]. These activities highlight the need to understand the mechanisms of action and characteristics of Pol I inhibitors. In parallel, high-throughput screens have identified experimental and FDA-approved compounds that inhibit ribosome biogenesis and disrupt the nucleolus [7, 20–22]. Several natural compounds inhibit ribosome biogenesis [23, 24]. For example, the alkaloid nitidine chloride intercalates DNA and inhibits TOP1 and TOP2, generating torsional stress on rDNA that blocks Pol I function and silences rRNA synthesis [23].

Our recent work showed that three different DNA intercalators BMH-21, aclarubicin, and CBL0137 (curaxin), disrupt chromatin structure and transcription by affecting phosphorylation and degrading RNA Pol II, inhibiting Pol I, triggering enrichment of Facilitates Chromatin Transcription (FACT) complex and topoisomerases on chromatin, and evicting histones at high concentrations [9], generating a broader “chromatin damage” state that alters multiple transcriptional processes. Although these intercalators exert broad chromatin effects, Pol I activity is consistently a sensitive readout, effectively inhibited at lower concentrations than other chromatin processes given that Pol I/rDNA is exposed and transcriptionally active across multiple gene copies [8, 10, 13, 25, 26]. Genetic depletion of Pol I via RNAi or degrons independently confirms that Pol I inhibition alone is sufficient to arrest the cell cycle [26–28]. Drug-repurposing efforts have extended this logic to clinically approved agents such as amodiaquine, that exerts unanticipated nucleolar effects, activating p53 and suppressing cancer cell growth [29].

JP-1302 is an experimental compound originally developed as a potent, selective α_2C_-adrenergic receptor antagonist [30]. Chemically, it is a 9-anilinoacridine, an anilino-substituted derivative of the planar tricyclic 9-aminoacridine core, shared by intercalators such as quinacrine [31], aminacrine [32] and ethacridine [33], all of which inhibit Pol I transcription. JP-1302’s asymmetric [4-(4-methylpiperazin-1-yl)-phenyl]amine substituent at the 9-position, a tail group not previously explored in the context of Pol I inhibition, places JP-1302 structurally closer to amsacrine, a clinically tested and used 9-anilinoacridine topoisomerase II poison [34] than to the simpler aminoacridines. This substituent also confers a favourable lipophilicity profile and topological polar surface area (logP 4.88 and TPSA 31.4 Å^2^), properties that likely underlie JP-1302’s documented blood-brain barrier penetrance and in vivo activity at tolerated milligram-per-kilogram doses in rodent models, of particular relevance to malignant brain tumours [35, 36]. Mitchell et al. used proteome-wide profiling to identify JP-1302 as a broad transcriptional perturbagen, linking its activity primarily to FACT inhibition, rapid linker-histone H1 degradation, and suppression of Pol II phosphorylation at micromolar concentrations, thereby reducing Pol II-dependent *CDKN1A* (p21) expression [37], and grouped it closely with the carbazole-derived compound CBL0137 and CDK9 inhibitors [37], but the study did not examine effects on Pol I or the nucleolus. Given JP-1302’s structural resemblance to other Pol I inhibitors and its demonstrated chromatin activity, we investigated whether JP-1302 targets Pol I transcription, and at what concentrations. Our data show that JP-1302 effectively blocks rRNA synthesis and induces nucleolar stress at submicromolar-to-low-micromolar concentrations, triggering p53 and p21 activation through the nucleolar stress pathway.

## Results

### JP-1302 induces proteasome-dependent POLR1A degradation

**Fig. 1:**
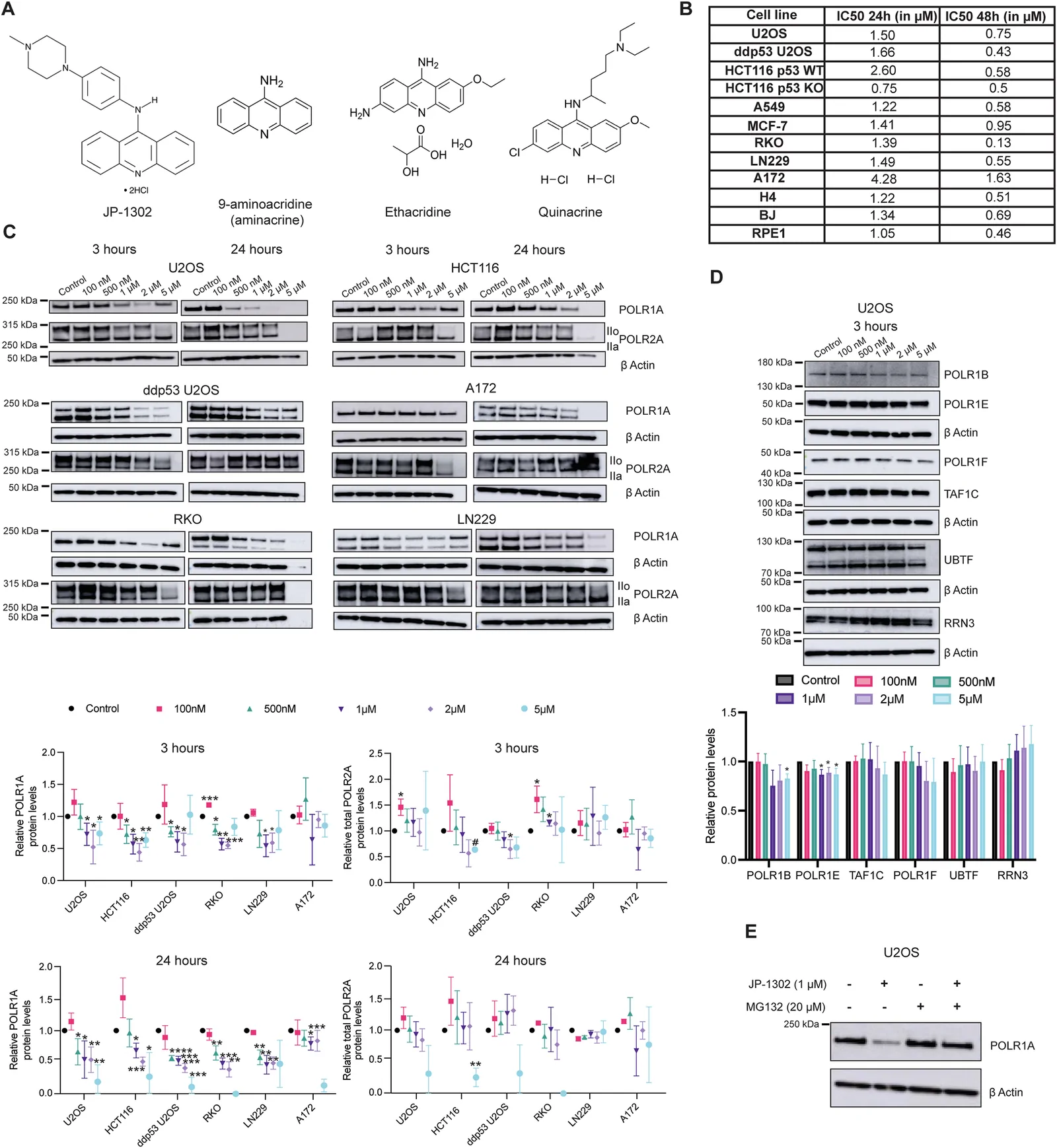
JP-1302 induces POLR1A degradation in cancer cell lines. **A** Structure of JP-1302 in comparison to acridine compounds reported to inhibit Pol I transcription and POLR1A degradation. **B** Table showing IC_50_ values of JP-1302 across various cancer and non-cancer cell lines. **C** Immunoblots showing effect of different concentrations of JP-1302 on POLR1A and POLR2A levels in cancer cell lines. IIa is the unphosphorylated state of POLR2A and IIo shows the phosphorylated POLR2A levels at 3 h and 24 h of treatment. Beta-actin was used as a loading control. Of note, extensive cell death was observed in RKO cells when treated with 5 µM JP-1302 for 24 h, therefore, no detectable protein bands were observed in the RKO cells at this concentration and treatment duration. Lower panel shows the quantification of immunoblots in biological triplicates. Error bars represent mean ± SD. *P < 0.05, **P < 0.01, ***P < 0.001, ****P < 0.0001, ^*#*^*P* < 1 × 10^−^^5^. **D** Immunoblots showing protein levels of Pol I subunits and Pol I-specific pre-initiation complex components in U2OS cells after 3 h of treatment with 100 nM to 5 µM JP-1302. Lower panel shows the quantification of immunoblots in biological triplicates. Error bars represent mean ± SD. *P < 0.05. **E** Immunoblot showing rescue of JP-1302-mediated POLR1A depletion after treatment with proteasome inhibitor, MG132 in U2OS cell line for 3 h.

JP-1302 shares structural similarities with other acridine derivatives known to inhibit Pol I transcription, such as aminacrine [32], ethacridine [33] and quinacrine [31] (Fig. 1A). Among these acridine derivatives, ethacridine [33] and quinacrine [31] are known to target Pol I transcription through degradation of POLR1A. To test whether JP-1302 acts similarly, we first mined the DeepCover MOA proteomic data set [37] and found that JP-1302 markedly downregulates POLR1A relative to most other profiled compounds (https://wren.hms.harvard.edu/DeepCoverMOA/). Resazurin based IC_50_ profiling of JP-1302 across cancer cell lines of varied origin and p53 status (osteosarcoma, glioblastoma, colorectal carcinoma, breast cancer and lung adenocarcinoma) and two non-cancerous lines revealed comparable growth-inhibitory activity across the panel, ranging from 0.75–4.28 µM at 24 h to 0.13-1.63 µM at 48 h (Fig. 1B, Supplementary Fig. S1). IC_50_ values in p53-WT and p53-KO HCT116 cells converged by 48 h (0.58 vs 0.5 µM), suggesting that growth/metabolic inhibition occur largely independent of p53 in the tested cell lines.

Guided by these IC_50_ values, we first assessed POLR1A protein levels across a concentration range in various cancer cell lines and at 3 h and 24 h time points. JP-1302 treatment resulted in a concentration-dependent reduction of POLR1A protein, with depletion generally evident from 500 nM onward and most pronounced at 1–5 µM at 24 h (Fig. 1C and Supplementary Fig. S2). The magnitude and kinetics of this response varied between cell lines. For example, A172 glioma cells showed comparatively weak POLR1A depletion after 3 h, but exhibited clearer depletion at higher concentrations and longer exposure to JP-1302 (Fig. 1C). In contrast, POLR1A levels in BJ fibroblasts were largely preserved under the same conditions (Supplementary Fig. S2).

Since JP-1302 was previously reported to inhibit Pol II phosphorylation at 3-10 µM [37], we also in comparison examined POLR2A using an antibody (clone D8L4Y) that detects both unphosphorylated and hyperphosphorylated states of the protein [9]. Phosphorylated POLR2A (IIo) fluctuated with treatment, while combined total levels of POLR2A were largely unaltered except at 5 µM, where both IIa and IIo were depleted in U2OS and HCT116 cells (Fig. 1C and Supplementary Fig. S2), indicating that POLR2A degradation, unlike POLR1A, requires higher concentrations. Among other Pol I preinitiation complex components tested, only POLR1E showed a marginal but significant reduction at low concentrations, and far less than POLR1A. (Fig. 1D). Since JP-1302 affected protein levels of RNA polymerases, we used quantitative real-time PCR (qPCR) assay to rule out transcriptional repression of *POLR1A* itself. *POLR1A* mRNA was unaltered (Supplementary Fig. S3). Co-treatment of U2OS cells with JP-1302 and the proteasome inhibitor MG-132 for 3 h rescued POLR1A protein levels (Fig. 1E), indicating proteasome-mediated degradation. Finally, knockdown of the α_2C_-adrenergic receptor (*ADRA2C*) did not affect POLR1A protein levels (Supplementary Fig. S4), suggesting that POLR1A degradation occurs independently of JP-1302´s adrenergic receptor antagonism.

### JP-1302 reduces Pol I-mediated transcription in cancer cell lines

**Fig. 2:**
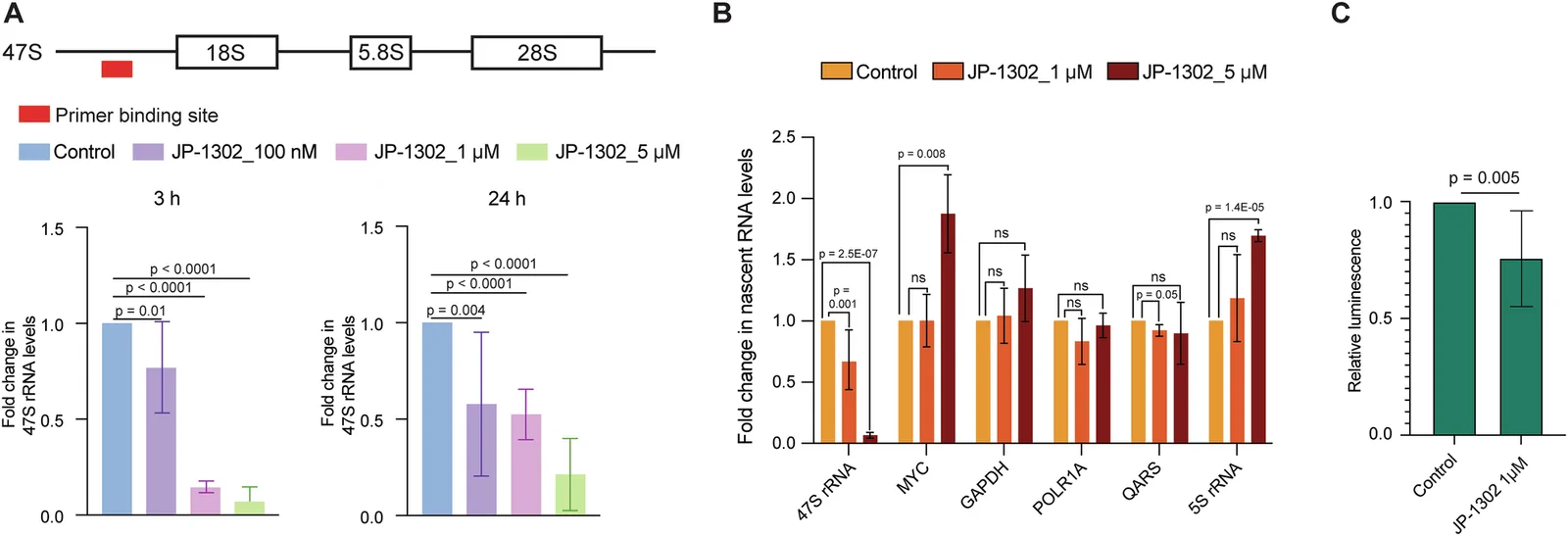
Suppression of Pol I transcription in JP-1302 treated cells. **A** Bar plots showing reduced 47S rRNA levels after treatment of U2OS cells with varying concentrations of JP-1302 for 3 h and 24 h. Error bars represent mean ± SD of biological triplicates. **B** Bar plot showing nascent RNA synthesis levels of Pol I transcript (47S rRNA), Pol II transcripts (MYC, GAPDH, POLR1A, and QARS) as well as Pol III transcript 5S rRNA after 1 µM or 5 µM JP-1302 treatment in U2OS cells for 3 h. Error bars represent mean ± SD of biological triplicates. **C** Bar plot showing reduced relative luminescence in an rDNA core promoter-driven luciferase reporter construct upon treatment with 1 µM JP-1302 for 3 h in U2OS cells. Error bars represent mean ± SD of biological triplicates.

Given POLR1A degradation, we examined the primary Pol I transcript, 47S pre-rRNA. qPCR in U2OS cells treated with different concentrations of JP-1302 for 3 h and 24 h showed significant 47S downregulation from 100 nM (Fig. 2A). To confirm this reflects active transcriptional inhibition rather than altered RNA turnover, we used a nascent RNA capture assay based on ethynyl uridine (EU)-labelled RNA pulldown. Nascent 47S rRNA was significantly reduced, with no significant changes in nascent Pol II transcripts *GAPDH, POLR1A*, although a modest decrease was noted in *QARS*. Interestingly, MYC nascent RNA was significantly increased (1.9-fold) and the nascent Pol III transcript 5S rRNA was also increased (1.7-fold) at 5 µM JP-1302 (Fig. 2B). The increase in MYC and 5S rRNA may reflect secondary or stress induced compensatory transcription. To test rDNA promoter activity directly, we used a plasmid-based rDNA core-promoter luciferase reporter assay. Luciferase activity was significantly, though modestly reduced after 3 h of JP-1302 treatment (Fig. 2C), a smaller effect than seen at the endogenous locus, possibly caused by the reporter´s lack of native chromatin context. Together, these results demonstrate that JP-1302 suppresses Pol I transcription.

### JP-1302 disrupts nucleolar morphology in cancer cells

**Fig. 3:**
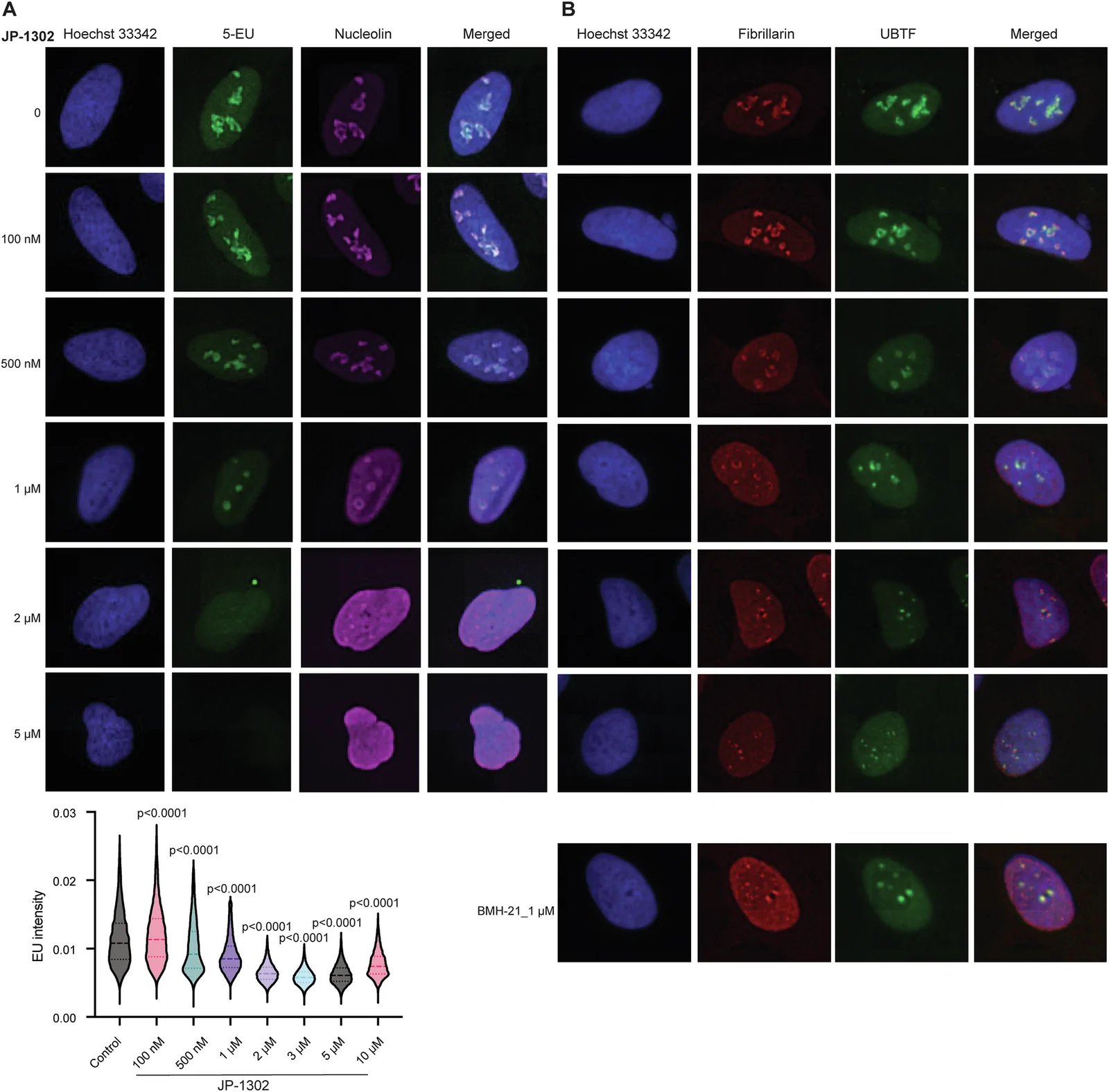
JP-1302 induces changes in nucleolar structure. **A** Images showing incorporation of nascent RNA in U2OS cells after 3 h JP-1302 treatment using 5-ethynyl uridine (5-EU). Nucleolin was used as a nucleolar marker. Hoechst 33342 was used to stain the nuclei. Lower panel shows reduction in EU intensity with increasing concentration of JP-1302. The experiment was performed in biological triplicates. **B** Fluorescence images showing nucleolar disassembly as depicted by nucleolar markers for DFC (Fibrillarin) and UBTF as a marker of active rDNA transcription units in response to varied concentrations of JP-1302, treated for 3 h. 1 µM BMH-21 was used as a positive control. Hoechst 33342 was used to stain the nuclei. The experiment was performed in biological triplicates.

We combined EU incorporation with nucleolin (NCL) staining to visualise nascent RNA synthesis and nucleolar morphology, respectively. In U2OS cells, 3 h of JP-1302 treatment revealed a significant reduction in nascent RNA synthesis starting from 500 nM of JP-1302 (Fig. 3A). Moreover, a prominent nucleolar ring was formed by NCL in all the cells at 1 µM JP-1302 with progressive NCL redistribution into the nucleoplasm at higher concentrations (Fig. 3A). This nucleolar ring formation by NCL is an indicator of nucleolar stress caused by impaired Pol I- mediated transcription [38]. To further describe the nucleolar disruption, we utilised specific markers for different nucleolar sub-compartments; fibrillarin as a marker for the dense fibrillar component and UBTF as a marker for fibrillar centre. We observed nucleolar changes in fibrillarin distribution from 100 nM JP-1302 treatment, visible nucleolar caps at 2 µM and a more complete nucleolar separation at 5 µM (Fig. 3B). Taken together, these data show a dose-dependent progression from nucleolar ring and cap formation to full nucleolar architectural collapse.

### JP-1302 activates the p53 response to nucleolar stress

**Fig. 4:**
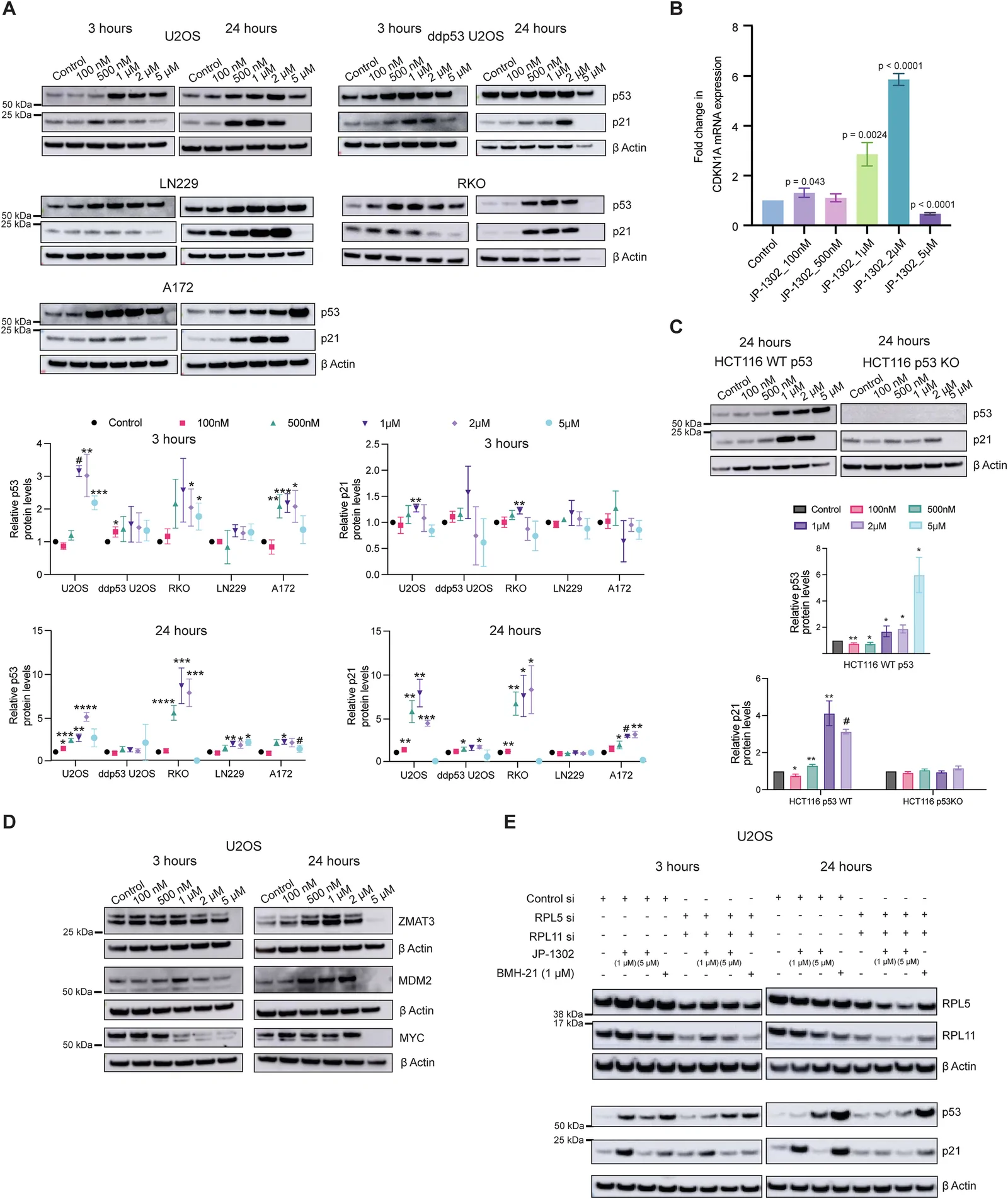
Nucleolar stress and activation of p53 following exposure to JP-1302. **A** Immunoblots showing stabilisation of p53 with increasing concentrations of JP-1302 in cancer cell lines at both 3 h and 24 h of treatment. The levels of p21 increased in accordance with p53 levels; however, at high concentration of JP-1302 (5 µM), there was a reduction in p21 protein levels at both 3 h and 24 h treatments. Lower panel shows quantification of the blots in biological triplicates, represented as mean ± SD. Of note, ddp53 U2OS cells express wild type p53 and an engineered dominant-negative p53. The quantifications are therefore a combination of both p53 types. *P < 0.05, **P < 0.01, ***P < 0.001, ****P < 0.0001, ^*#*^*P* < 1 × 10^−^^5^. **B** Bar plots showing increased levels of *CDKN1A* (p21) mRNA in U2OS cells after JP-1302 treatment for 24 h, up to 2 µM concentrations and reduction at 5 µM concentration. Error bars represent mean ± SD of biological triplicates. **C** Immunoblot showing increased p21 protein levels with increased concentrations of JP-1302 up to 2 µM in HCT116 WT p53 cells and reduction in p21 protein levels at 5 µM in HCT116 p53 WT and KO cells. Lower panel shows quantification of the blots, represented as mean ± SD of biological triplicates. **D** Immunoblots showing effect of JP-1302 treatment for 3 h and 24 h on protein levels of p53 targets, ZMAT3 and MDM2; and MYC as a Pol II-dependent, non-p53-induced control in U2OS cells. **E** Immunoblots showing effect of RPL5 and RPL11 knockdown on JP-1302-mediated modulation of p53 and p21 proteins at 1 µM and 5 µM concentrations of JP-1302. BMH-21 (1 µM) was used as a positive control. All the experiments were performed in biological triplicates.

Since nucleolar stress activates p53-signalling in cells [39], we assessed p53 stabilisation and *CDKN1A* (p21) induction. Across the cell-line panel, JP-1302 induced p53 accumulation at submicromolar concentrations, with the response generally evident from ~500 nM after 3 h. Of note, ddp53 U2OS is an engineered cell line expressing both WT p53 and dominant negative p53, where dominant negative p53 further blocks the function of WT p53. Consistent with p53 stabilisation and activation, p21 protein increased substantially up to 2 µM JP-1302 but fell significantly at 5 µM JP-1302 (Fig. 4A, Supplementary Fig. S5). Further analysis of *CDKN1A* mRNA revealed a significant downregulation of *CDKN1A* at the highest concentration used (Fig. 4B), indicating a p53-independent mechanism of p21 depletion. To directly assess p53 dependence, we compared p53-WT or p53-KO HCT116 cells. As expected, JP-1302 was unable to cause p21 upregulation in p53-KO HCT116 cells. However, treatment with 5 µM JP-1302 resulted in depleted p21 levels in both p53-WT and p53-KO HCT116 cells (Fig. 4C). This concentration-dependent uncoupling of p53 stabilisation from p21 expression is consistent with the proteome-level relationship reported by Mitchell et al., in which JP-1302 increased p53 while failing to induce p21 at transcription-inhibitory concentrations [37]. We tested two other p53 targets, ZMAT3 and MDM2 as well as the transcription factor MYC. Notably, the p53 targets and MYC followed similar response at highest concentration of JP-1302 **(**Fig. 4D**)**. The decline in steady-state MYC protein contrasts with the early-time point increase in nascent MYC transcription, indicating that transcriptional output and protein abundance are not concordant for MYC.

Pol I inhibition typically activates p53 via the impaired ribosome biogenesis checkpoint (IRBC), in which free RPL5/RPL11/5S rRNA binds and inhibits MDM2´s E3 ligase activity [40]. To understand whether JP-1302-mediated p53 activation followed the same pathway, we simultaneously knocked down RPL5 and RPL11 using siRNAs. Co-depletion of RPL5 and RPL11 reduced JP-1302-mediated stabilisation of p53 and activation of p21 (Fig. 4E), indicating that JP-1302 activates p53 at least partially through the IRBC. Collectively, these data indicate that JP-1302 activates a nucleolar stress pathway at low-to-intermediate concentrations.

### JP-1302 induces a concentration-dependent cytostatic-to-cytotoxic response

**Fig. 5:**
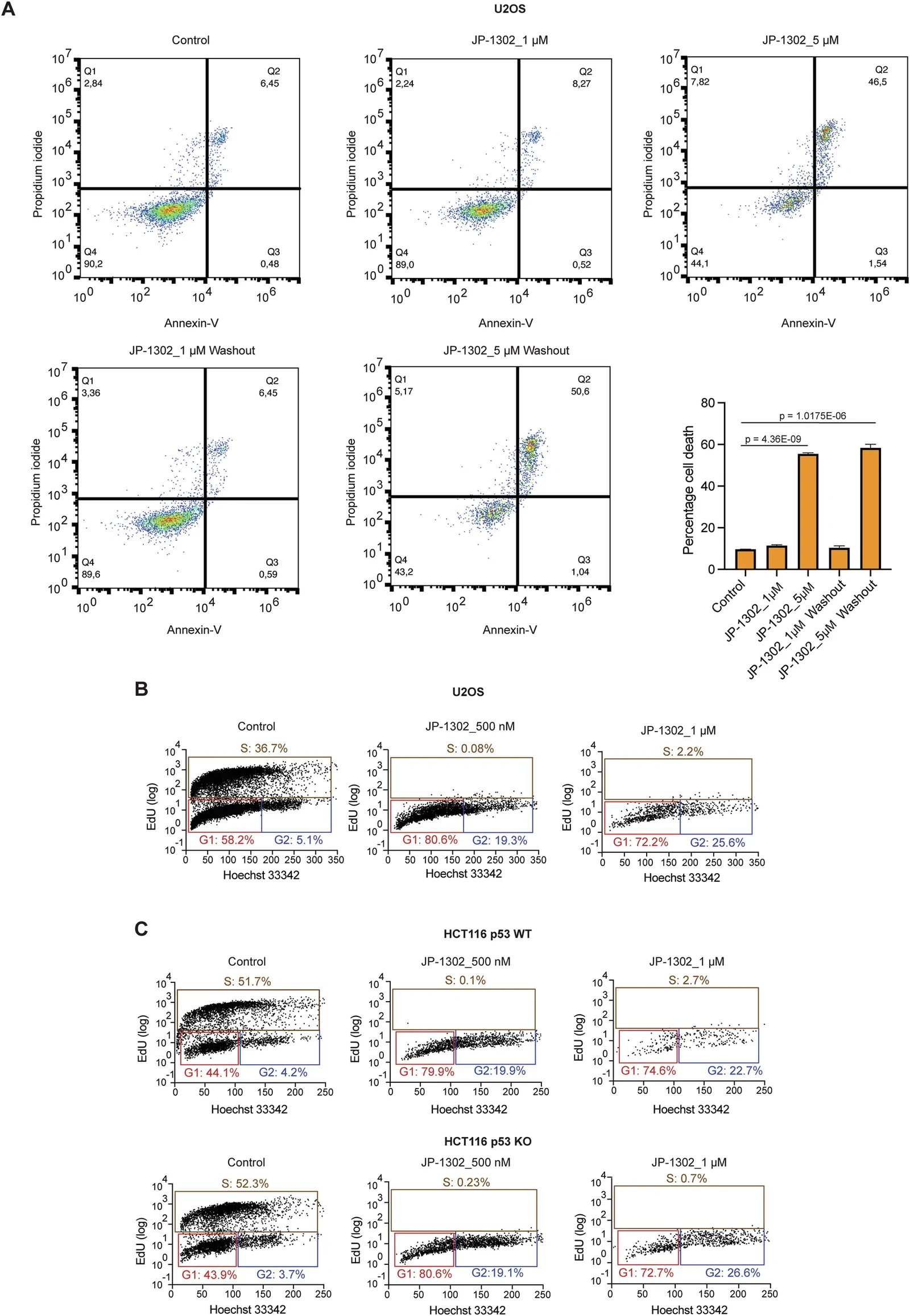
Concentration-dependent cell-cycle inhibition and induction of cell death in cancer cells treated with JP-1302. **A** FlowJo analysis showing Annexin-V/propidium iodide-based cell-death assay after treatment of U2OS cells with 1 µM or 5 µM of JP-1302 for 24 h or treatment for 8 h followed by a washout of JP-1302 and recovery in compound-free complete medium for 16 h compared to vehicle control (DMSO). Bar graph on the right shows the percentage of cell death calculated as a sum of Q1, Q2 and Q3 quadrants. The data is represented as mean ± SD of biological triplicates. **B** EdU incorporation assay showing cell cycle arrest of U2OS cells treated with 500 nM and 1 µM of JP-1302 for 24 h. **C** EdU incorporation assay showing cell cycle arrest in HCT116 p53 WT and p53 KO cells treated with 500 nM and 1 µM of JP-1302 for 24 h. All the experiments were performed in biological triplicates.

Given that nucleolar stress and IRBC activation can trigger cell cycle arrest or apoptosis [41], we assessed cell fate in U2OS cells treated with 1 µM or 5 µM of JP-1302 for 24 h. Furthermore, to test the reversibility of phenotype after compound removal, we washed out the compound after 8 h of treatment and incubated the cells for recovery in complete medium. No significant cell death was observed at 1 µM, with effects compatible with full recovery after washout, whereas 5 µM caused significant, washout-irreversible cell death (Fig. 5A). Since resazurin assays had shown reduced activity even at low concentrations of JP-1302 (Fig. 1B), we asked whether low-dose JP-1302 affects the cell cycle. Ethynyl deoxyuridine (EdU) incorporation after 24 h of JP-1302 treatment showed S-phase depletion and accumulation of cells in the G1/G2 populations, independently of p53 status (Figs. 5B, 5C). Together, these data indicate that low concentration of JP-1302 induces cell cycle arrest, while at higher concentration and additional mechanisms, it causes rapid and extensive cell death.

### JP-1302 intercalates DNA and traps topoisomerases on chromatin

**Fig. 6:**
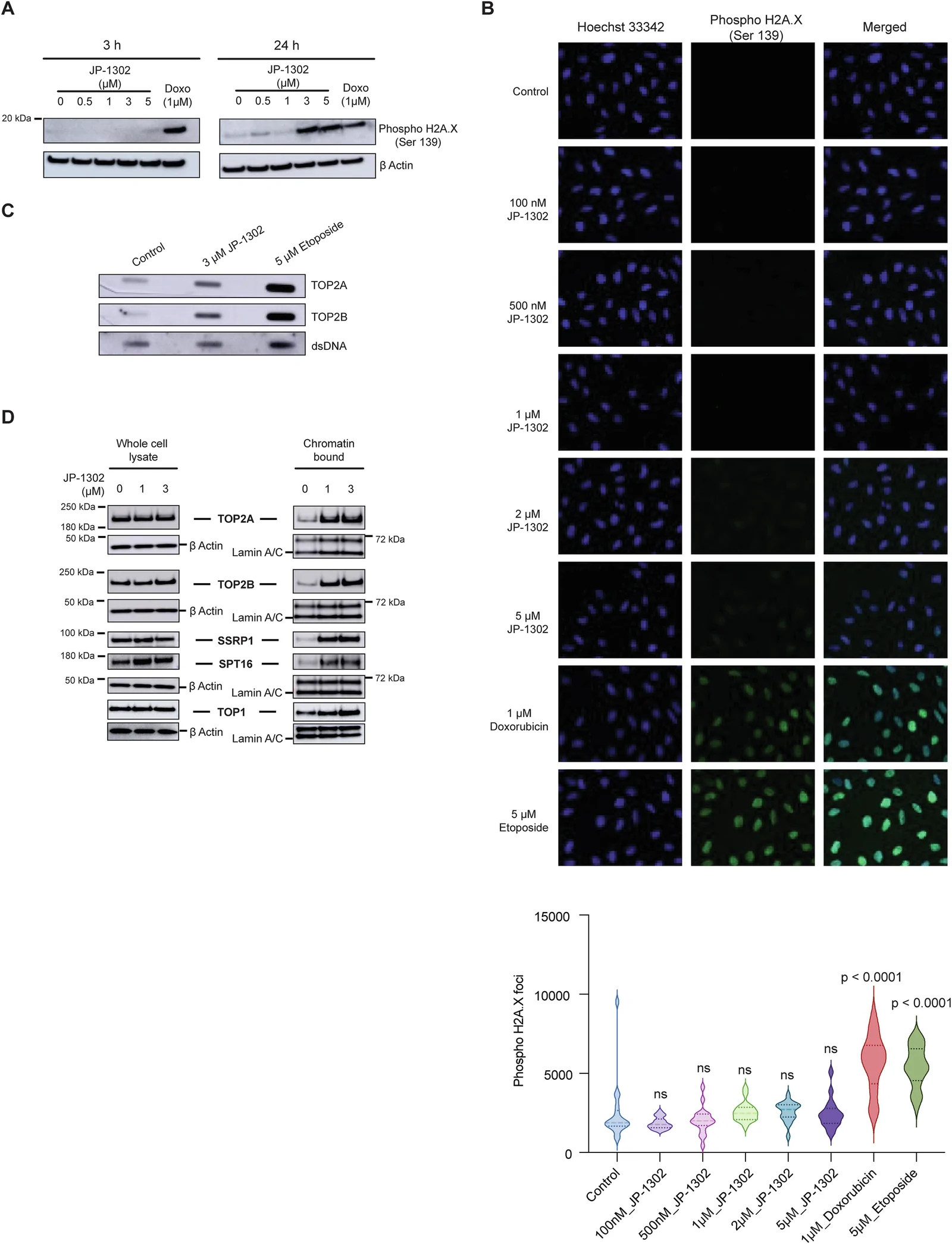
FACT chromatin enrichment and TOP2A/2B covalent trapping in JP-1302 treated cells. **A** Immunoblot showing no phosphorylation of H2A.X at concentrations of JP-1302 up to 1 µM for 3 h and 24 h in U2OS cells. However, phosphorylation of H2A.X was observed at 3-5 µM JP-1302 after 24 h of treatment in U2OS cells. Doxorubicin was used as a positive control. The experiment was performed in biological triplicates. **B** Immunofluorescence image analysis showing no significant γH2AX foci in U2OS cells treated with JP-1302 for 3 h compared to DMSO control. Doxorubicin and Etoposide were used as positive controls. Lower panel shows quantification of γH2AX foci from biological triplicates. **C** RADAR assay showing covalent trapping of TOP2A and TOP2B on DNA after treatment with 3 µM JP-1302 for 3 h in U2OS cells. Etoposide (5 µM) was used as a positive control. The experiment was performed in biological triplicates. **D** Subcellular fractionation assay showing chromatin enrichment of TOP2A, TOP2B, TOP1 and FACT complex subunits SSRP1 and SPT16 in chromatin fractions after treatment of U2OS cells with 1 µM or 3 µM JP-1302 for 3 h. Beta-actin was used as a loading control for whole cell lysates and Lamin A/C was used as a loading control for chromatin fractions.

JP-1302 shares functional features with the chromatin destabilizer CBL0137 [9, 37]. We therefore investigated JP-1302´s effects on DNA damage and chromatin stability. Interestingly, JP-1302 did not induce Ser139 phosphorylation of H2A.X (γH2AX) at any concentration at 3 h (Fig. 6A, B); at 24 h γH2AX was detected only at the highest concentrations (Fig. 6A), indicating that the classical DNA damage response is not engaged at concentrations sufficient to impair Pol I transcription. To provide experimental support for JP-1302 DNA intercalation, we first performed molecular docking using the known intercalator ellipticine as a reference. JP-1302 docked into the vacated ellipticine CpG intercalation site of DNA hexamer d(CGATCG)₂ with a modelled binding affinity of −7.65 kcal/mol, its acridine chromophore sandwiched between flanking base pairs and closely overlapping the ellipticine position (Supplementary Fig. S6). Second, to test this prediction experimentally, we performed a fluorescence intercalator displacement (FID) assay across oligonucleotides spanning 0–87.5% GC content. The reference intercalator ActD displaced EtBr–DNA fluorescence in a GC-content-dependent manner, as expected of a GC-preferring intercalator. JP-1302, by contrast, displaced EtBr–DNA fluorescence across the full GC-content range at all concentrations examined (10, 20 and 50 µM) with no clear GC dependence (Supplementary Fig. S7). Thus, molecular docking and FID assay support that JP-1302 intercalates DNA, and indicate that under these assay conditions, unlike ActD and BMH-21’s documented preference for GC-rich rDNA sequences, we did not observe an obvious dependence of EtBr displacement on the GC-content range tested.

To test whether JP-1302 also traps topoisomerases on genomic DNA, we used the RADAR (Rapid Approach to DNA Adduct Recovery) assay, which isolates covalently DNA-trapped proteins, while excluding non-covalent binders. Etoposide, a well-characterised topoisomerase II poison that stabilises covalent TOP2-cleavable complexes, and also inhibits Pol I, was used as a positive control [42]. The data revealed that JP-1302 (3 µM) increased the occupancy of TOP2A and TOP2B in U2OS cells (Fig. 6C). Chromatin fractionation similarly showed enrichment of TOP2A, TOP2B, TOP1 and the FACT complex components SPT16 and SSRP1 in the chromatin-bound fraction at 1 and 3 µM of JP-1302 (Fig. 6D). We performed chromatin immunoprecipitation (ChIP) assays to understand whether the enriched or trapped factors are localised to rDNA following JP-1302 treatment. ChIP-qPCR at rDNA promoter (−250 to +102 bp of the transcription start site) showed reduced occupancy of POLR1A, TOP2A, and SSRP1 following treatment (Supplementary Fig. S8), consistent with possible redistribution of TOP2A and SSRP1 away from rDNA into non-rDNA chromatin, and similar to what has been observed upon CBL0137 treatment [43]. In summary, chromatin enrichment of FACT and topoisomerase trapping were clearly detectable at 1–3 µM.

## Discussion

Pol I drives highly active rRNA synthesis across numerous topologically accessible ribosomal DNA (rDNA) repeats [44], making Pol I transcription exposed and vulnerable to DNA-intercalating compounds not originally developed to target it [13]. In addition, biochemical and single-molecule studies report differences in Pol I’s functional properties like elongation dynamics, and response to DNA damage and topology, that have the potential to make it directly or indirectly more sensitive to such inhibitors than Pol II [45, 46]. The proteome-wide study by Mitchell et al. identified JP-1302 as a broad transcriptional perturbagen based on its similarity to CDK9 inhibitors and CBL0137, and demonstrated suppression of Pol II phosphorylation [37]. Our findings extend these observations to Pol I. At the molecular level, JP-1302 displaces POLR1A from the rDNA promoter, triggers proteasome-dependent and selective degradation of POLR1A without equivalent degradation of other core Pol I subunits or initiation factors, and reduces both steady-state and nascent 47S rRNA levels. Docking and FID data support DNA intercalation by JP-1302, but with no clear GC-content dependence, unlike GC-preferring intercalators ActD and BMH-21. The effect on Pol I is detectable well below the ~3–10 μM range at which JP-1302 has previously been reported to inhibit Pol II phosphorylation, and consistent with our finding that up to 2 μM its most visible impact is on nucleolar structure. This does not exclude partial Pol II effects at low concentrations, which could also feedback negatively on ribosome biogenesis through various mechanisms.

JP-1302 did not induce γH2AX at 3 h at any concentration tested in U2OS cells, including the dose at which RADAR detected covalent trapping of TOP2A/B. By 24 h, however, γH2AX became detectable at 3 and 5 µM, the same concentration range in which covalent TOP2 complexes were captured at the earlier timepoint. This would be consistent with TOP2 engagement by JP-1302, and in line with its structural proximity to amsacrine. By contrast, the lower, Pol I-inhibitory and nucleolar-stress-inducing concentrations of JP-1302 remained free of γH2AX at both 3 and 24 h. Although it should be acknowledged that cell lines are likely to differ in their response. More broadly, an absence of acute DNA damage response despite nucleolar disruption is shared with several structurally diverse Pol I suppressors, including BMH-21, aclarubicin, amodiaquine and acridine derivatives [9, 12, 29, 33, 47–50]. However, the reason for lack of γH2AX among the compounds may differ, for example, BMH-21 antagonises TOP2-DNA complex formation induced by bona fide TOP2 poisons [9].

Structurally, JP-1302 sits at the intersection of two acridine lineages. It was originally developed as a selective α_2C_-adrenoceptor antagonist (K_i_ ≈28 nM) built on a 9-aminoacridine scaffold [30], and α_2C_ blockade may modestly sensitise cancer cells by attenuating pro-survival adrenergic signalling [51], though *ADRA2C* knockdown did not attenuate JP-1302-induced POLR1A degradation, indicating that this effect is independent of receptor engagement. In agreement, the JP-1302 proteome phenotype occurred in a cell line (HCT116) lacking detectable *ADRA2C* expression [37]. At the same time, as an anilinoacridine, JP-1302 is structurally closer to amsacrine than to the simpler 9-aminoacridines quinacrine and aminacrine [34]. Amsacrine is known as an anilinoacridine TOP2 poison developed as an antineoplastic agent and tested primarily in the treatment of acute leukaemias, particularly relapsed/refractory disease [34]. Interestingly, JP-1302 had only a weak proteome correlation with amsacrine despite its structural similarity, suggesting that JP1302´s whole-cell effect is not directly amsacrine-like and may occupy an unusual pharmacological space [37]. JP-1302 thus combines rRNA synthesis inhibition with α_2C_ antagonism in one scaffold.

The cellular response to JP-1302 shows a concentration dependence that is important for interpreting its mechanism. Between 100 nM to 2 µM, rRNA synthesis is suppressed, nucleolar morphology is disrupted, IRBC is engaged, p53 is stabilised, and *CDKN1A* transcription is activated. Interestingly for JP-1302, the resulting cell-cycle effect is p53-independent since HCT116 p53-KO cells and ddp53 U2OS cells show comparable S-phase depletion as their wild type counterparts, indicating the compound blocks cell cycle progression independently of p53 signalling. At micromolar concentration RADAR revealed covalent trapping of TOP2A/B, followed by γH2AX clearly detected at 24 h, and at 5 µM, total POLR2A levels also decline. This combined load, Pol II depletion, FACT/TOP2 chromatin enrichment and trapping, and DNA damage, likely drives cell death through a p53-independent, largely irreversible pathway. Indeed, Pol II degradation itself, regardless of transcription, has recently been identified as a pro-apoptotic signal [52], and our washout experiments show that JP1302´s effect at 5 µM is irreversible.

Some limitations should be noted. First, current data do not demonstrate a robust in vitro therapeutic window, with IC50 values in BJ fibroblasts and RPE1 cells falling within the same low-micromolar range as the most sensitive cancer lines. Whether the pharmacokinetic properties of JP-1302 create a meaningful exposure difference in vivo remains to be established. Second, we did not perform an in vitro reconstituted Pol I transcription assay, which would provide definitive evidence of direct Pol I inhibition. Third, although POLR1A is clearly the most sensitive Pol I component to JP-1302 treatment, the upstream mechanism to selective proteasomal targeting of POLR1A remains unresolved, although the FBXL14 E3 ligase is a strong candidate [53]. Fourth, the contributions of TOP2 and FACT chromatin engagement to Pol I inhibition specifically remains incompletely resolved, since our chromatin-trapping data apply to the higher-dose regime (1-3 µM), whereas suppression of 47S synthesis is already evident from 100 nM. However, it is also known that FACT co-purifies with Pol I complexes, SSRP1 occupies the rRNA gene repeats, and FACT knockdown impairs Pol I elongation [54]. Moreover, inactivating TOP2 selectively disrupted rDNA chromatin structure during transcription [55].

A notable feature of JP-1302 is its CNS pharmacokinetic profile, including blood-brain barrier penetrance [56] and documented in vivo activity at tolerated milligram-per-kilogram doses in rodent models [35, 36]. The three glioblastoma cell lines tested here (LN229, A172, H4) all showed low micromolar IC50 values, and given that a_2C_-adrenergic receptors are expressed in specific brain structures and glioblastoma microenvironments including pericytes, JP-1302´s dual pharmacology makes it an interesting candidate and perturbagen for exploration in malignant brain tumours [57]. Further in vivo validation particularly in orthotopic glioma models will be important, as blood-brain barrier penetrance is not evidence of therapeutic exposure in brain tumours. Our findings suggest several other directions. Chemical optimisation of JP-1302 may produce analogues with improved potency and selectivity. The acridine scaffold and tail group are clearly amenable to modification, and previous work on BMH-21 and amodiaquine analogues demonstrated that relatively small changes can alter DNA-binding and Pol I inhibitory activity [29, 58]. Combination strategies are also warranted. Pol I inhibition is known to activate pro-survival autophagy in solid tumours, and pairing JP-1302 with autophagy inhibitors may boost efficacy [59, 60]. In summary, our findings position JP-1302 as a distinct and interesting ribosome biogenesis inhibitor.

## Materials and methods

### Cell culture

The details on cell lines and drugs used are provided in Supplementary Tables S1 and S2, respectively. For siRNA transfections, 4×10^5^ U2OS cells were transfected with control siRNA/1:1 mix of 20 nM RPL5 and RPL11 siRNA SMARTpool (Dharmacon, GE Healthcare, Lafayette, Colorado, USA) for 24 h or 20 nM ADRA2C siRNA SMARTpool (Dharmacon) for 72 h. The transfected cells were treated either with DMSO or JP-1302 or BMH-21 for 3 or 24 h.

### Resazurin assay/IC_50_ measurements

Two thousand cells per well were seeded in 96-well plates and 18-22 h post-seeding, treated with 100 nM to 20 µM JP-1302 (or vehicle). After 24 or 48 h treatment, resazurin solution in DPBS (Sigma-Aldrich) was added to each well at a final concentration of 15 µg/mL and incubated for 4 h in CO_2_ incubator. The emitted fluorescence was measured using Tecan Infinite M1000 Pro microplate reader. Dose-response curves and IC_50_ values were generated using GraphPad Prism 10.0.

### Immunoblotting

Immunoblotting was performed as described previously [9]. Details on primary and secondary antibodies are provided in Supplementary Table S3. The images of uncropped blots are provided in supplemental information labelled “Original data”. Chemiluminescent signals were acquired using an Amersham Imager 680 and quantified using Image Lab 6.1 software (Bio-Rad Laboratories).

### RNA isolation and quantitative real-time PCR

Total RNA was isolated using PureLink^TM^ RNA Mini kit (ThermoFisher Scientific) following manufacturer’s guidelines and quantified using NanoDrop Lite microvolume spectrophotometer (ThermoFisher Scientific). 10 ng of total RNA was used for qPCR using Power SYBR Green RNA-to-C_T_^TM^ 1-step kit (ThermoFisher Scientific) in Quant Studio 5 thermal cycler (ThermoFisher Scientific). Data was analysed using 2^−ΔΔCt^ method. The experiments were performed in biological triplicates, with technical triplicates per experiment. Primer sequences are provided in Supplementary Table S4.

### Nascent RNA capture assay

The total nascent RNA was pulled down using Click-iT^®^ Nascent RNA Capture Kit (ThermoFisher Scientific) as per the manufacturer’s instructions. Briefly, 1 × 10^6^ U2OS were treated with 1 µM or 5 µM JP-1302 or DMSO for 3 h and then were labelled with 0.5 mM EU for 1 h followed by total RNA isolation. The nascent RNA pool was then pulled-down using biotinylated azide and 50 ng of nascent RNA was subjected to cDNA synthesis using SuperScript^®^ VILO^™^ cDNA synthesis kit (ThermoFisher Scientific). The nascent RNA levels for 47S rRNA, c-MYC, GAPDH, POLR1A, QARS, and 5S rRNA were quantified using qPCR.

### Ethynyl uridine incorporation and immunofluorescence

The details on the method and the uncropped original images are provided in the supplemental information.

### Luciferase assay

0.5 × 10^5^ U2OS cells per well in a 12-well plate were transfected with 10 ng Renilla and 500 ng rDNA promoter plasmids using Lipofectamine 3000 (ThermoFisher Scientific). The plasmids were received as a gift from Jacob laboratory, Ohio University [61]. After 24 h of transfection, the cells were treated with 1 µM JP-1302 or DMSO for 3 h followed by luciferase measurement of firefly luciferase activity and Renilla luciferase activity using Dual-Luciferase^®^ Reporter Assay System (Promega) on Tecan Infinite M1000 Pro microplate reader.

### Ethynyl deoxyuridine incorporation assay

Five thousand cells per well in a 96-well plate were treated either with DMSO or JP-1302 for 24 h followed by incubation with 10 µM EdU for 1 h. Cells were labelled according to manufacturer’s instruction (Click-iT EdU Imaging kits, Invitrogen). Images were captured using InCell and analysed using Cell Profiler. Integrated Hoechst 33342 intensity (linear) and a log of integrated EdU intensity were plotted in GraphPad Prism to obtain cell populations in different phases of cell cycle.

### Flow cytometry

1 × 10^5^ cells per well in a 6-well plate were treated with DMSO or varying concentrations of JP-1302. The cells were either incubated with JP-1302 for 24 h or 8 h followed by the washout of the compound and recovery in complete medium without compound for 16 h. Total cell death was analysed using Annexin V-FITC apoptosis staining kit (Abcam, Cambridge, UK), according to manufacturer’s instructions. The cells were acquired using Guava easycyte flow cytometer and data was analysed using FlowJo software (BD Biosciences, Franklin Lakes, New Jersey).

### Subcellular fractionation assay

1 × 10^6^ U2OS cells were treated either with DMSO or JP-1302. Following 3 h of treatment, cells were fractionated using Subcellular Protein Fractionation kit (#78840 ThermoFisher Scientific). Ten percent of the treated cells, before processing for chromatin fractionation, were used as whole cell lysates. The fractions obtained were analysed using western blotting.

### RADAR assay

DNA-topoisomerase covalent complexes were analysed using the RADAR assay. Briefly, 1 × 10^6^ cells were treated either with DMSO or 3 µM JP-1302 or 5 µM Etoposide (positive control) for 3 h. The assay was performed as described previously [9].

### Statistics

Data were presented as mean ± standard deviation (SD). The statistical significance between two groups was calculated using unpaired two-tailed Student’s t-test and between multiple groups was calculated using One-way ANOVA in GraphPad Prism. *P*-values < 0.05 were considered statistically significant. Number of biological replicates (*n*) is indicated in figure legend.

## Supplementary information

Original Data

Supplemental file

## Data Availability

All data generated or analysed during this study are included in this published article and its supplementary information files, including uncropped original western blots (“original data”). Further information is available from the corresponding author upon request.
