# Supplementary material for: JP-1302 inhibits RNA polymerase I transcription and induces nucleolar stress in cancer cell lines
Source: Cell Death Discov. 2026 Sep 30;12:389. doi: 10.1038/s41420-026-03381-8 (PMC13627690; doi:10.1038/s41420-026-03381-8)
Supplement: Supplementary file 1 [file 41420_2026_3381_MOESM1_ESM.pdf]

Uncropped immunoblot images:

Figure 1C

U2OS 3h

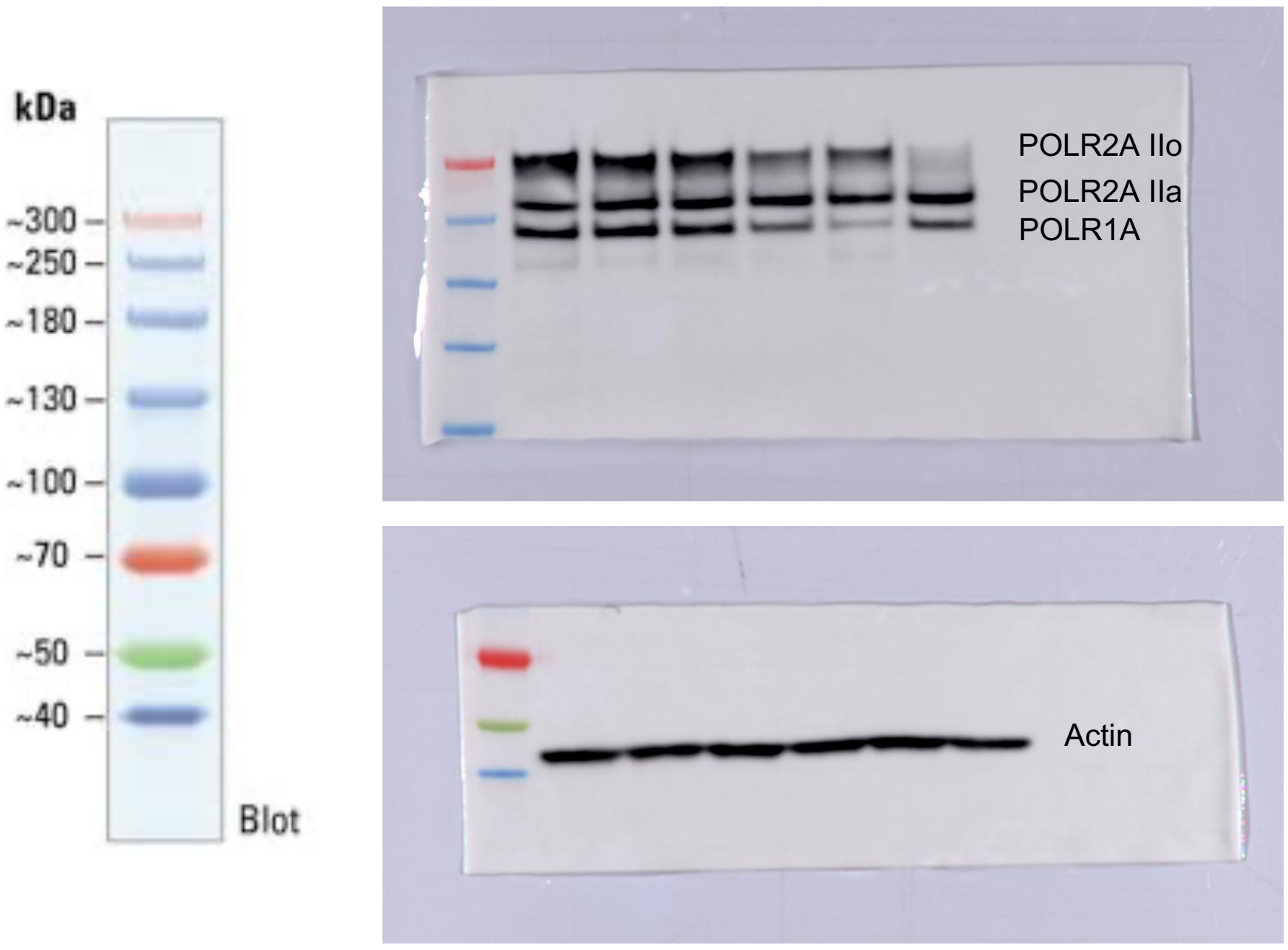

U2OS 24h

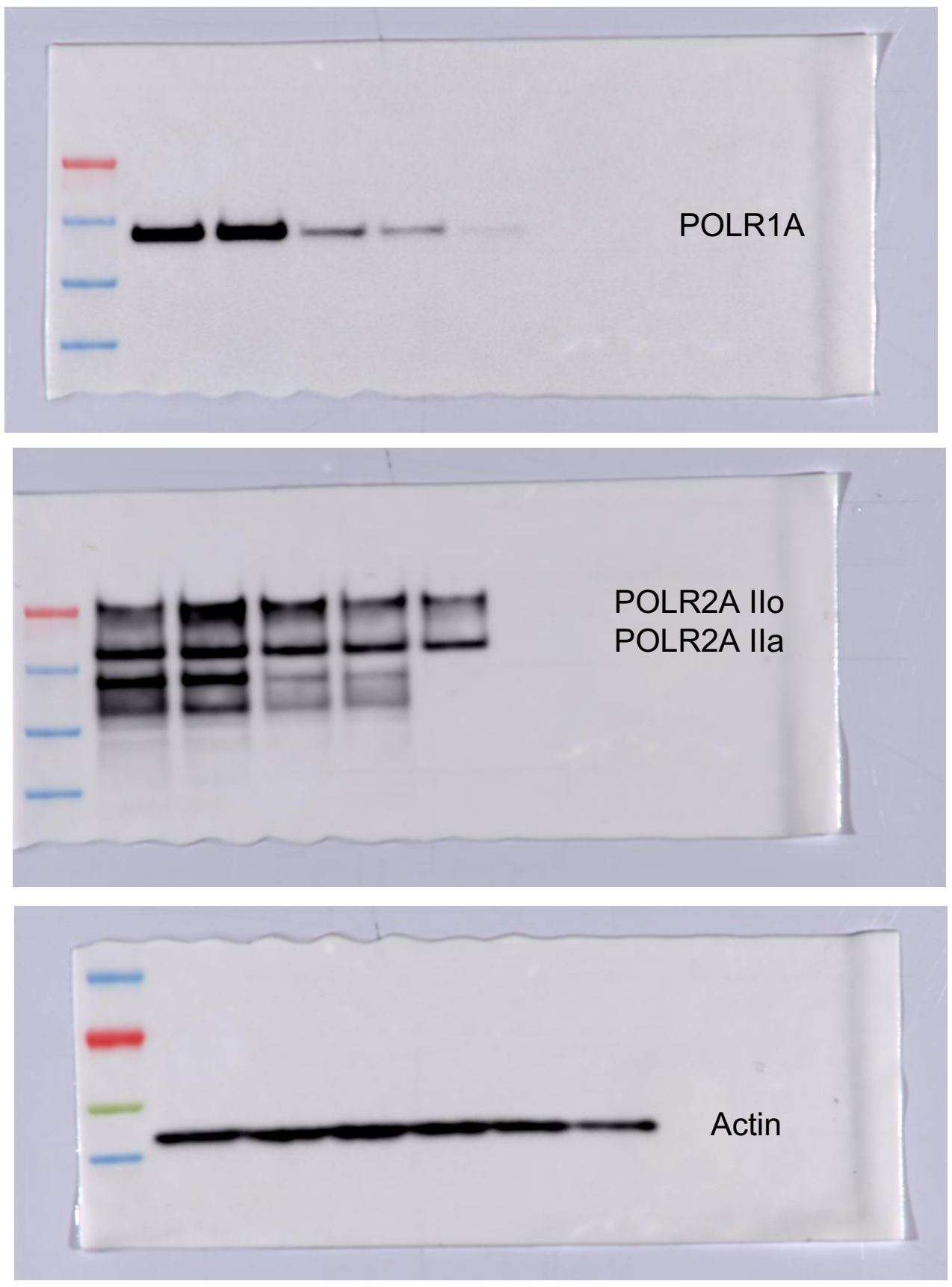

HCT116 3h

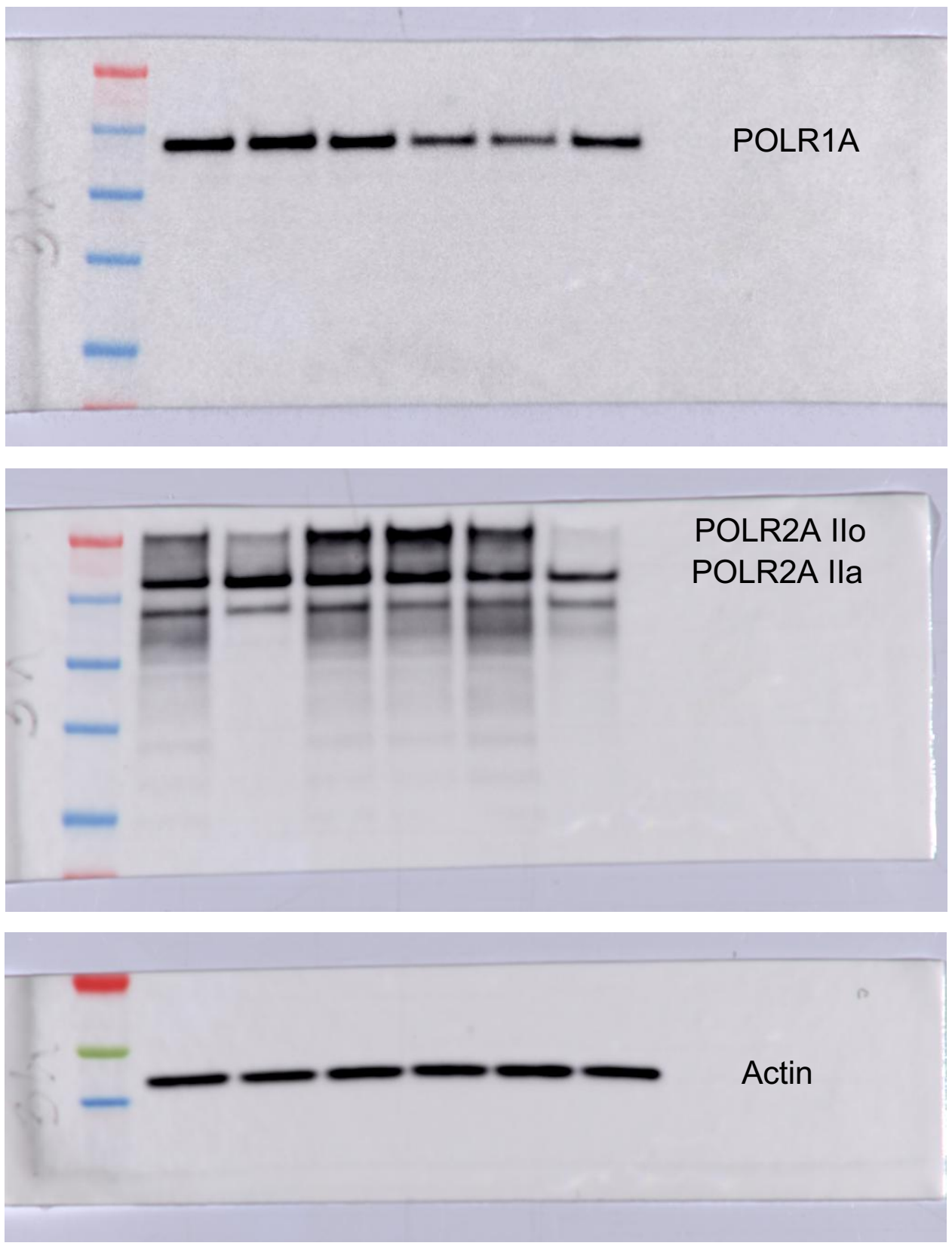

HCT116 24h

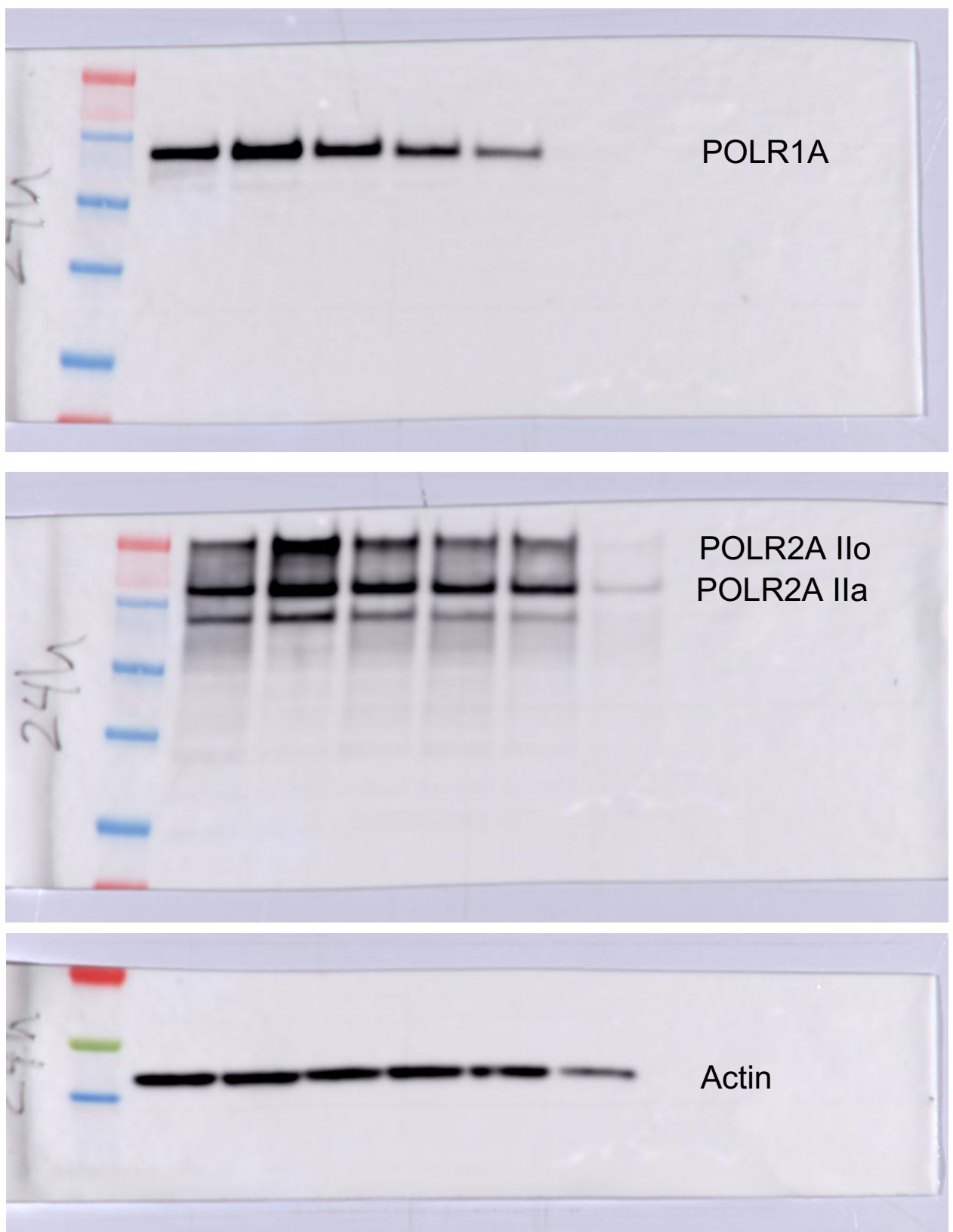

ddp53 U2OS POLR1A 3h

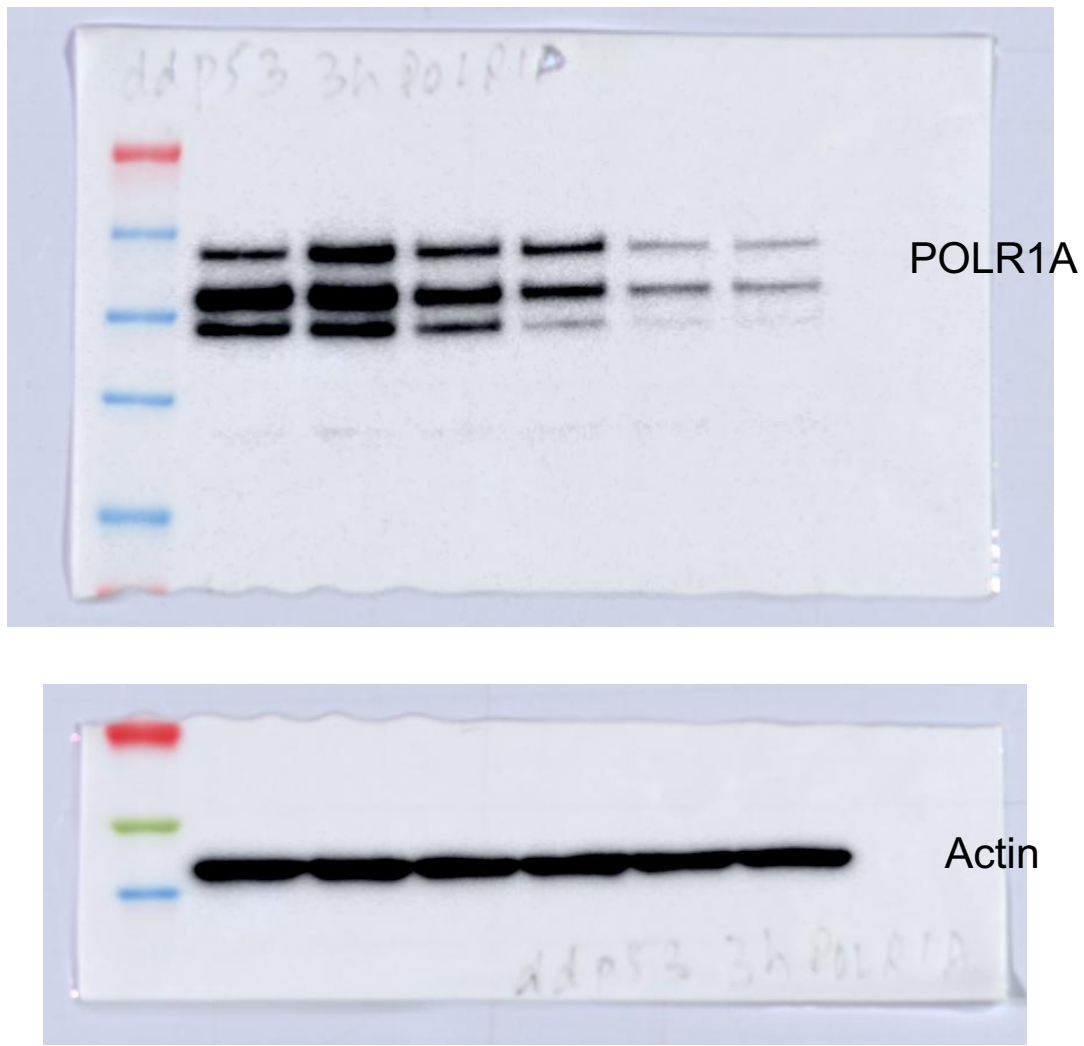

ddp53 U2OS POLR2A 3h

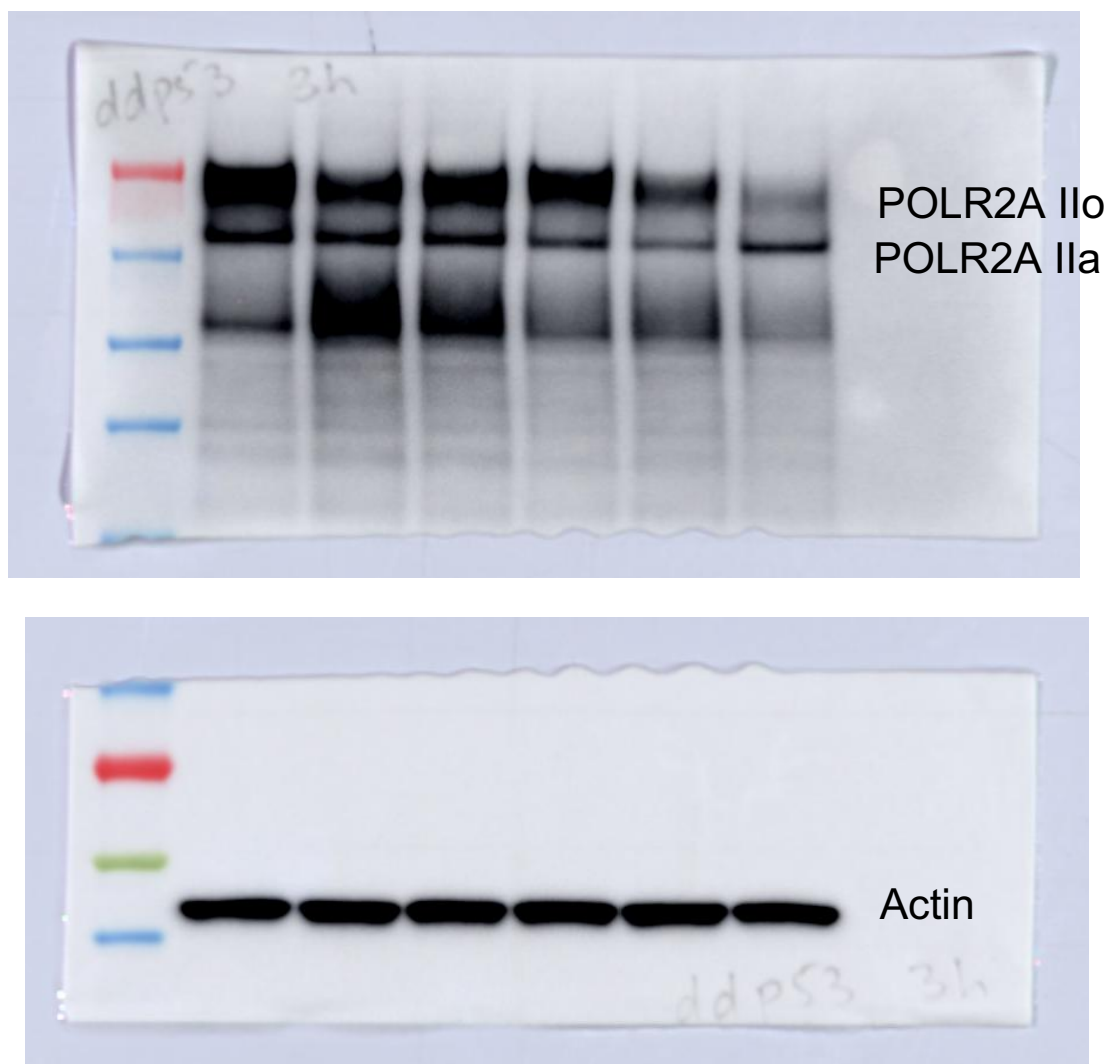

ddp53 U2OS POLR1A 24h

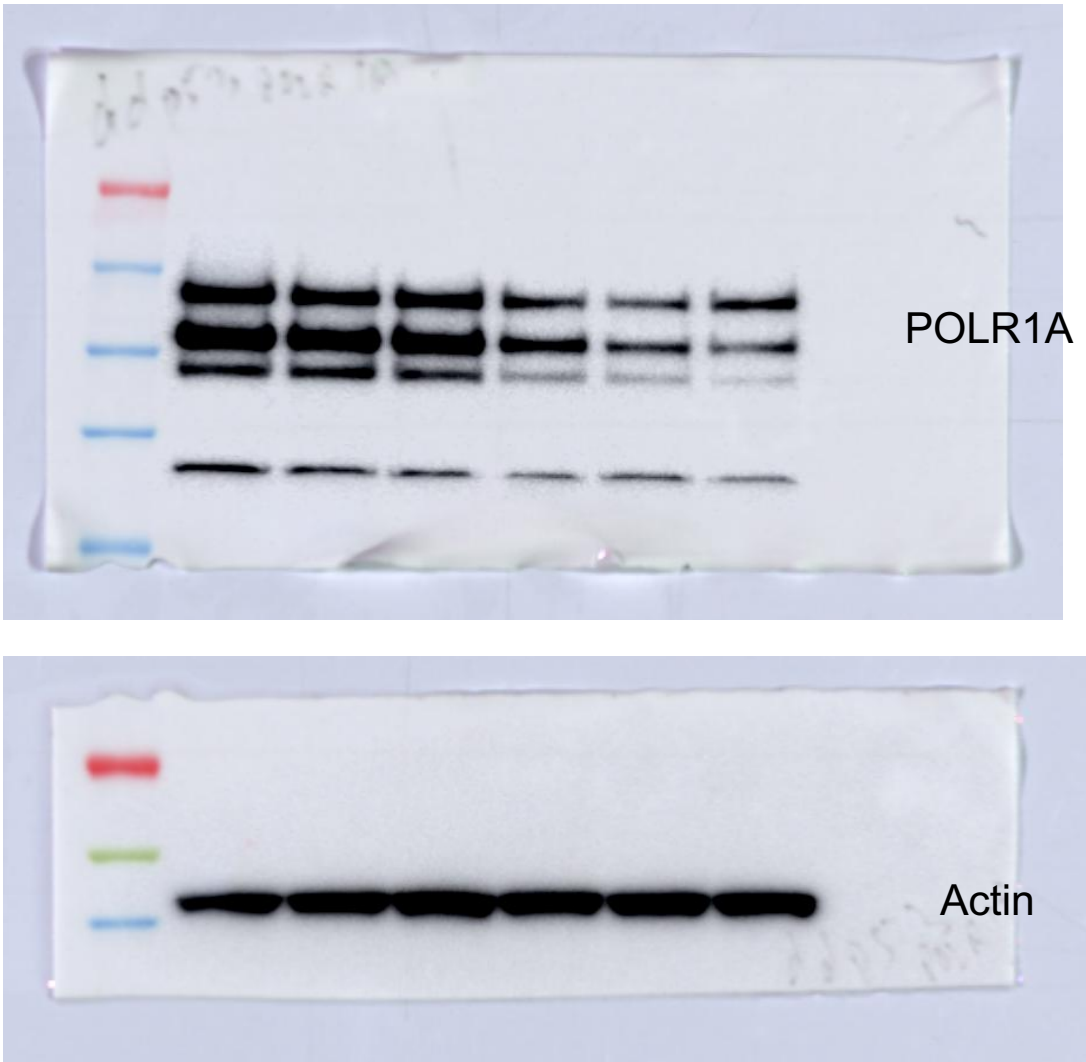

ddp53 U2OS POLR2A 24h

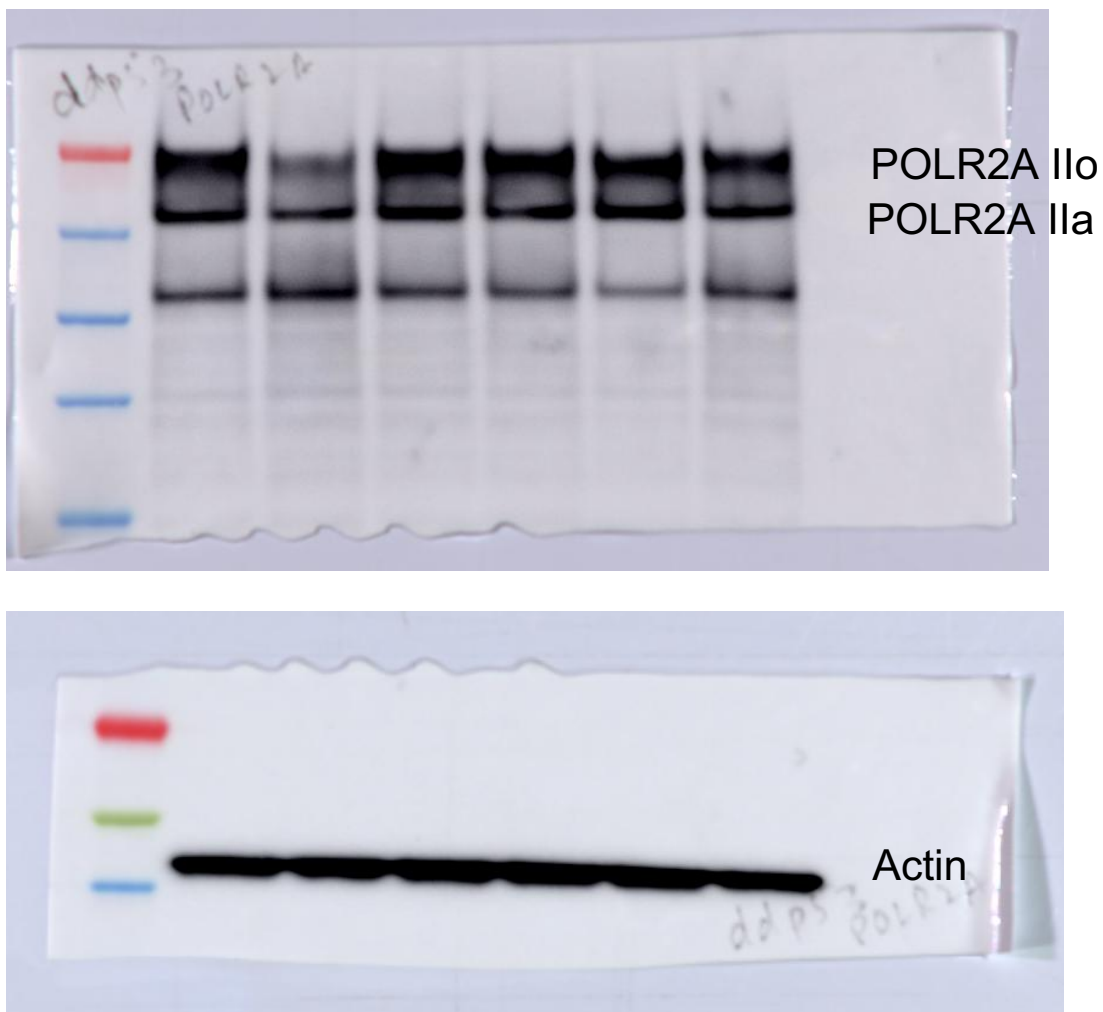

A172 POLR1A 3h

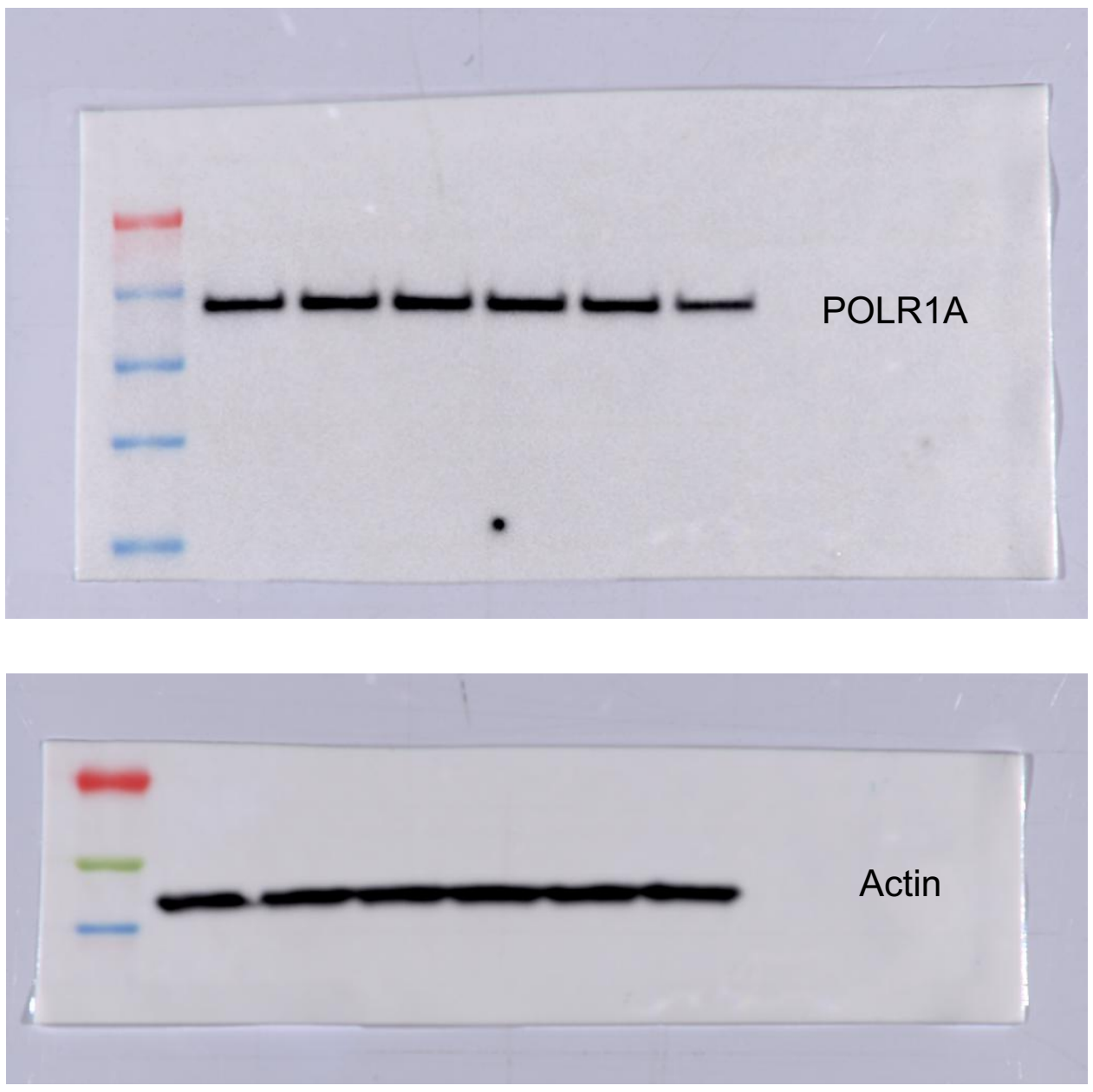

A172 POLR2A 3h

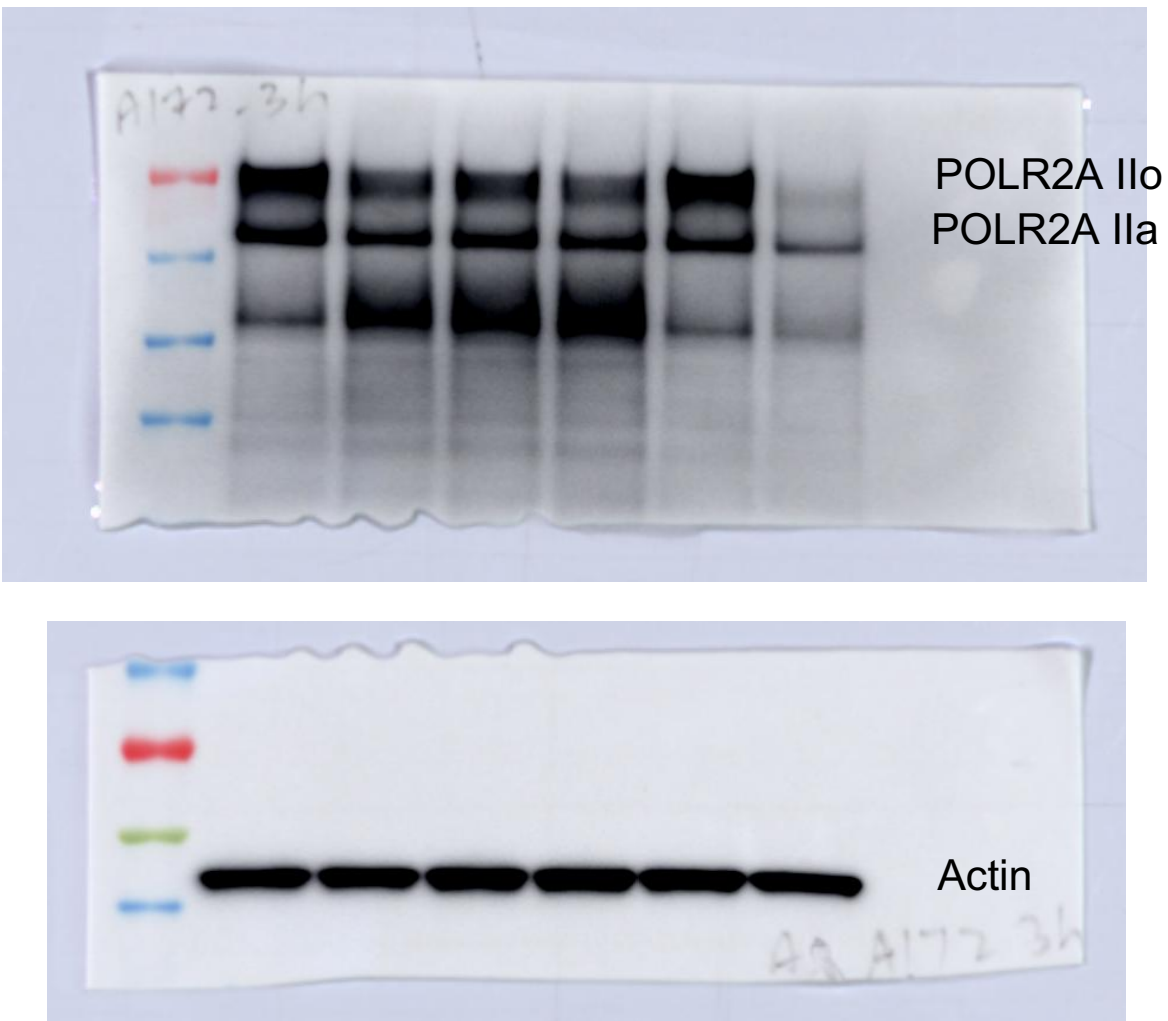

A172 POLR1A 24h

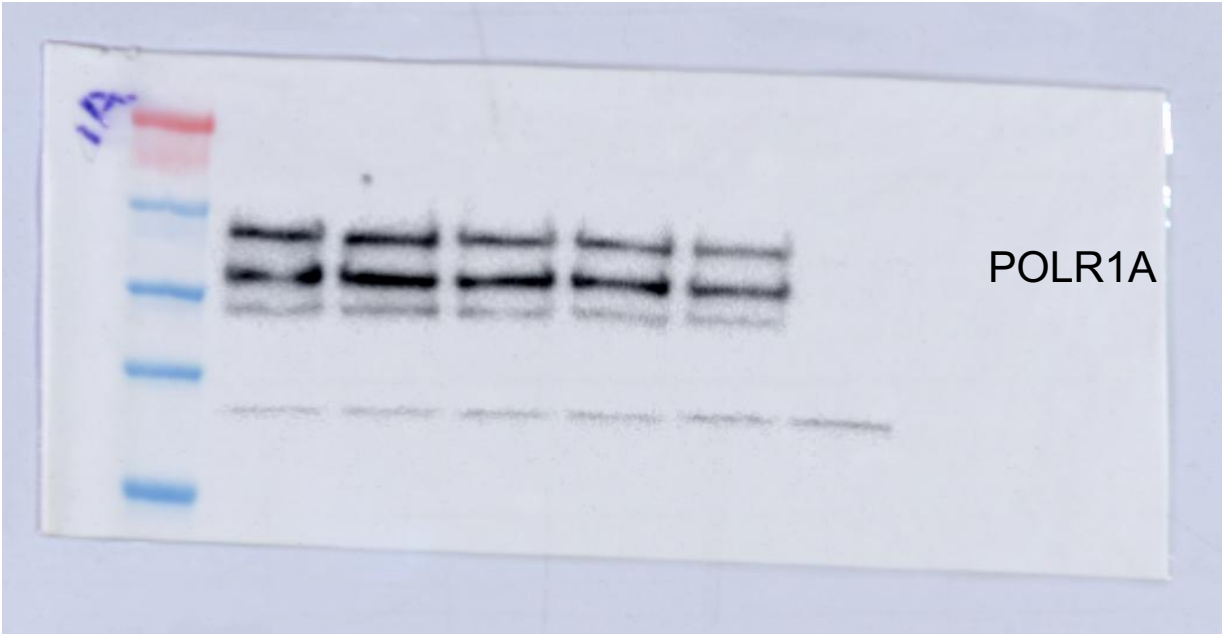

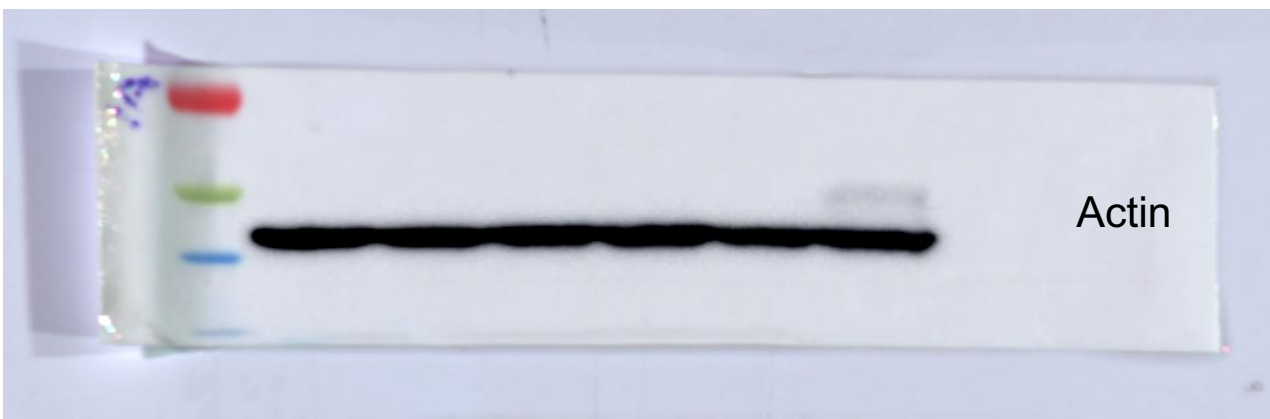

A172 POLR2A 24h

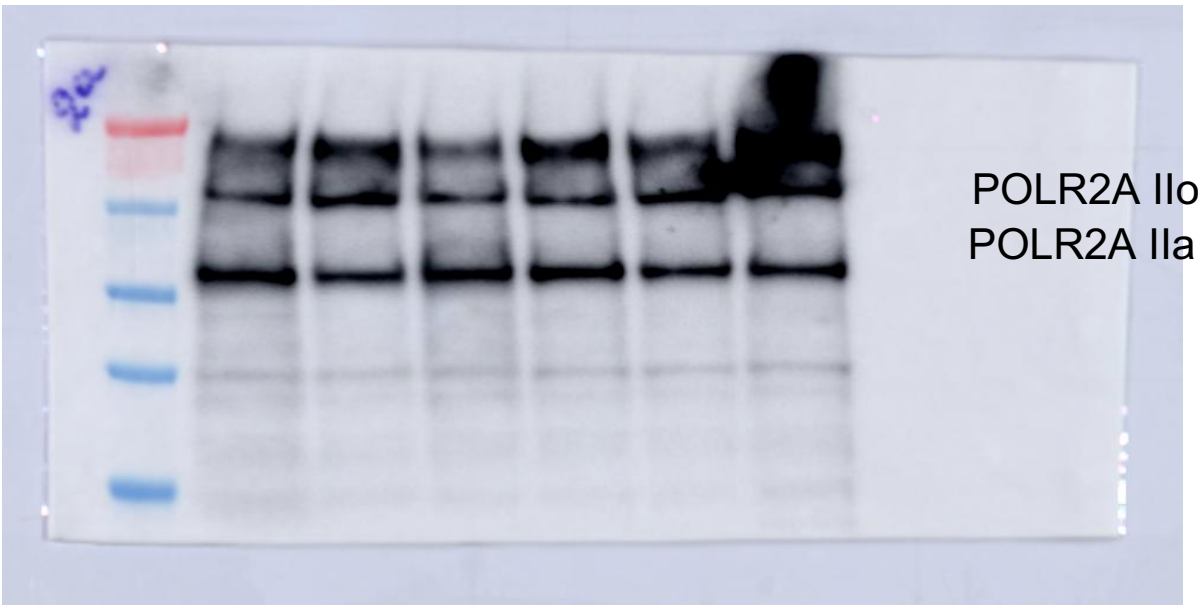

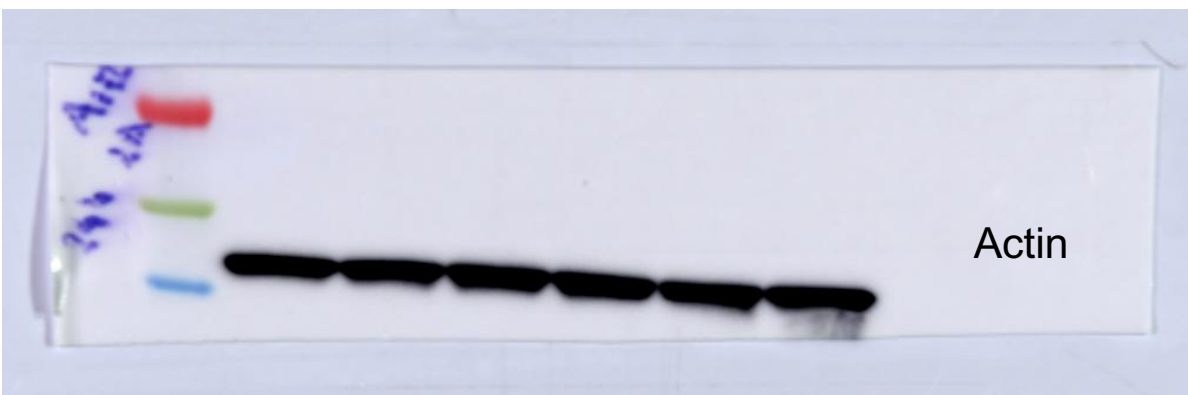

RKO POLR1A 3h

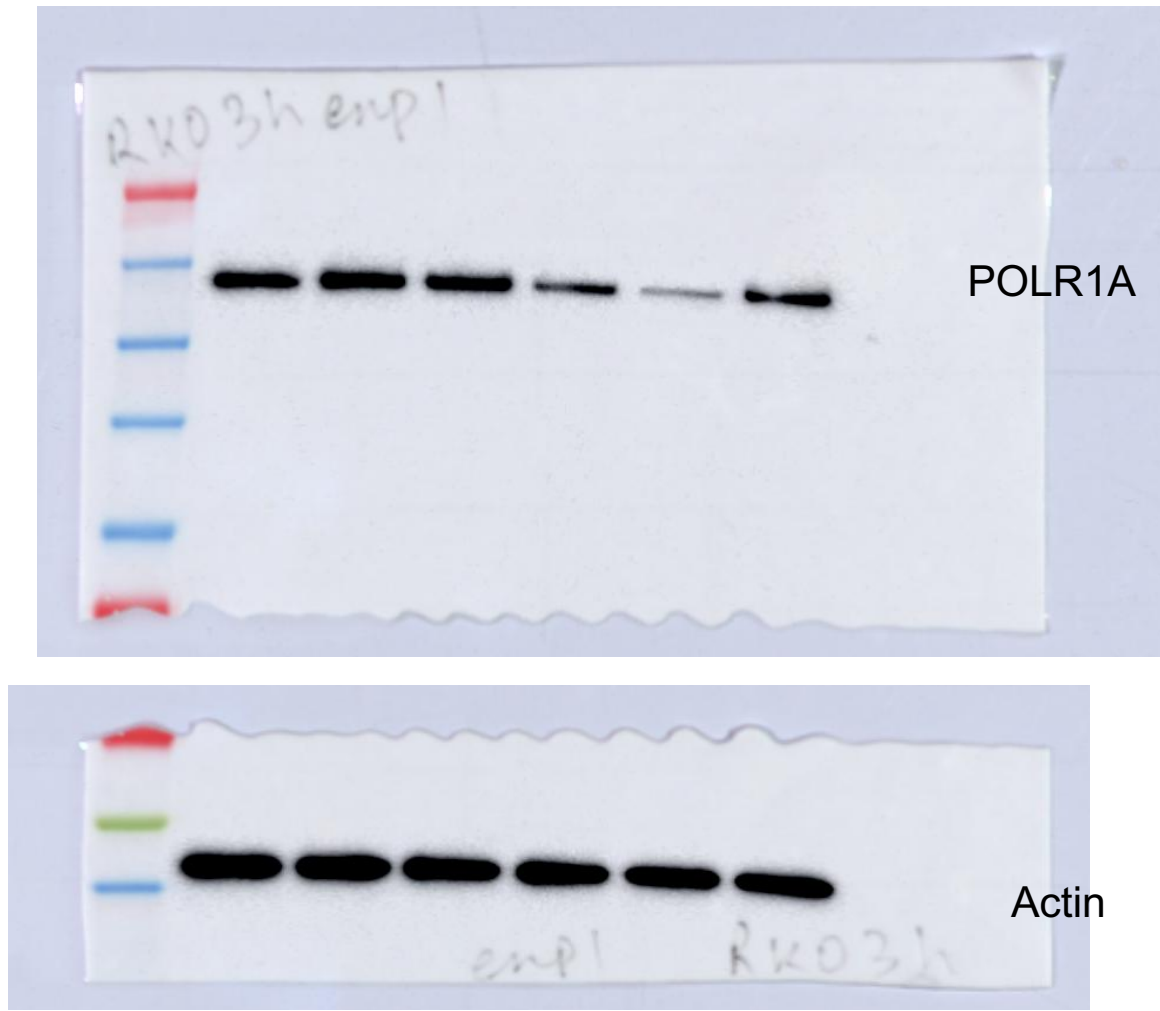

RKO POLR2A 3h

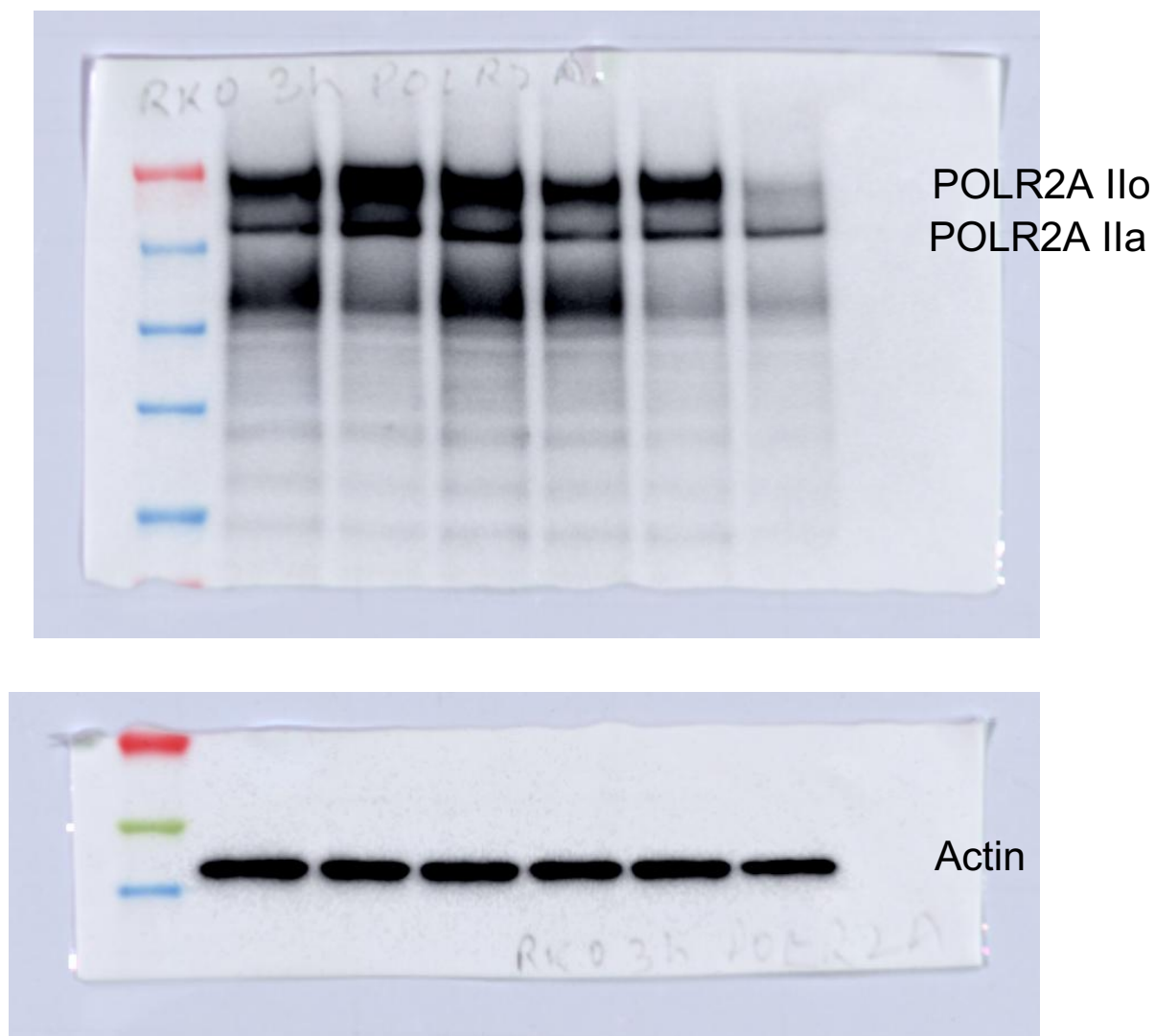

RKO POLR1A 24h

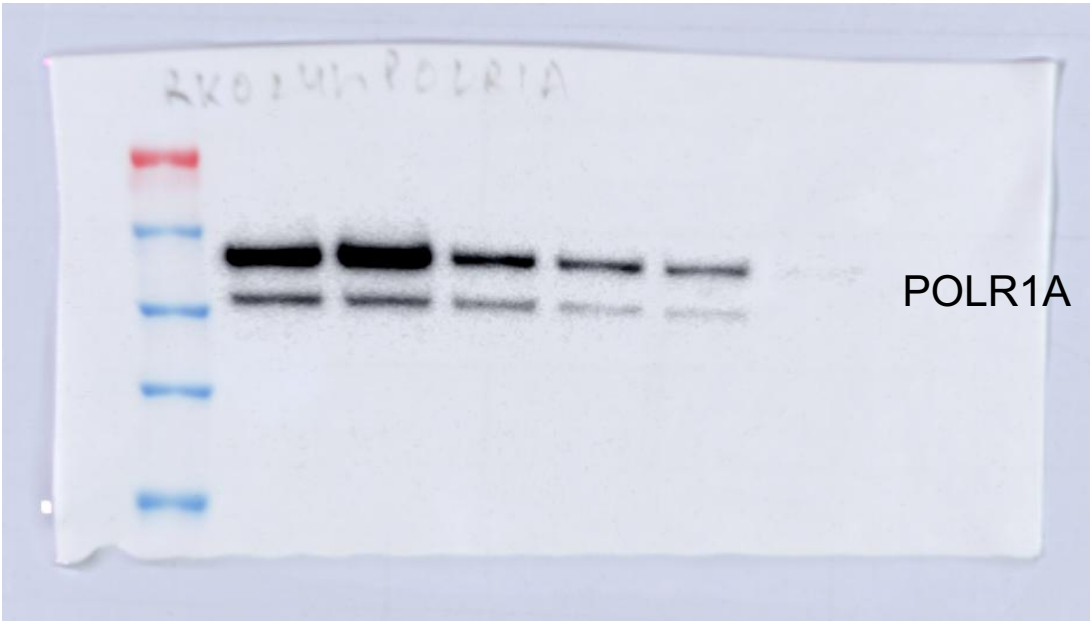

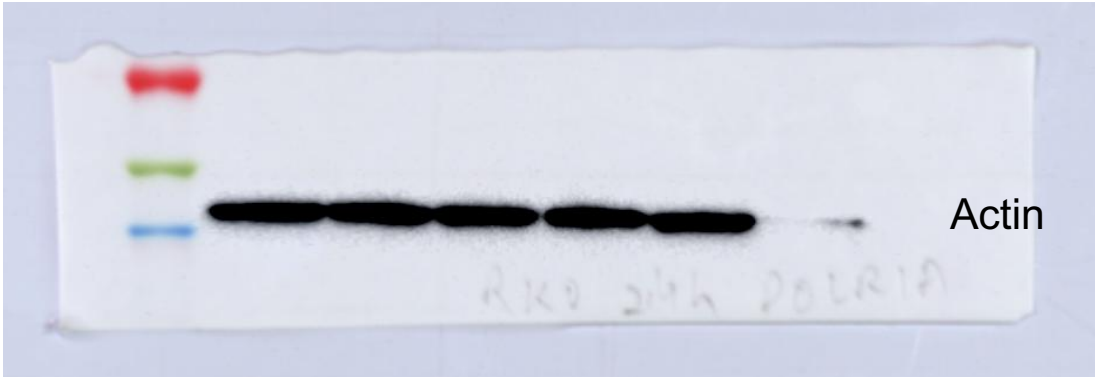

RKO POLR2A 24h

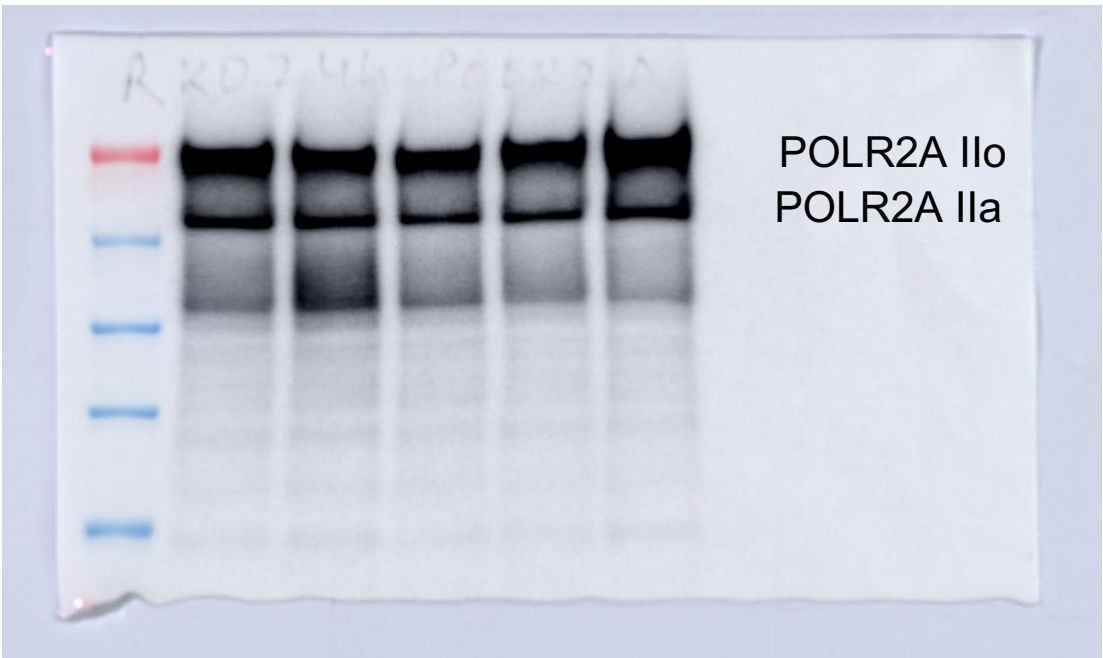

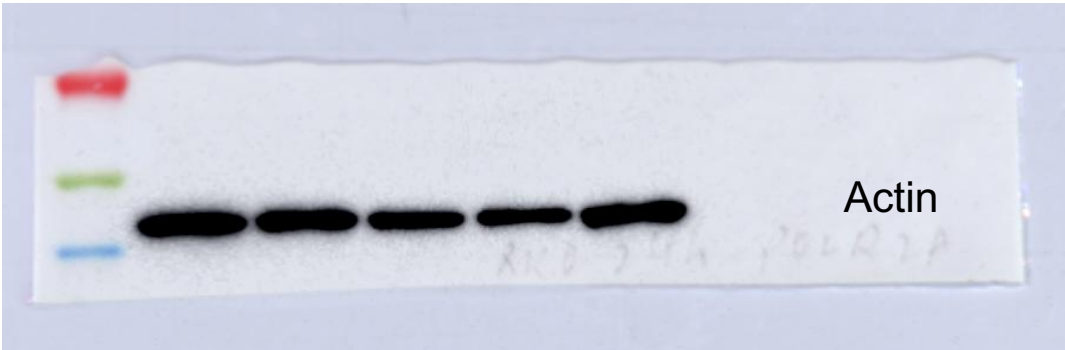

LN229 POLR1A 3h

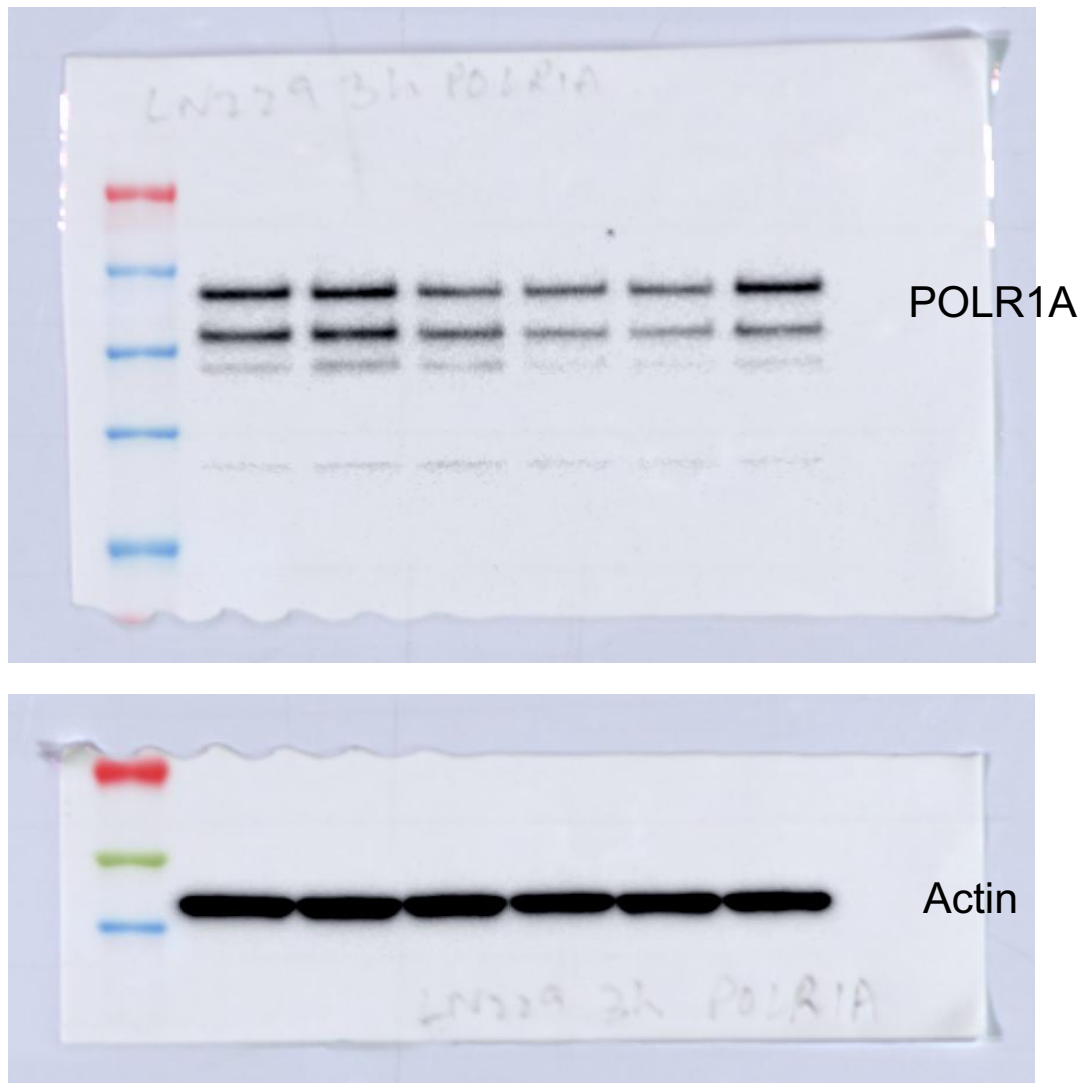

LN229 POLR2A 3h

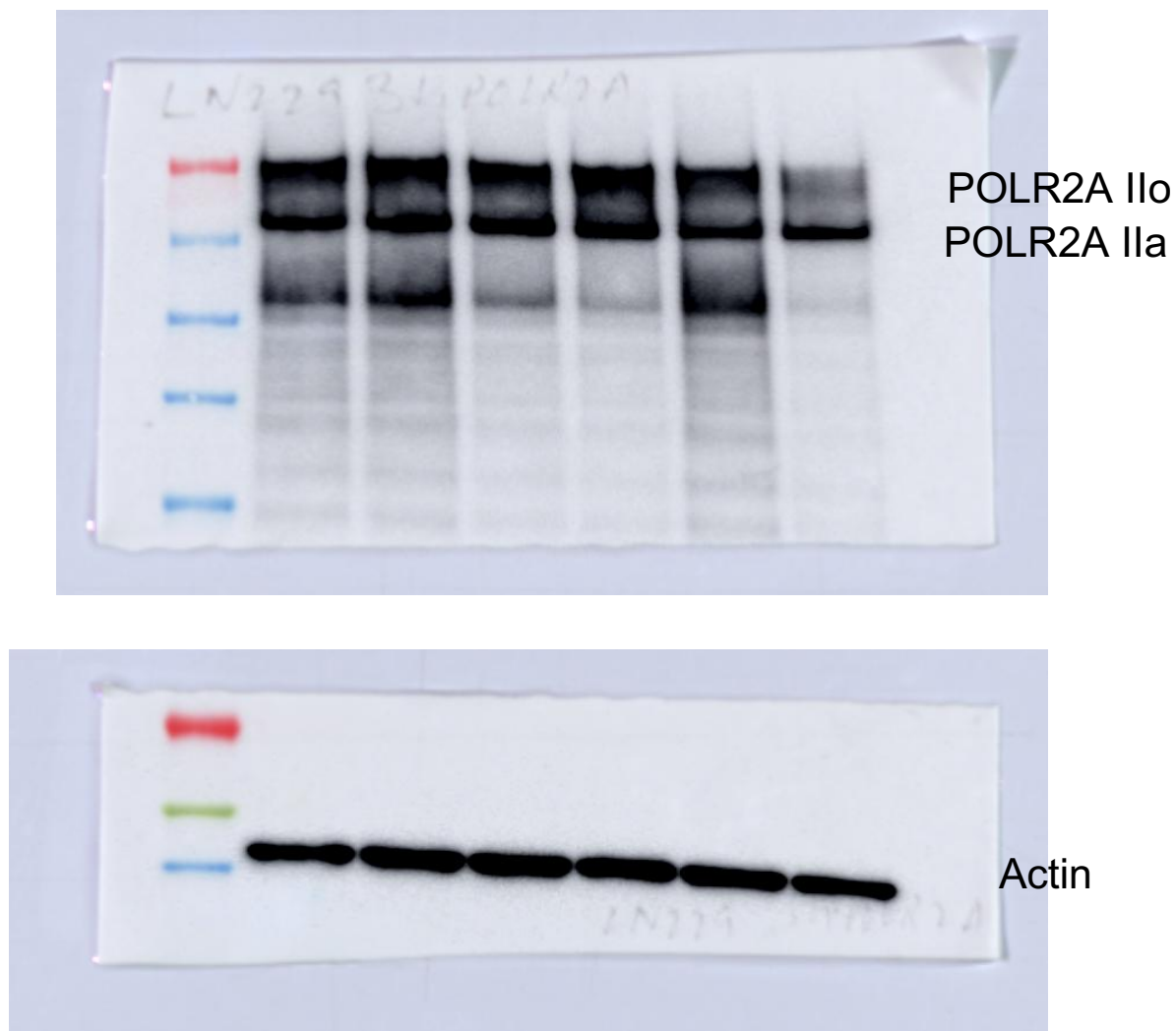

LN229 POLR1A 24h

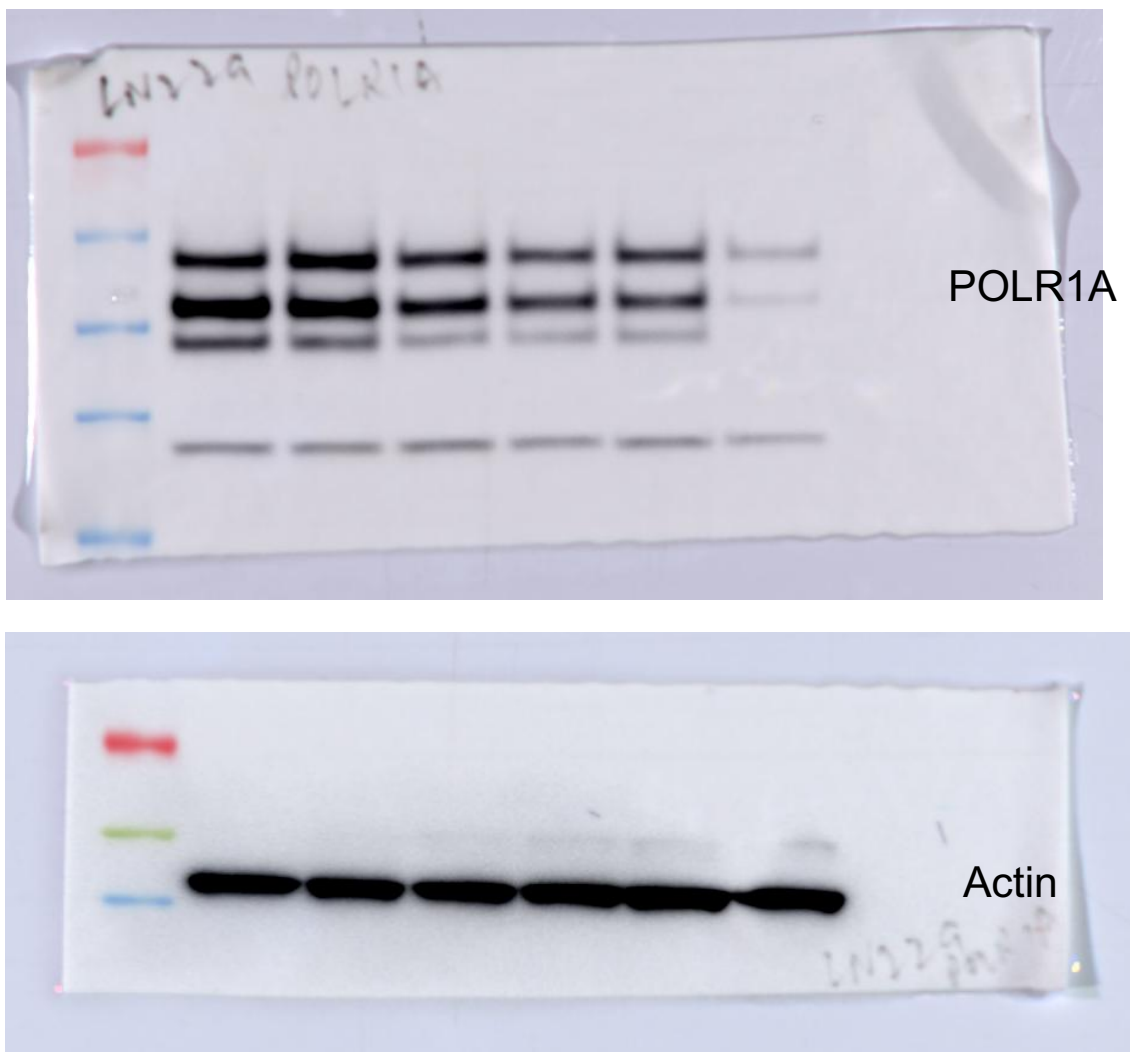

LN229 POLR2A 24h

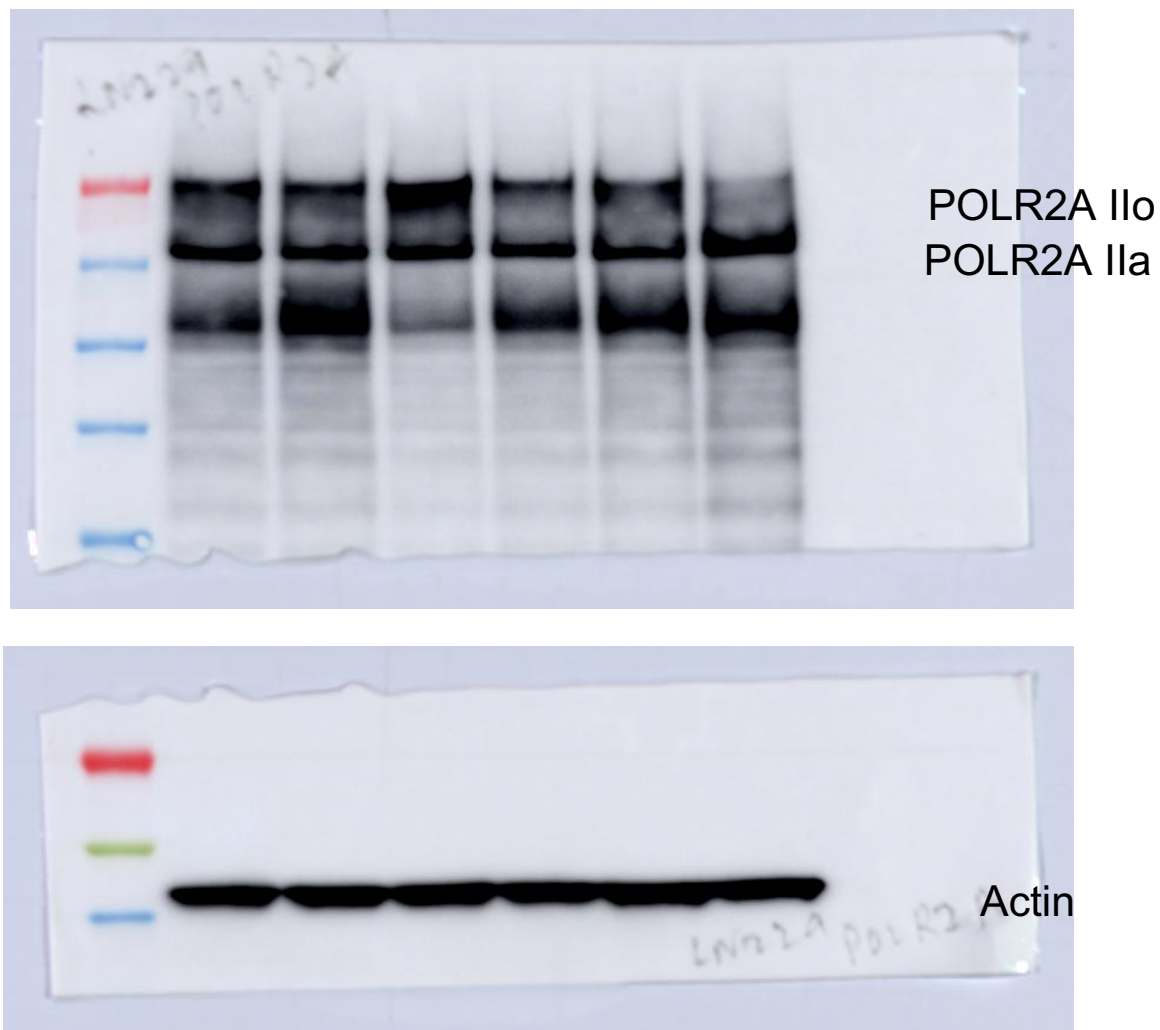

Figure 1D

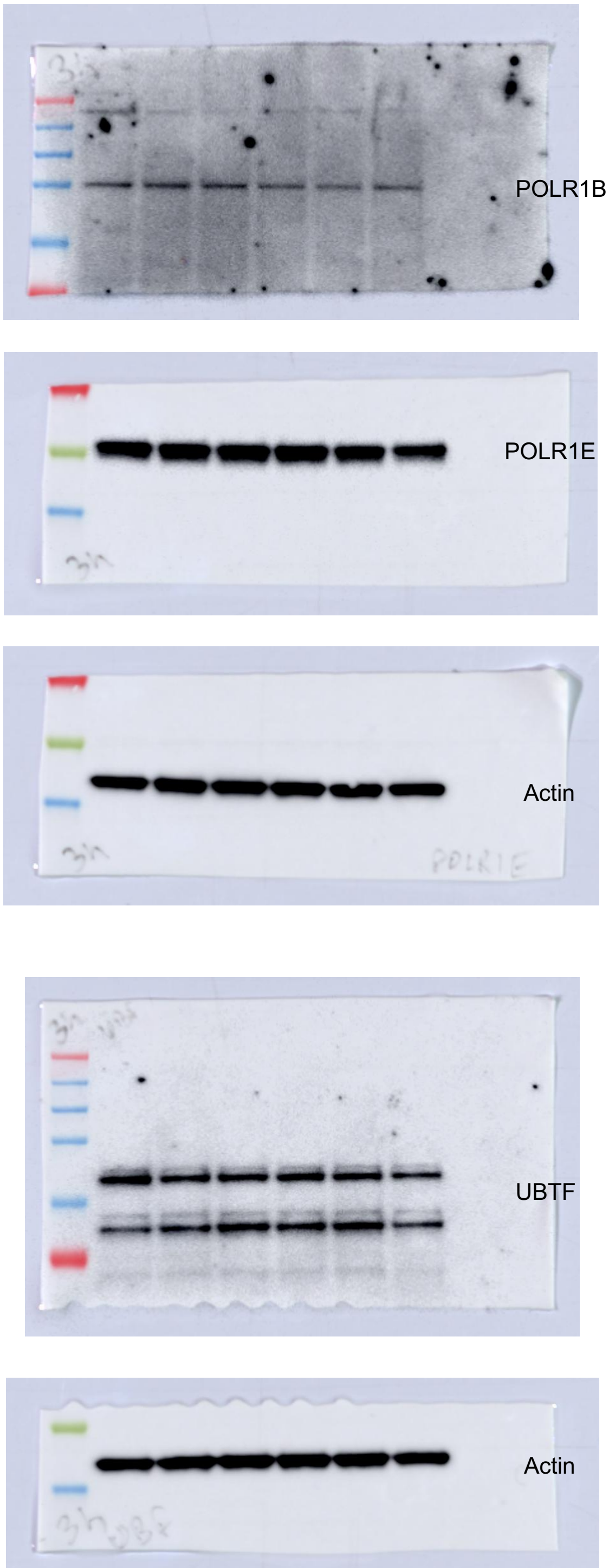

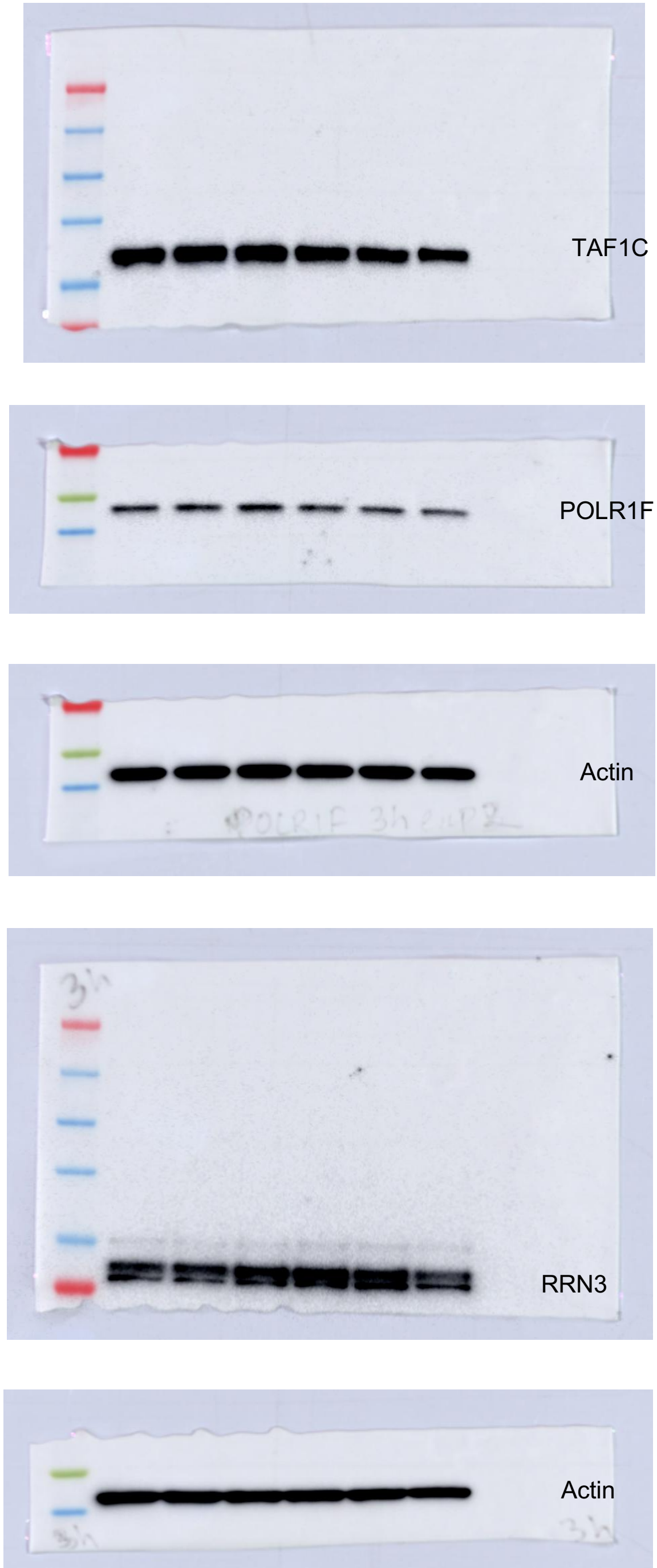
TAF1C
POLR1F
Actin
RRN3
Actin

Figure 1E

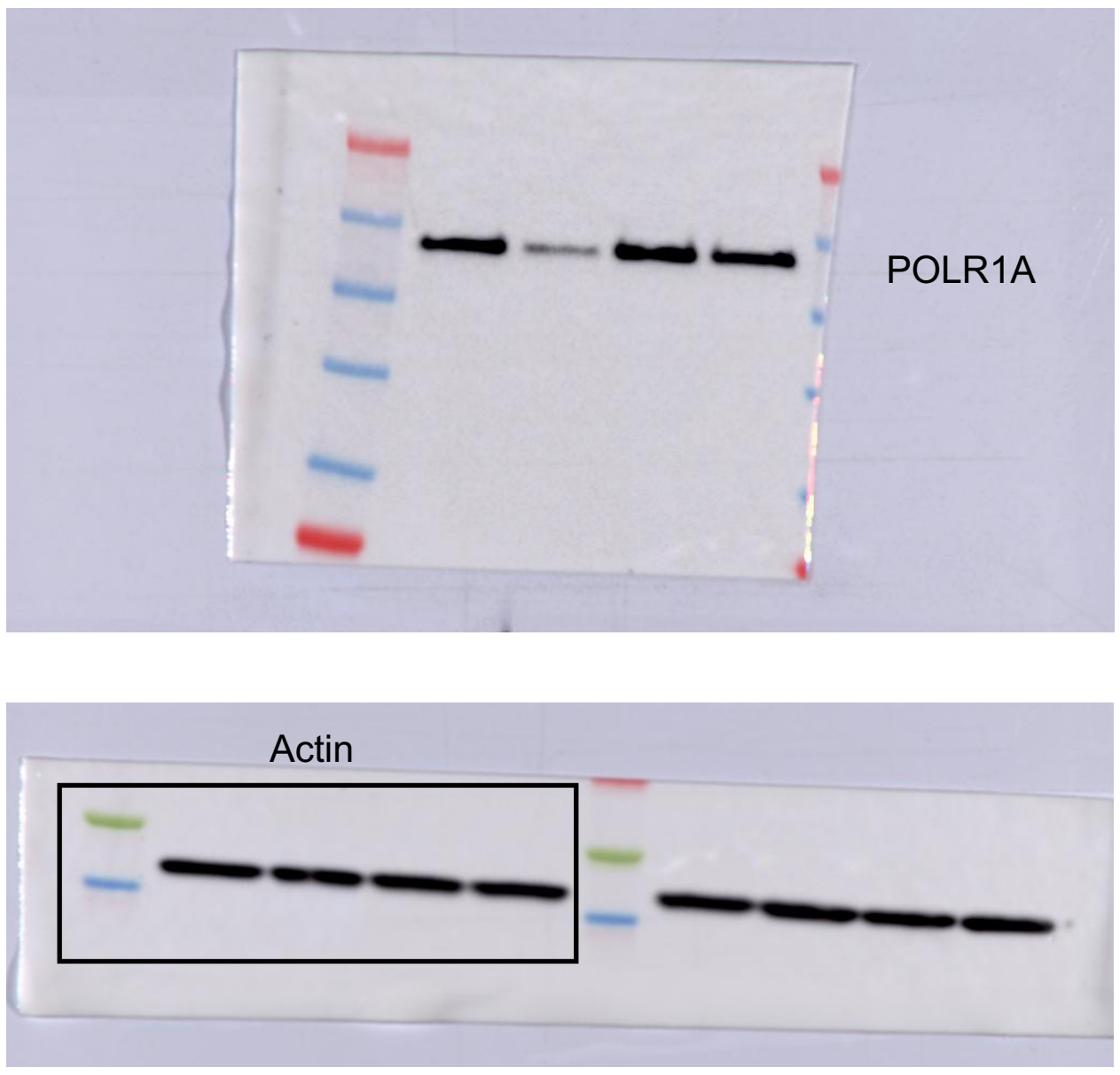

Figure 4A

U2OS 3h

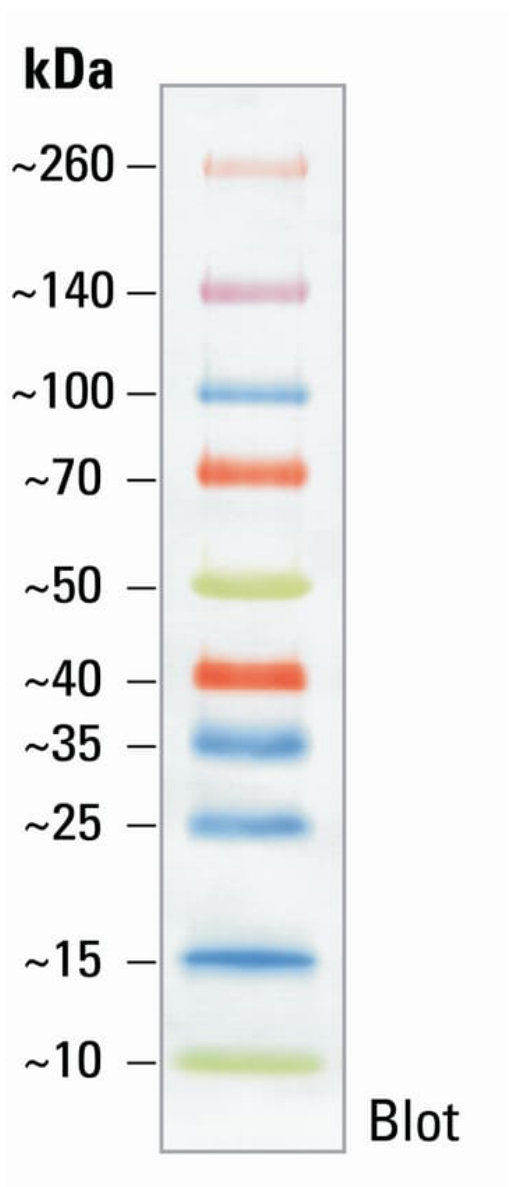

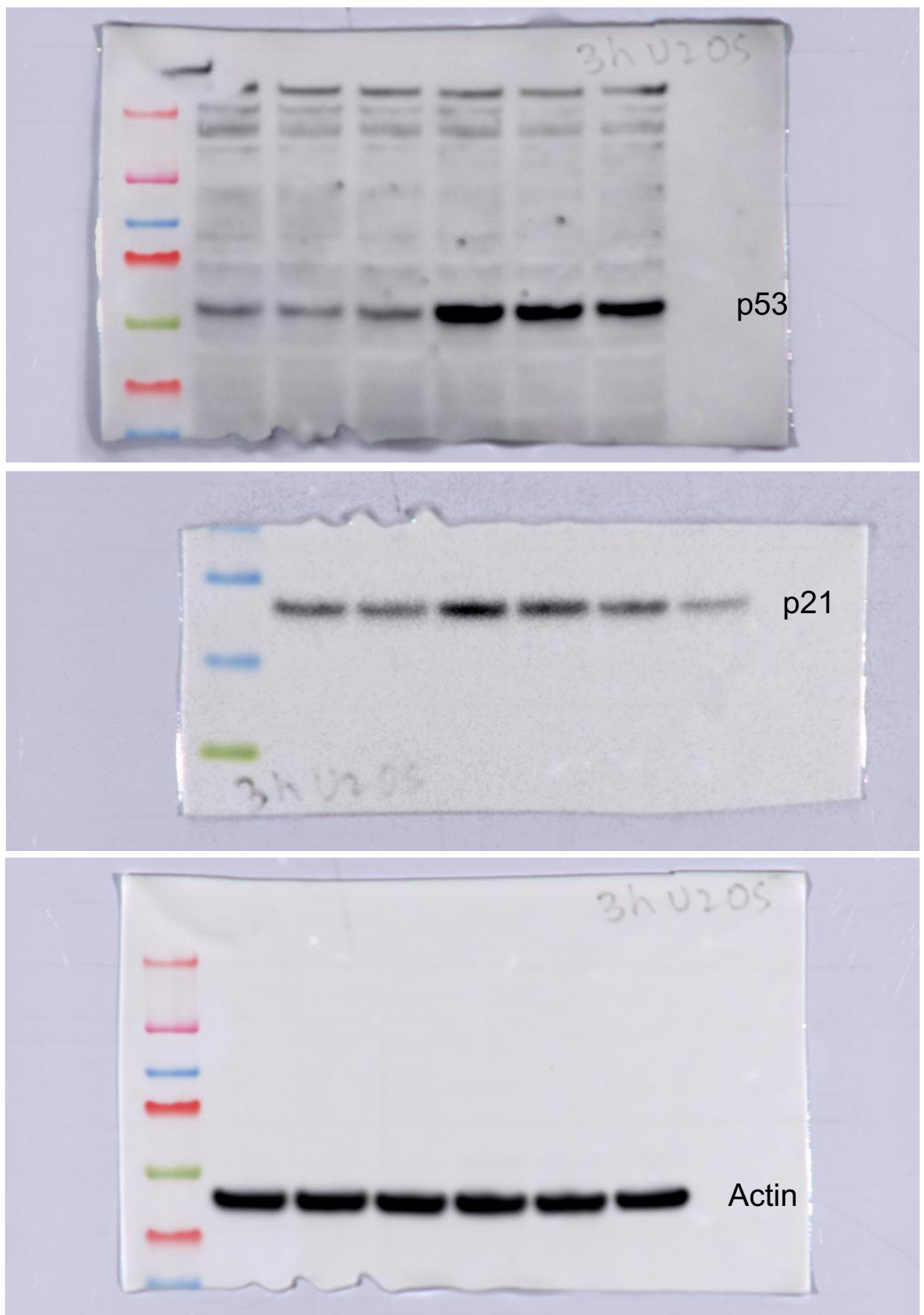

U2OS 24h

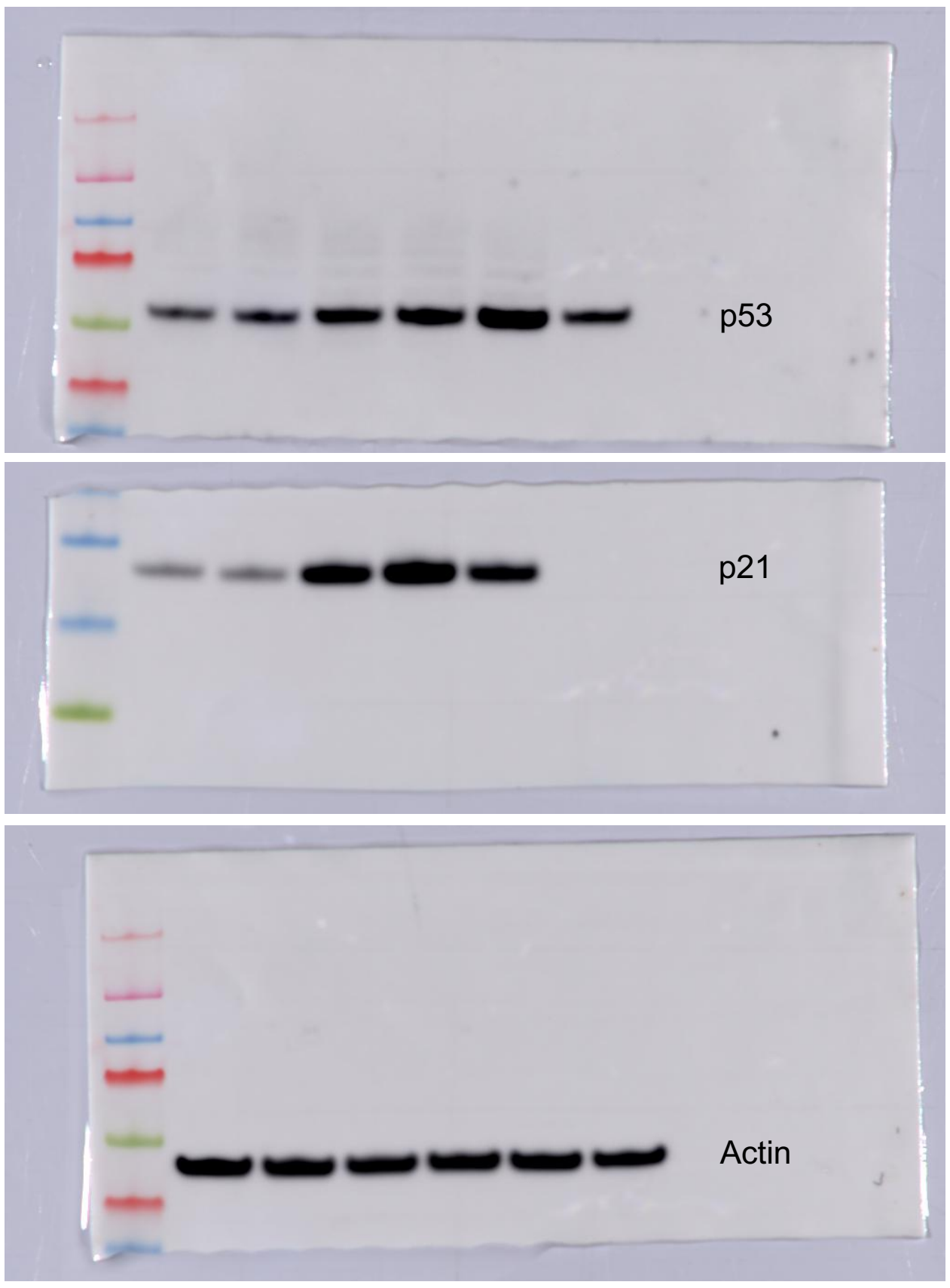

ddp53U2OS 3h

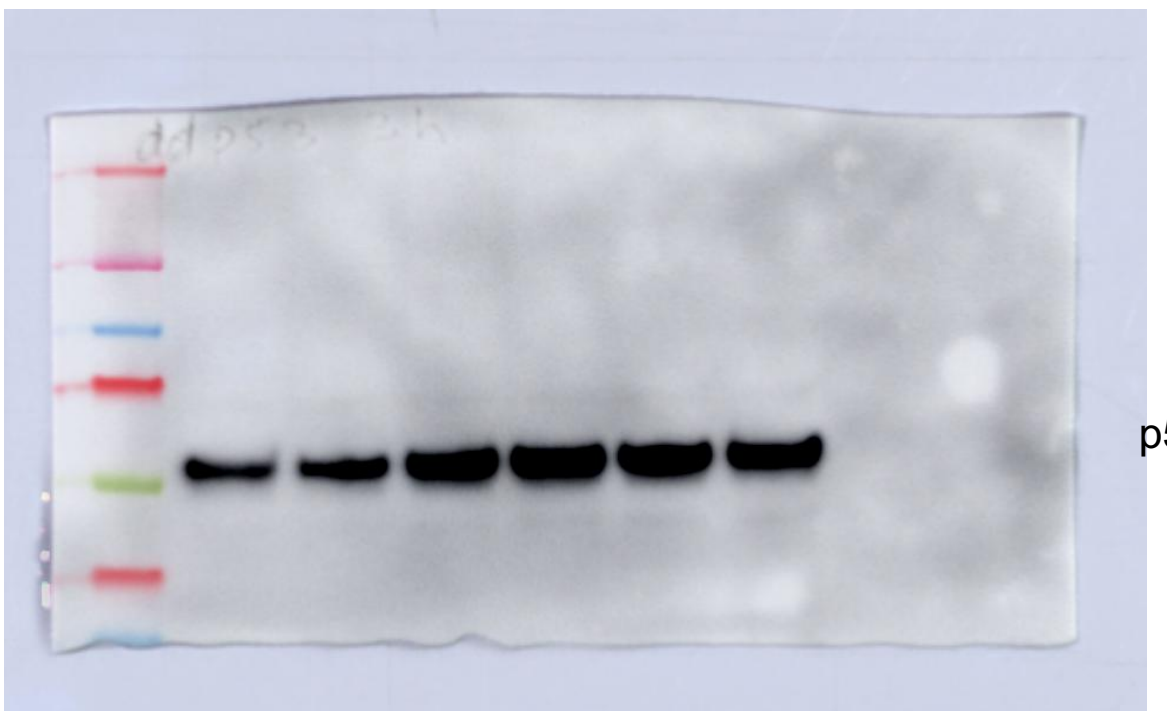

p53

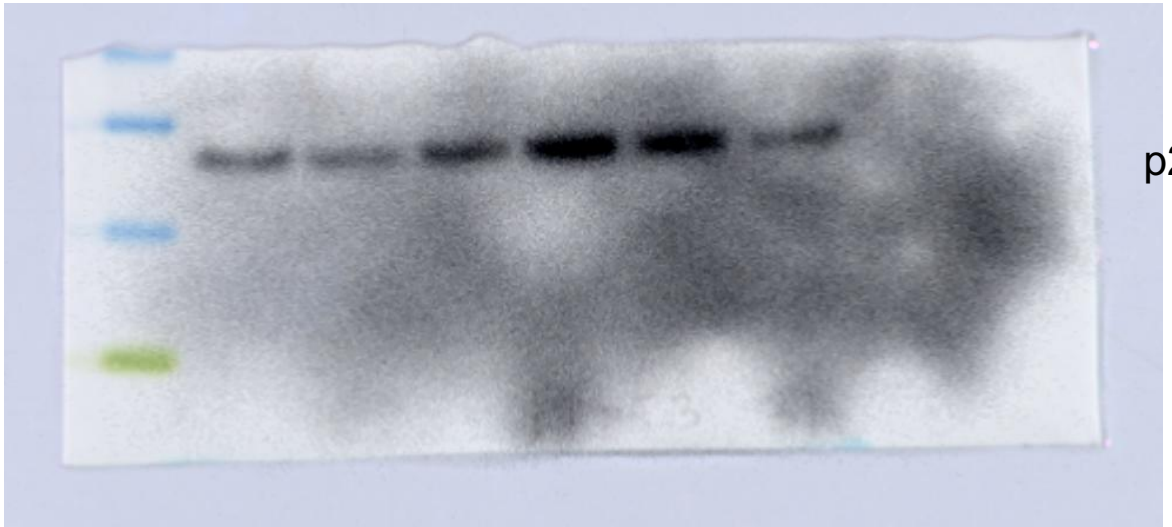

p21

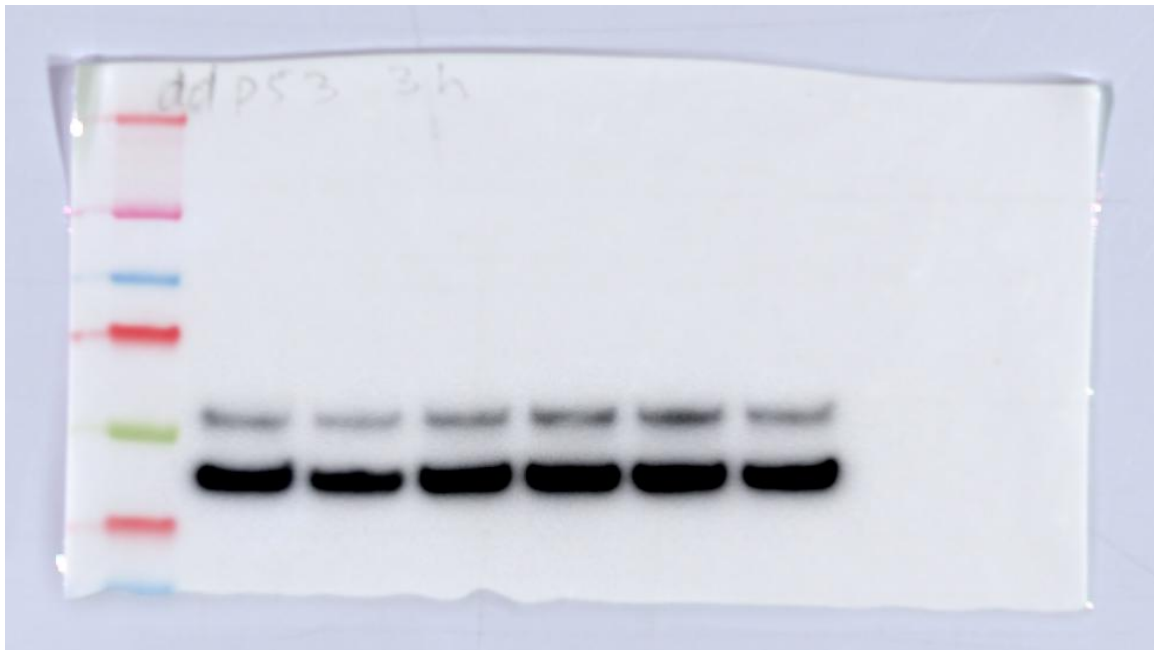

Actin

ddp53U2OS 24h

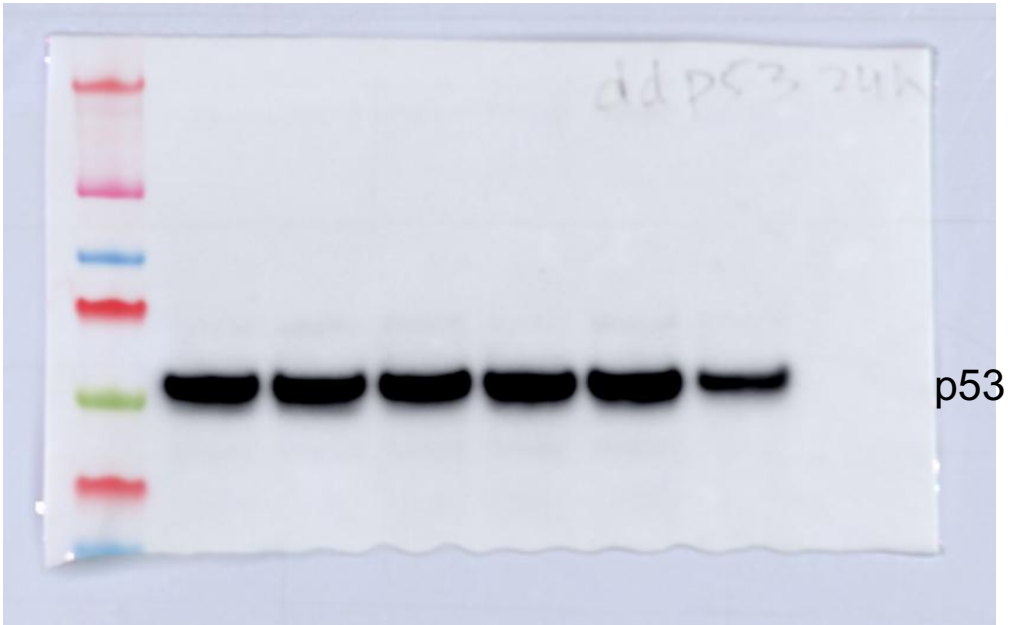

p53

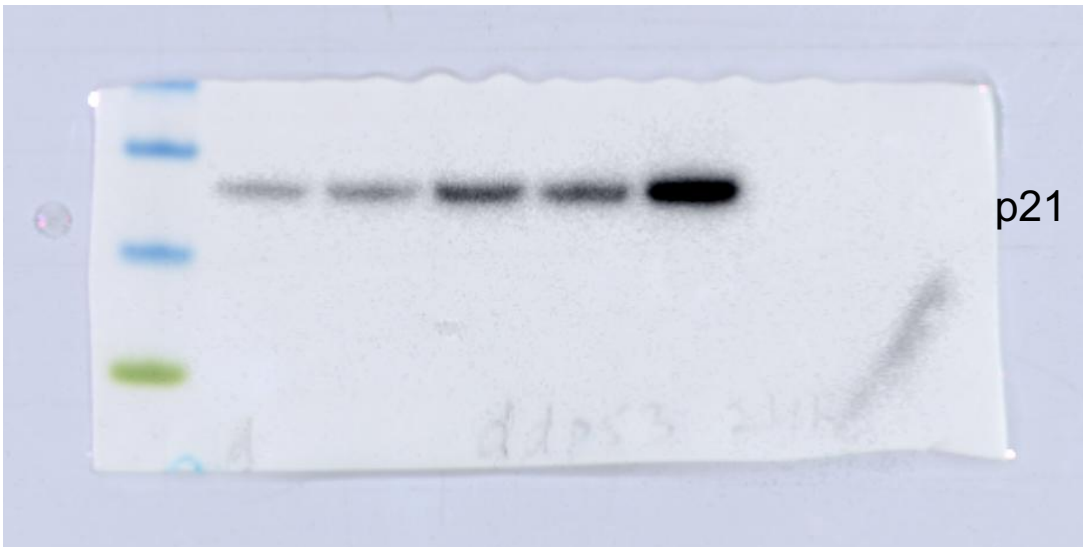

p21

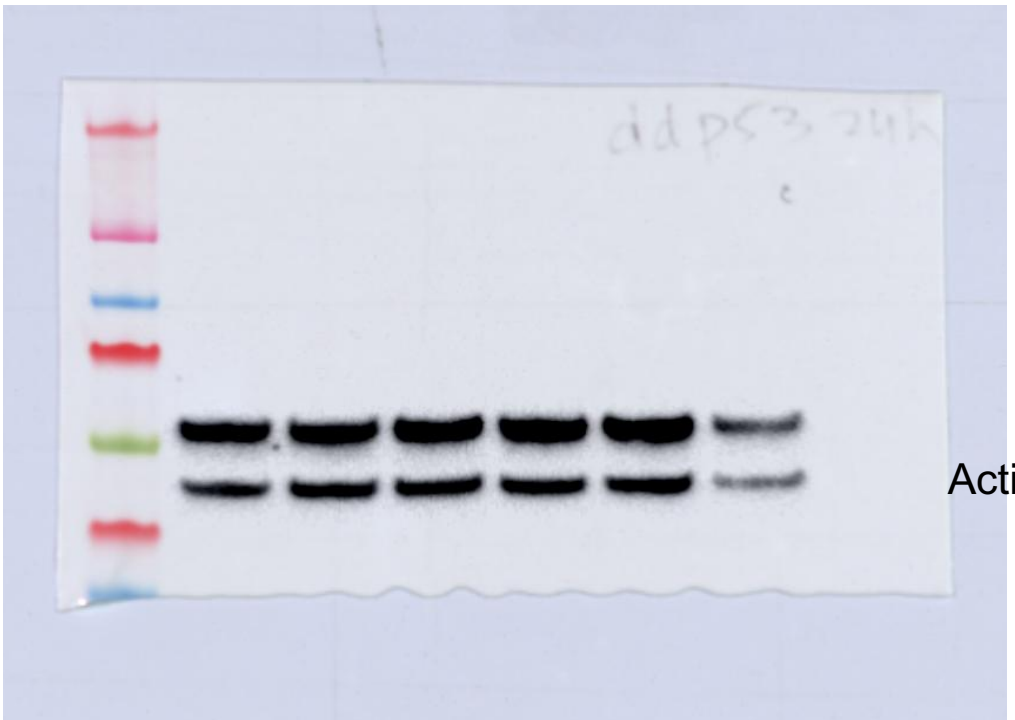

Actin

LN229 3h

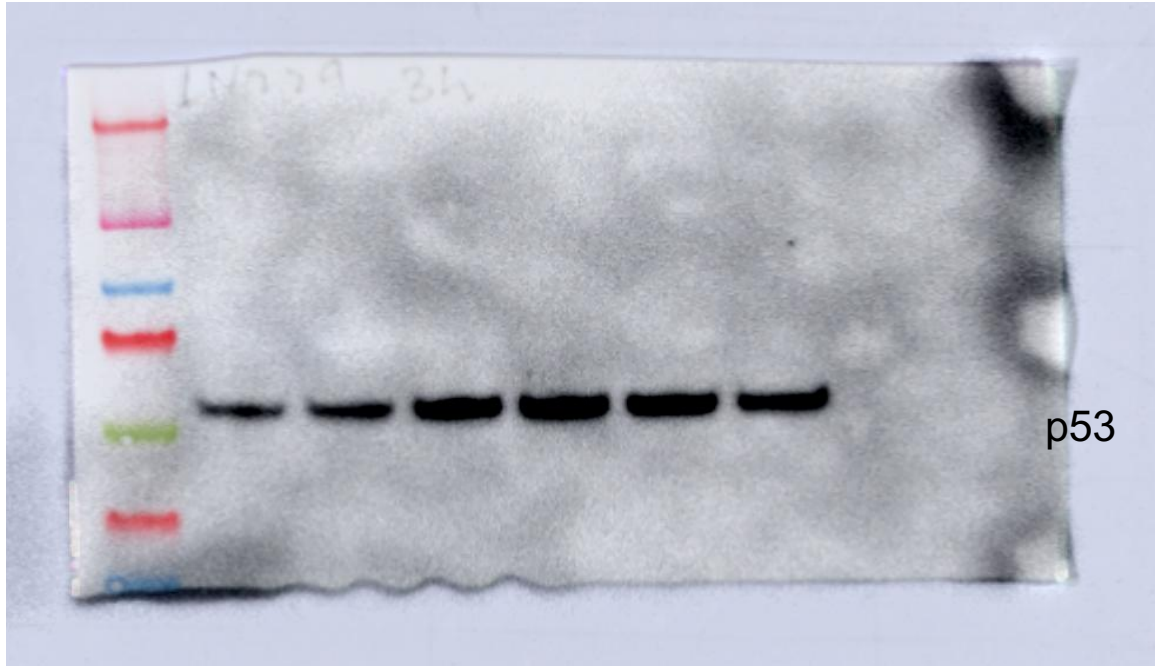

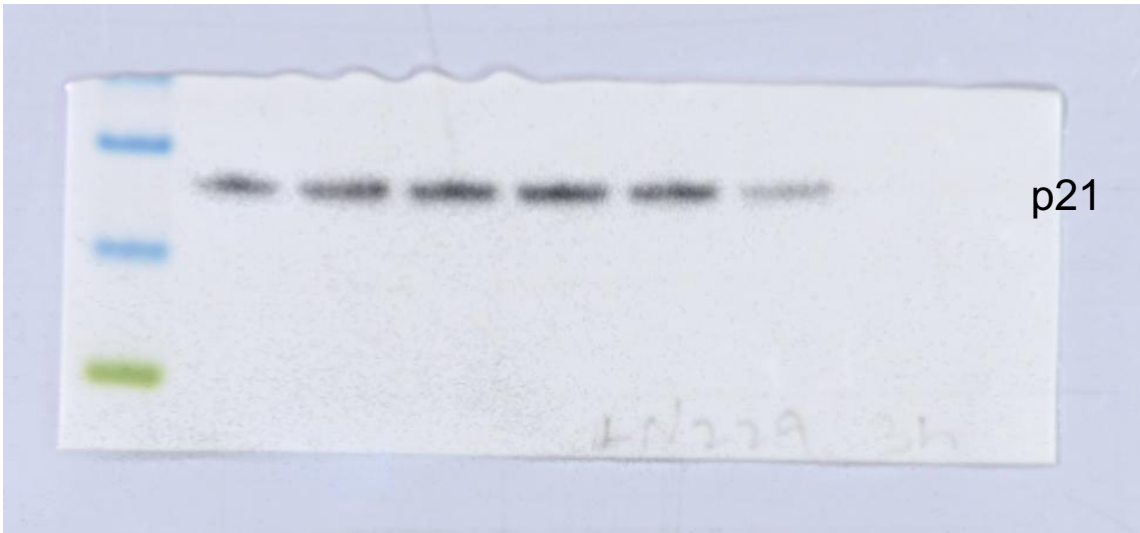

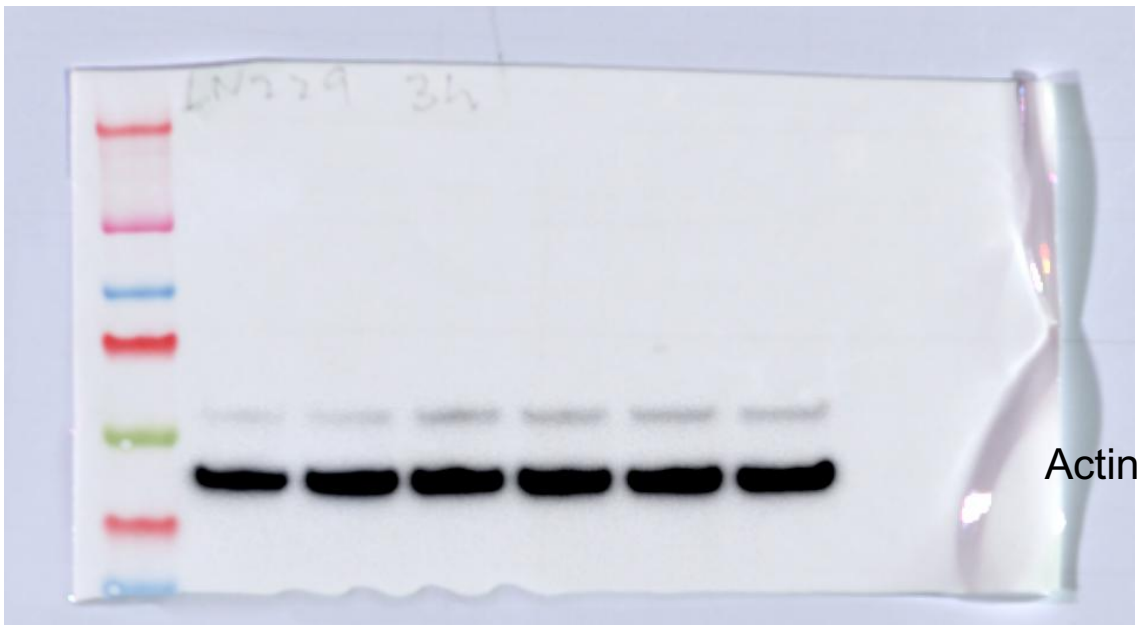

LN229 24h

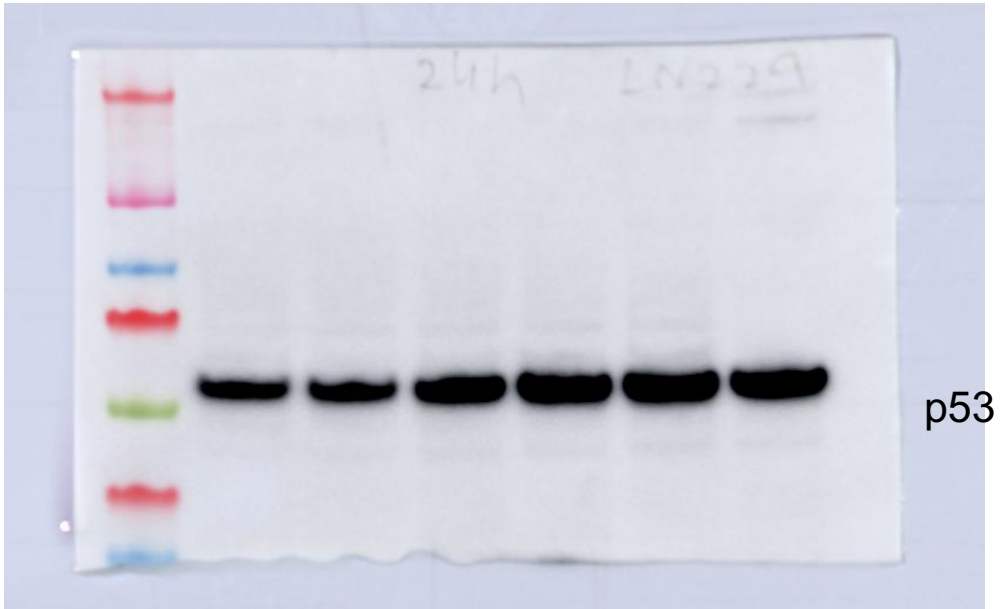

p53

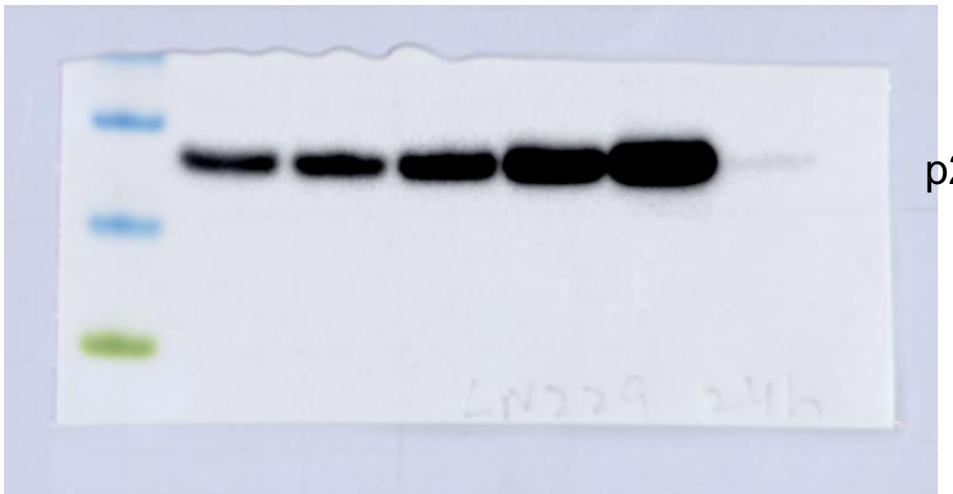

p21

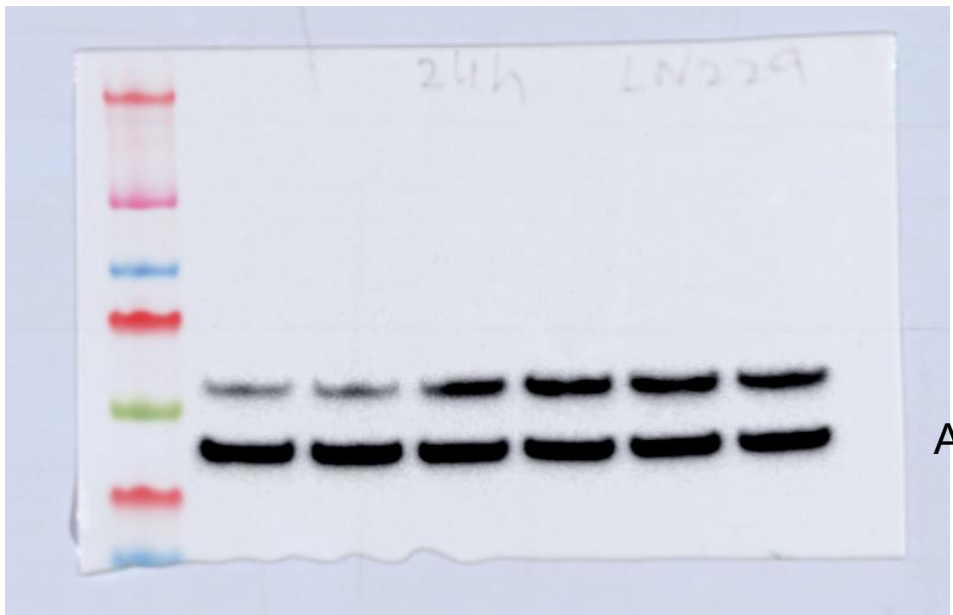

Actin

RKO 3h

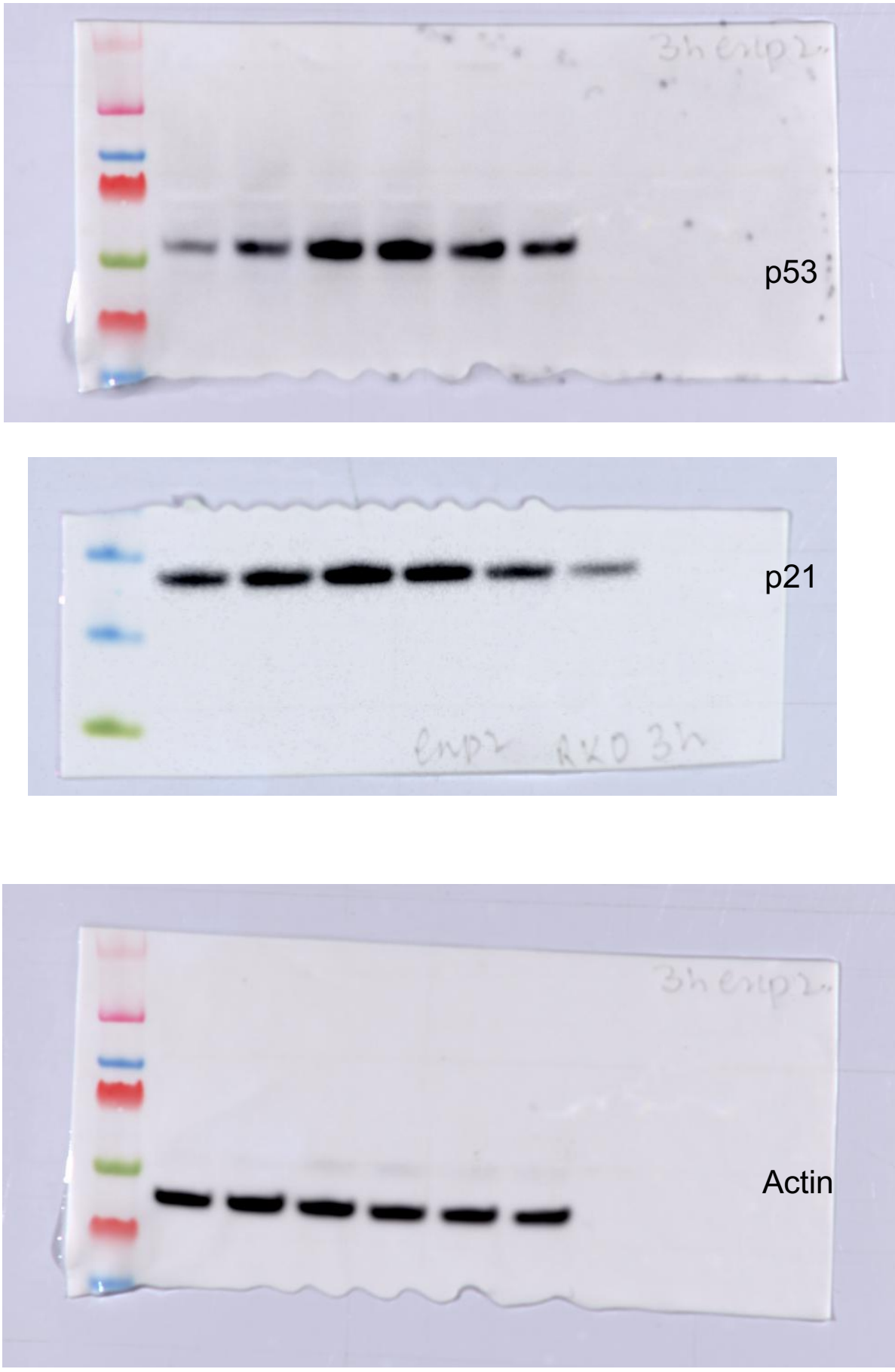

RKO 24h

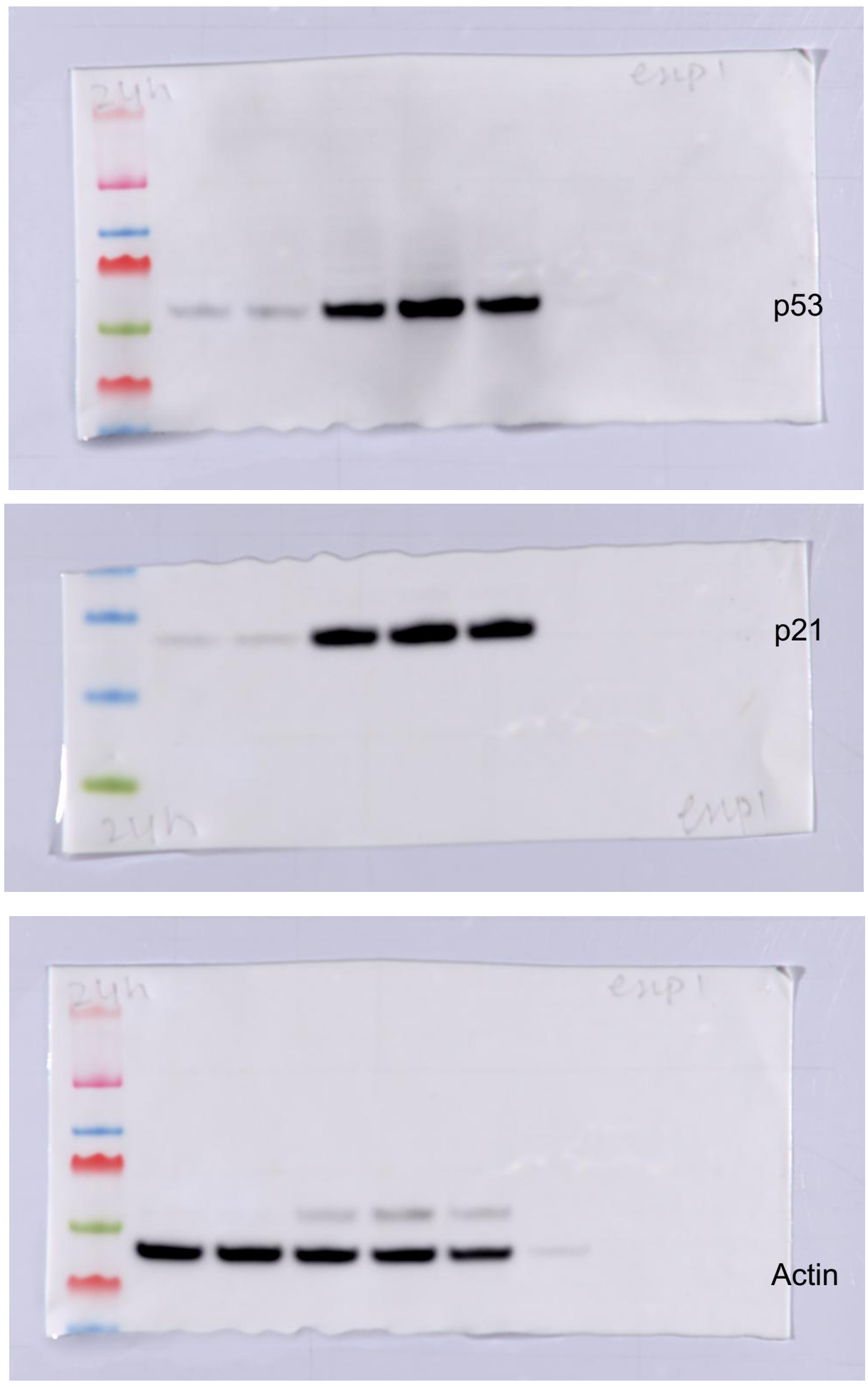

A172 3h

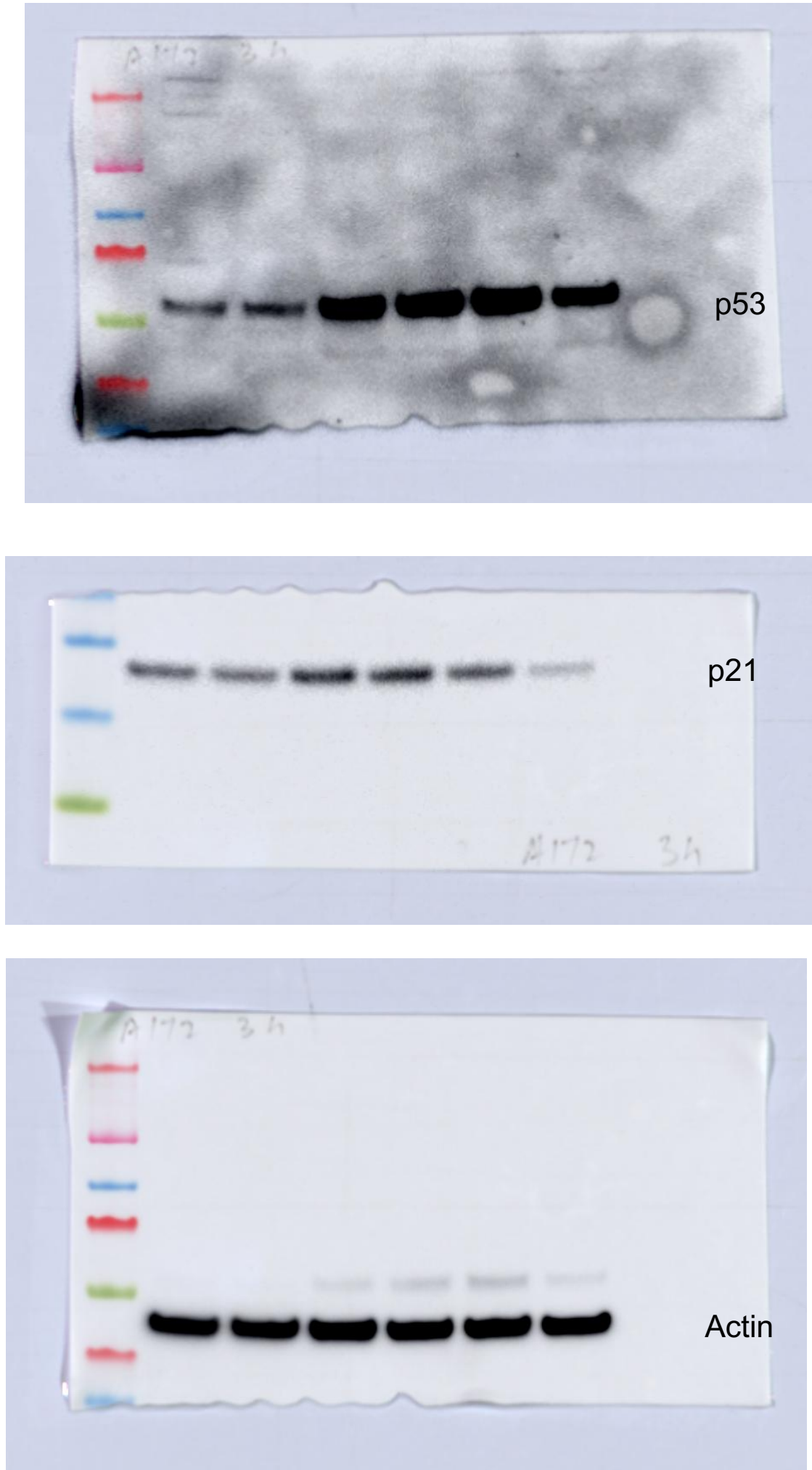

A172 24h

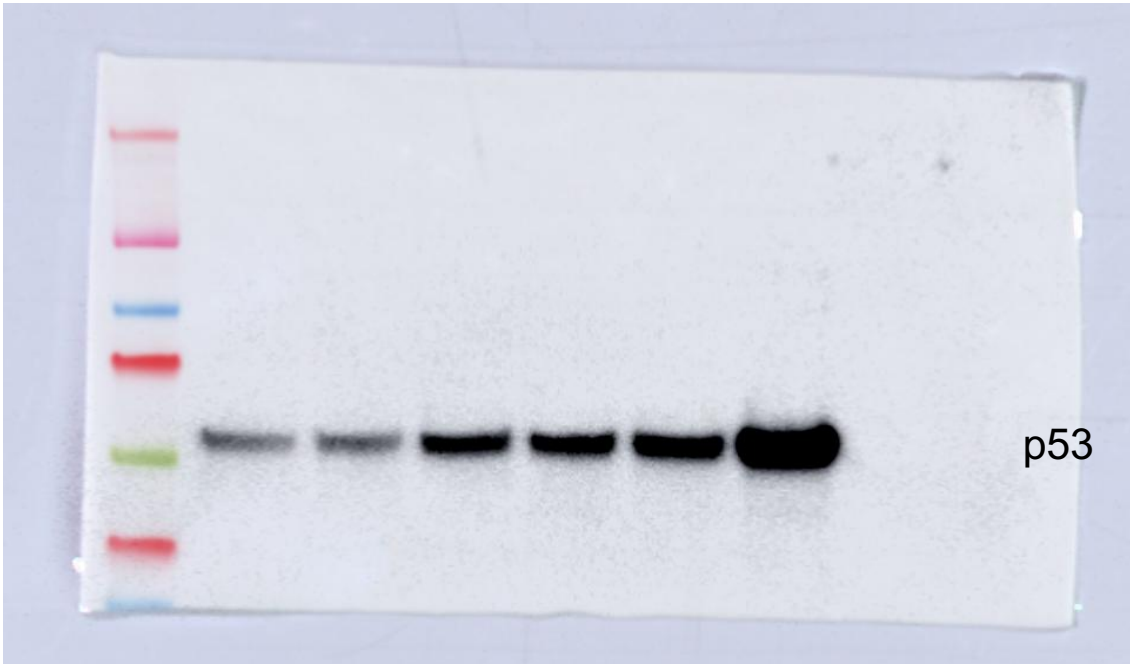

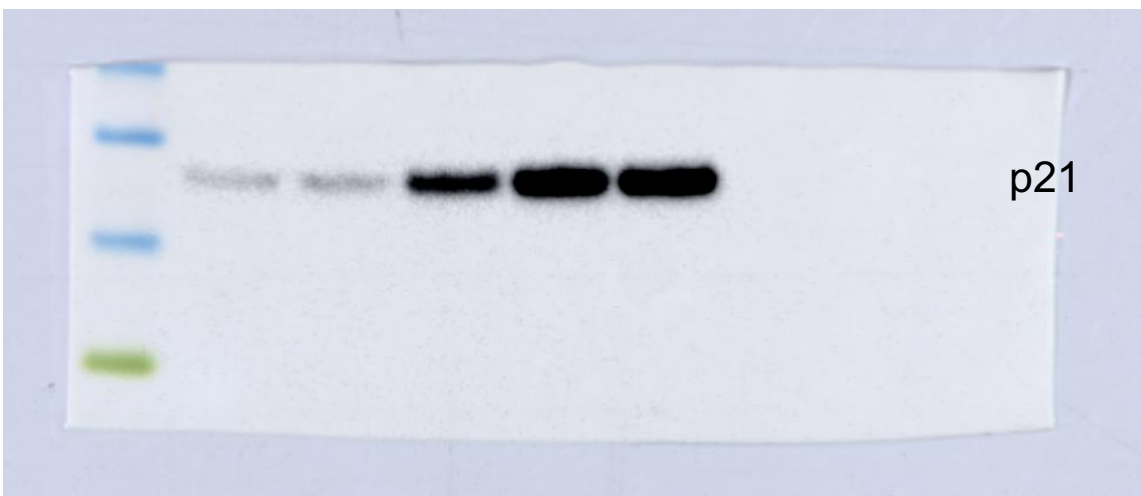

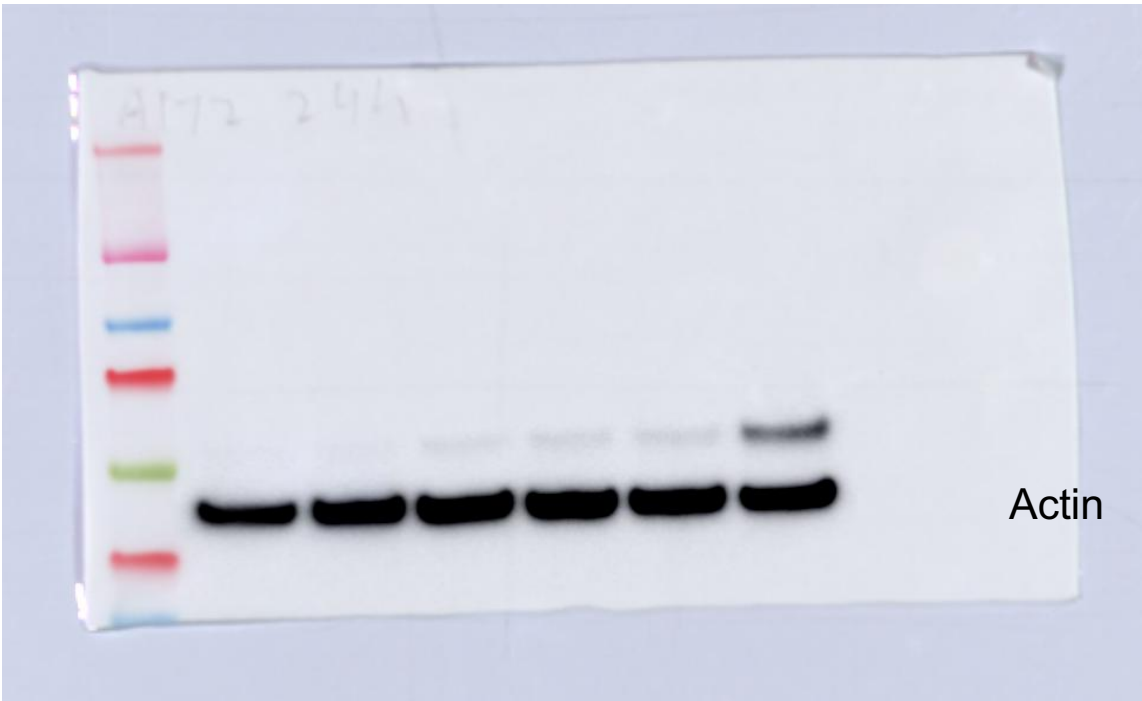

Figure 4C

HCT116 WT p53

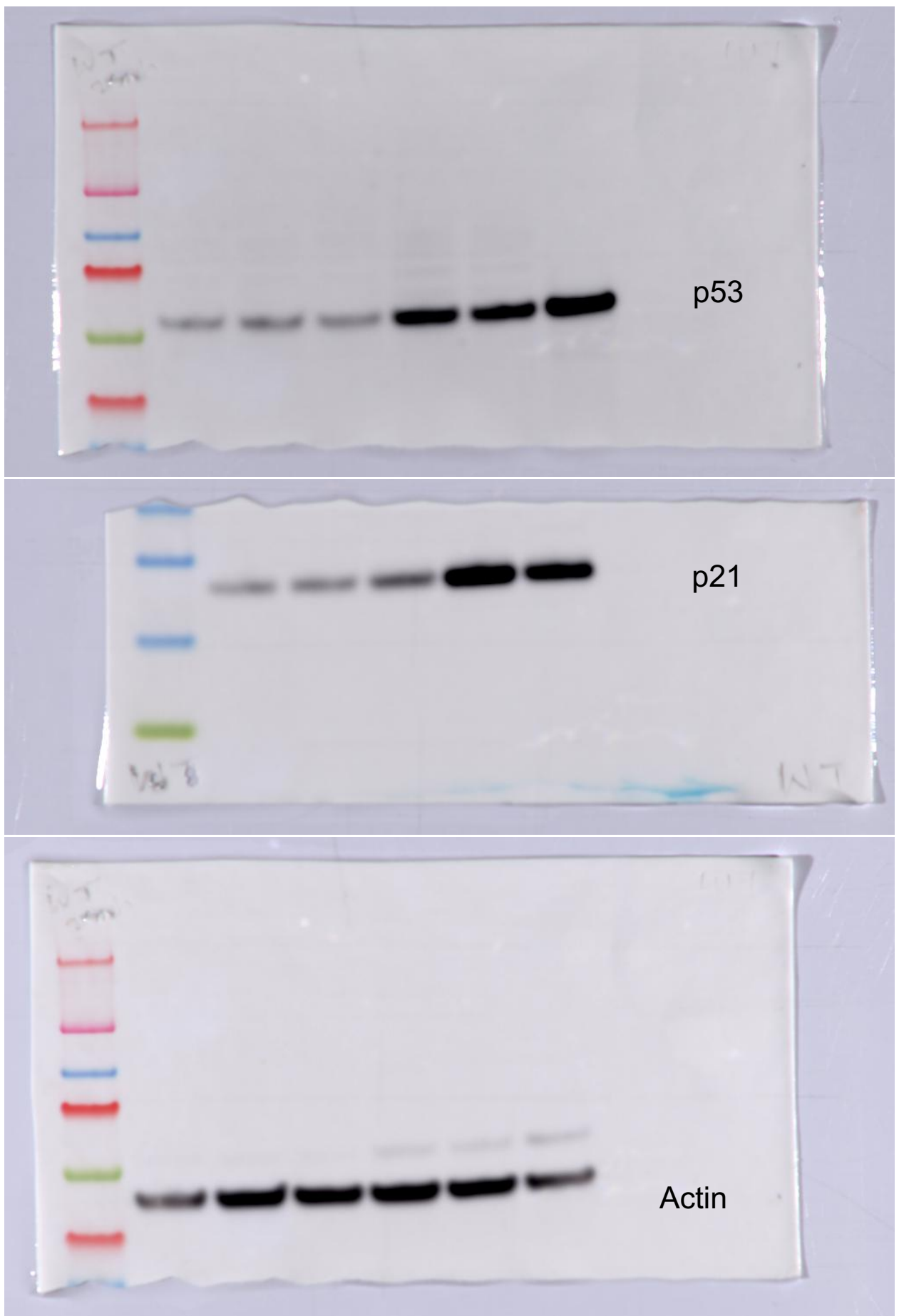

HCT116 p53 KO

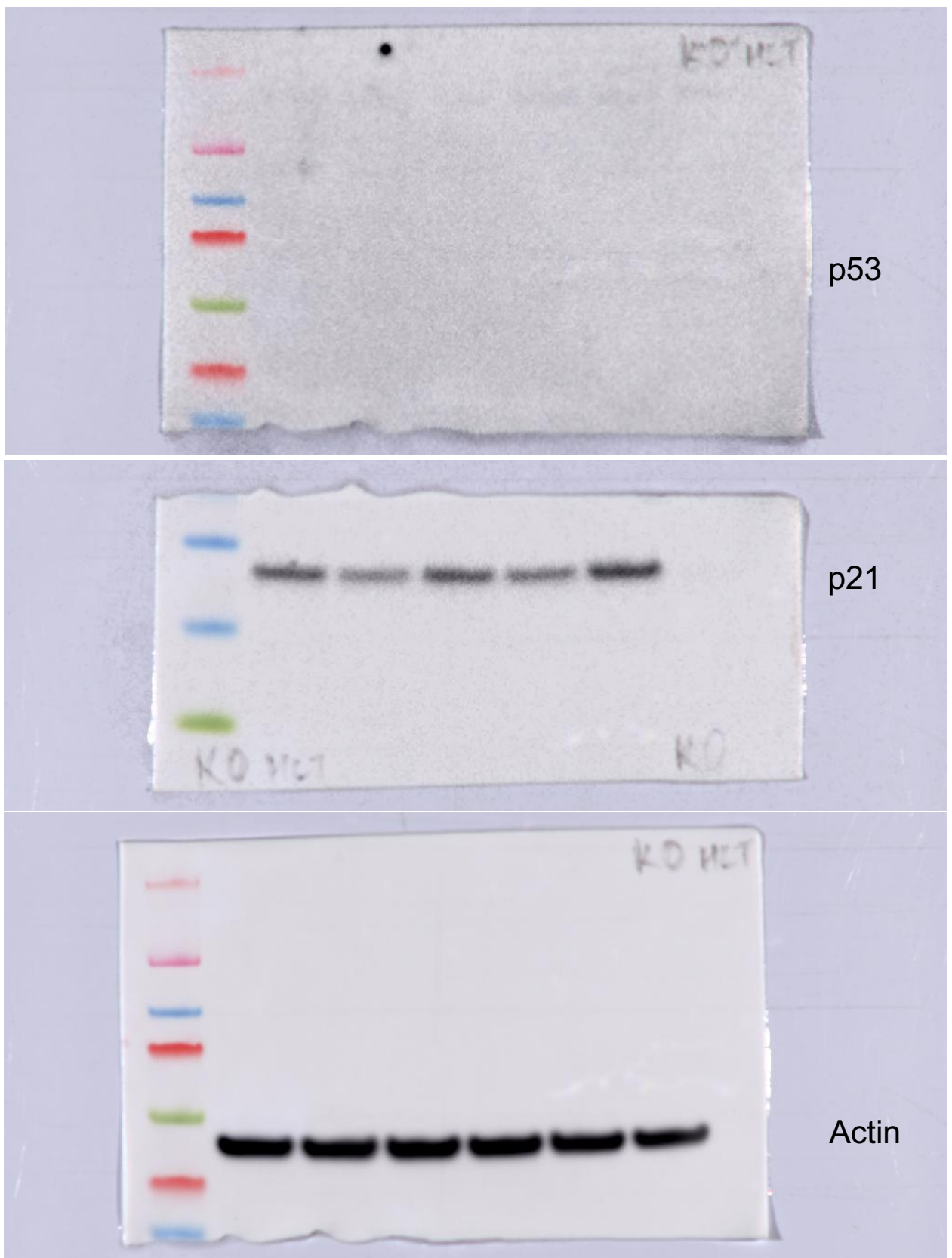

Figure 4D

ZMAT3 – 3h

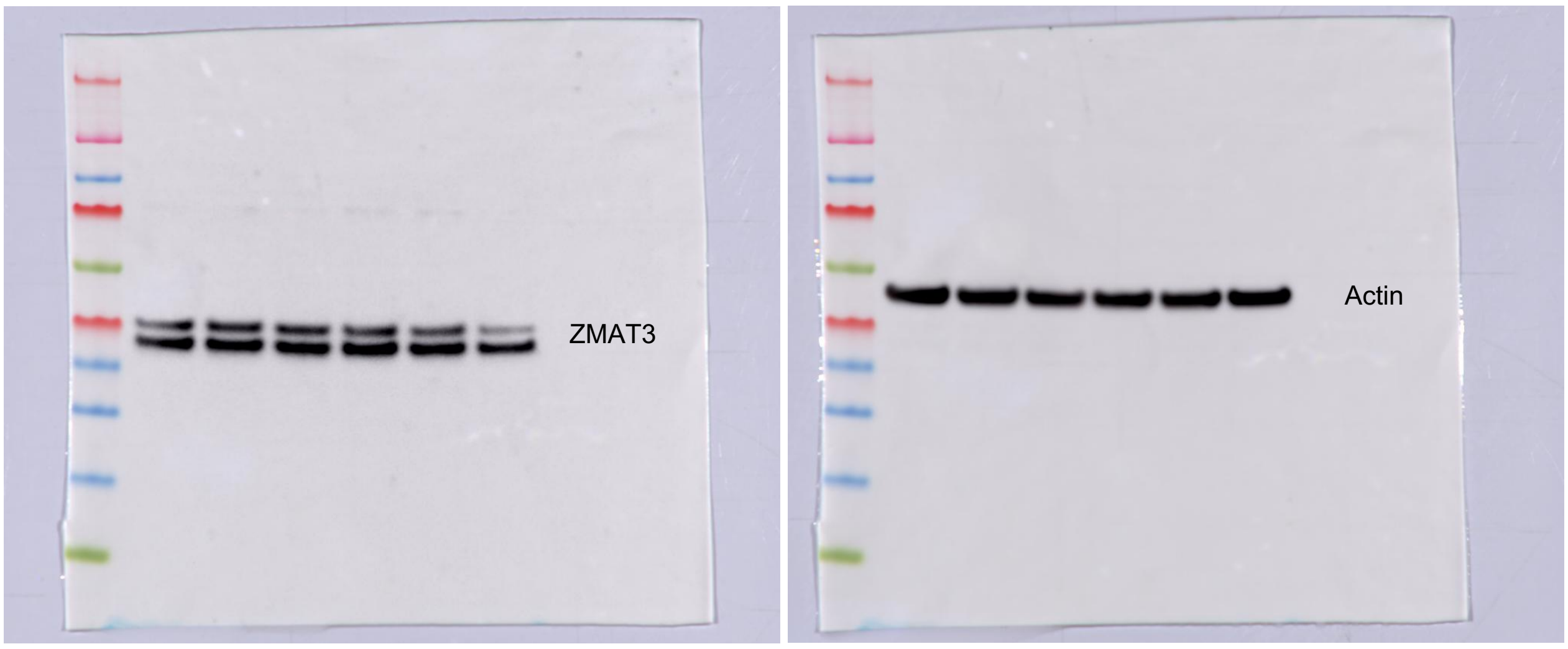

ZMAT3 – 24h

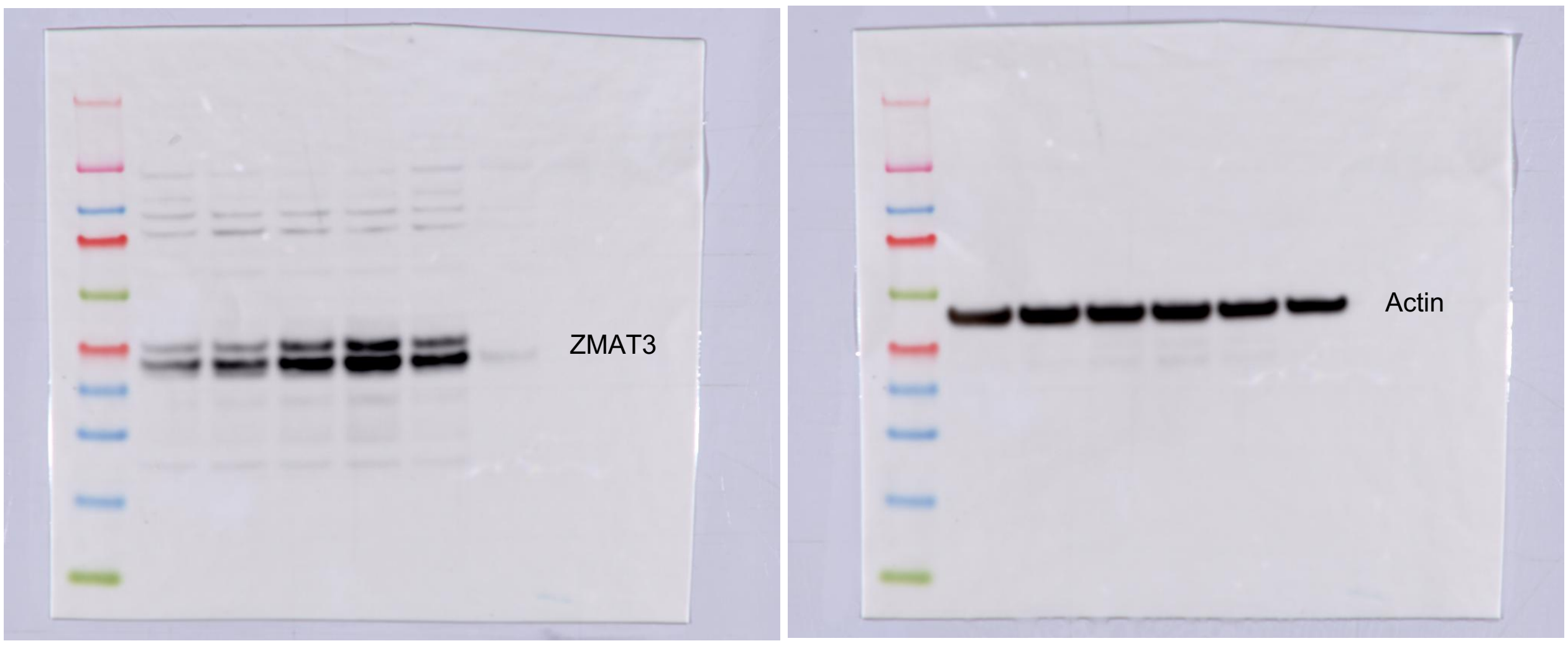

MDM2 – 3h

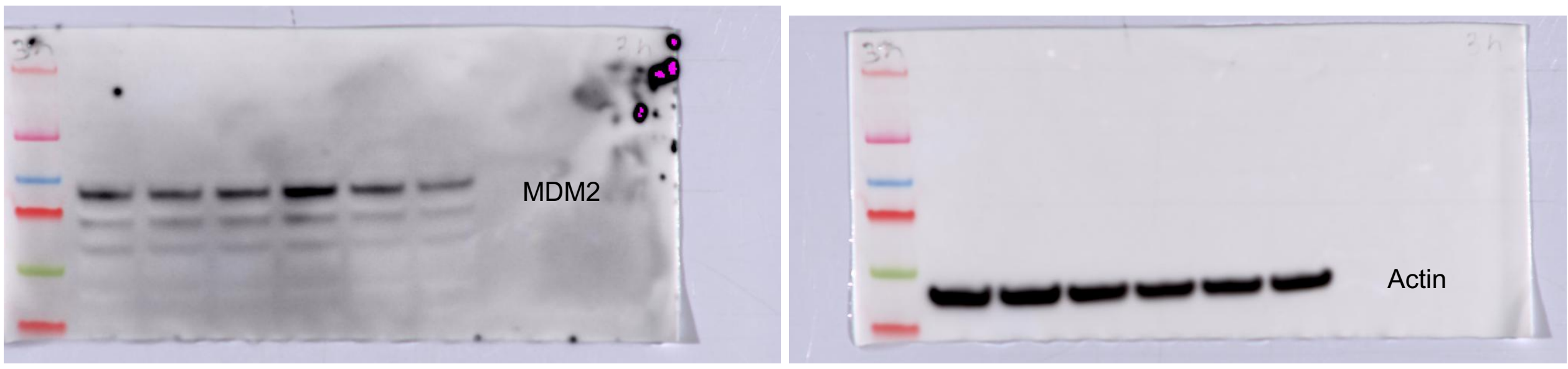

MDM2 – 24h

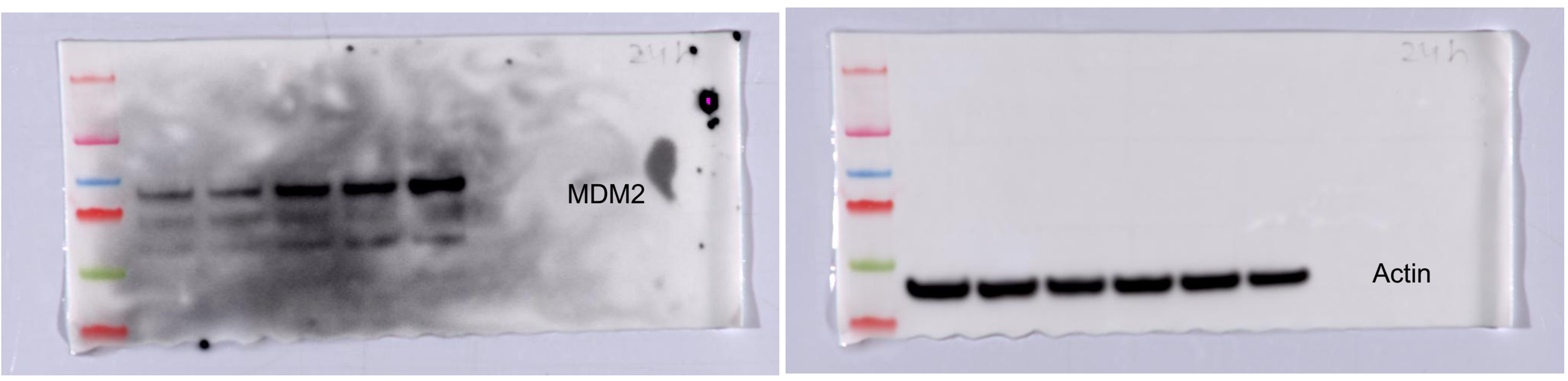

MYC – 3h

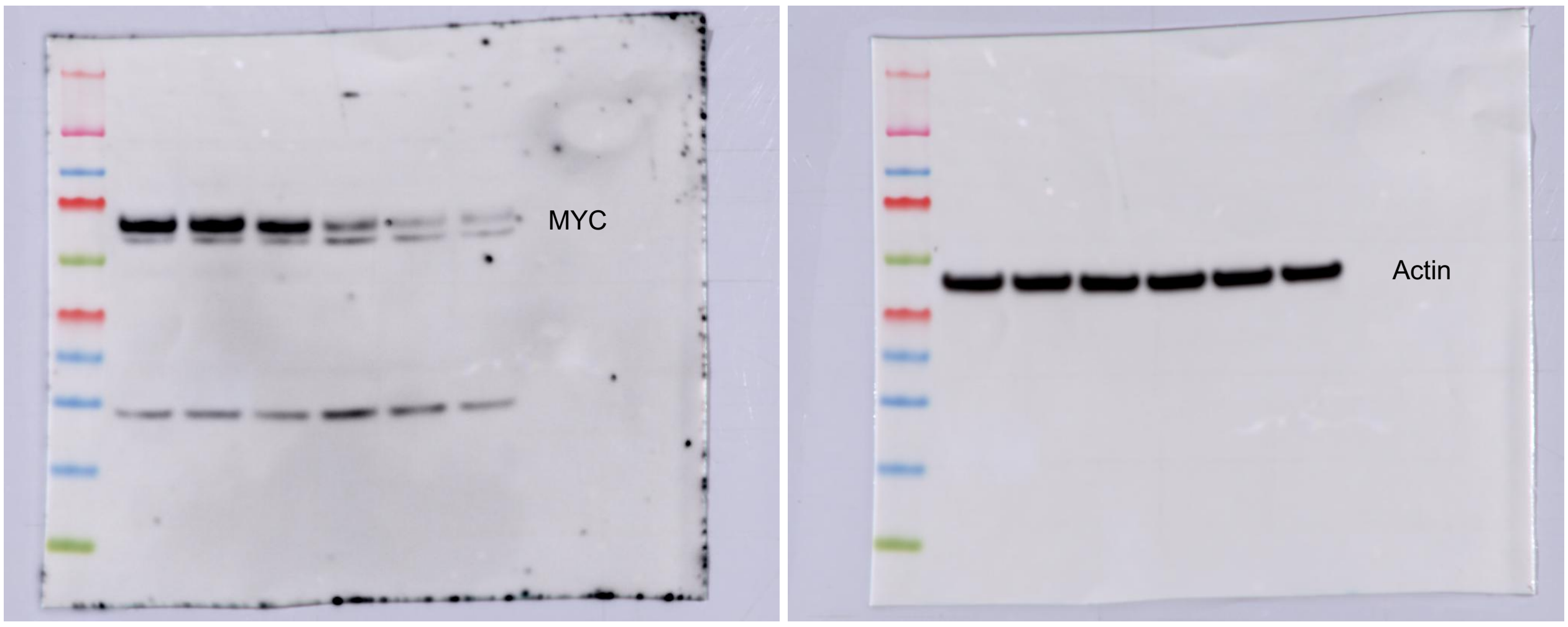

MYC – 24h

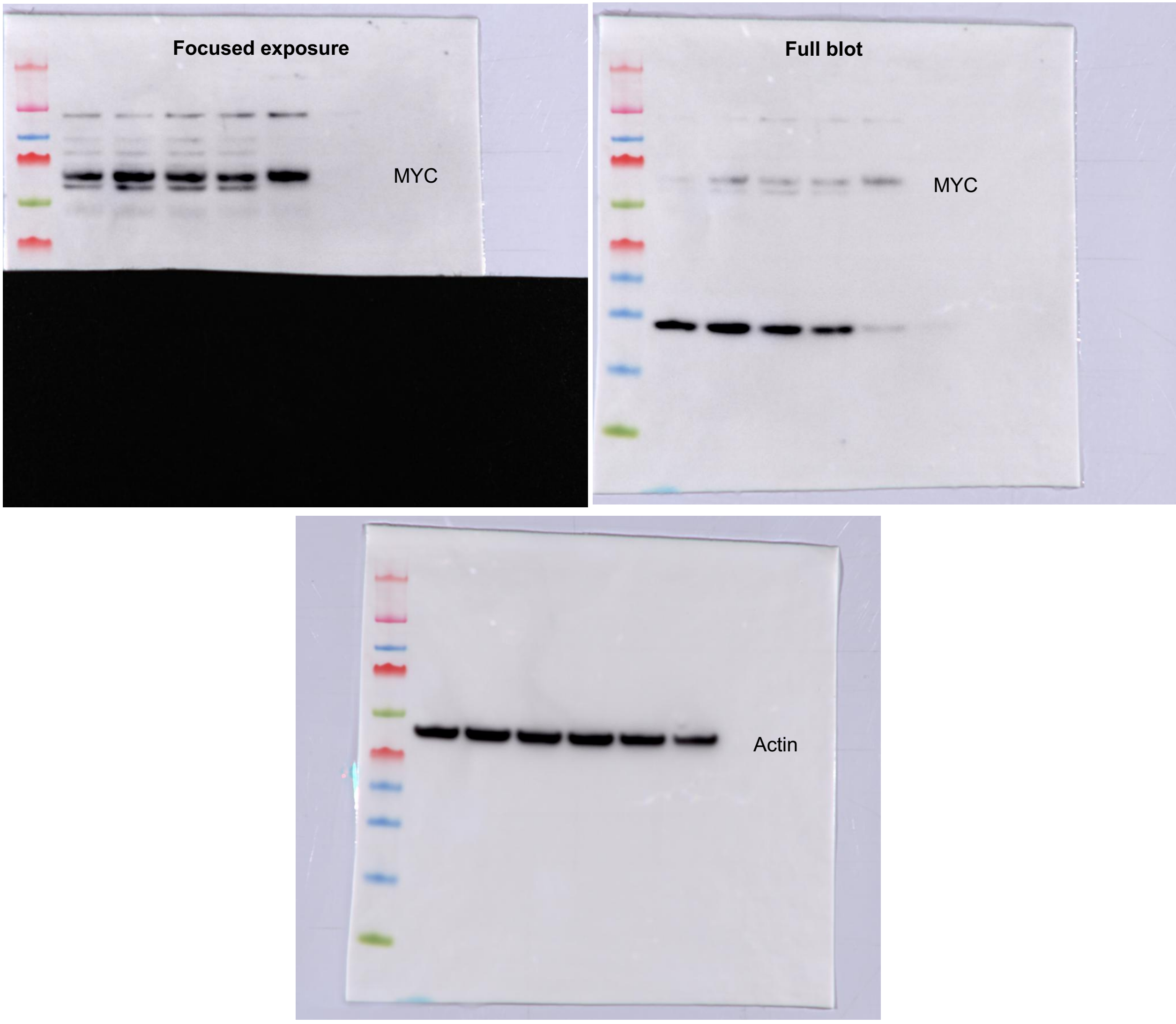

Figure 4E

RPL5 and RPL11 – 3h

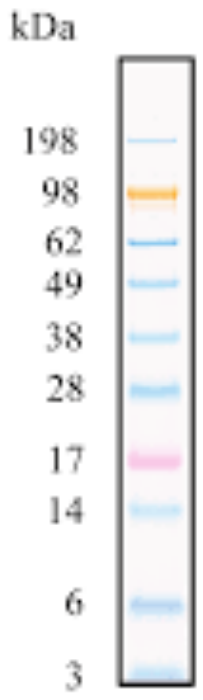

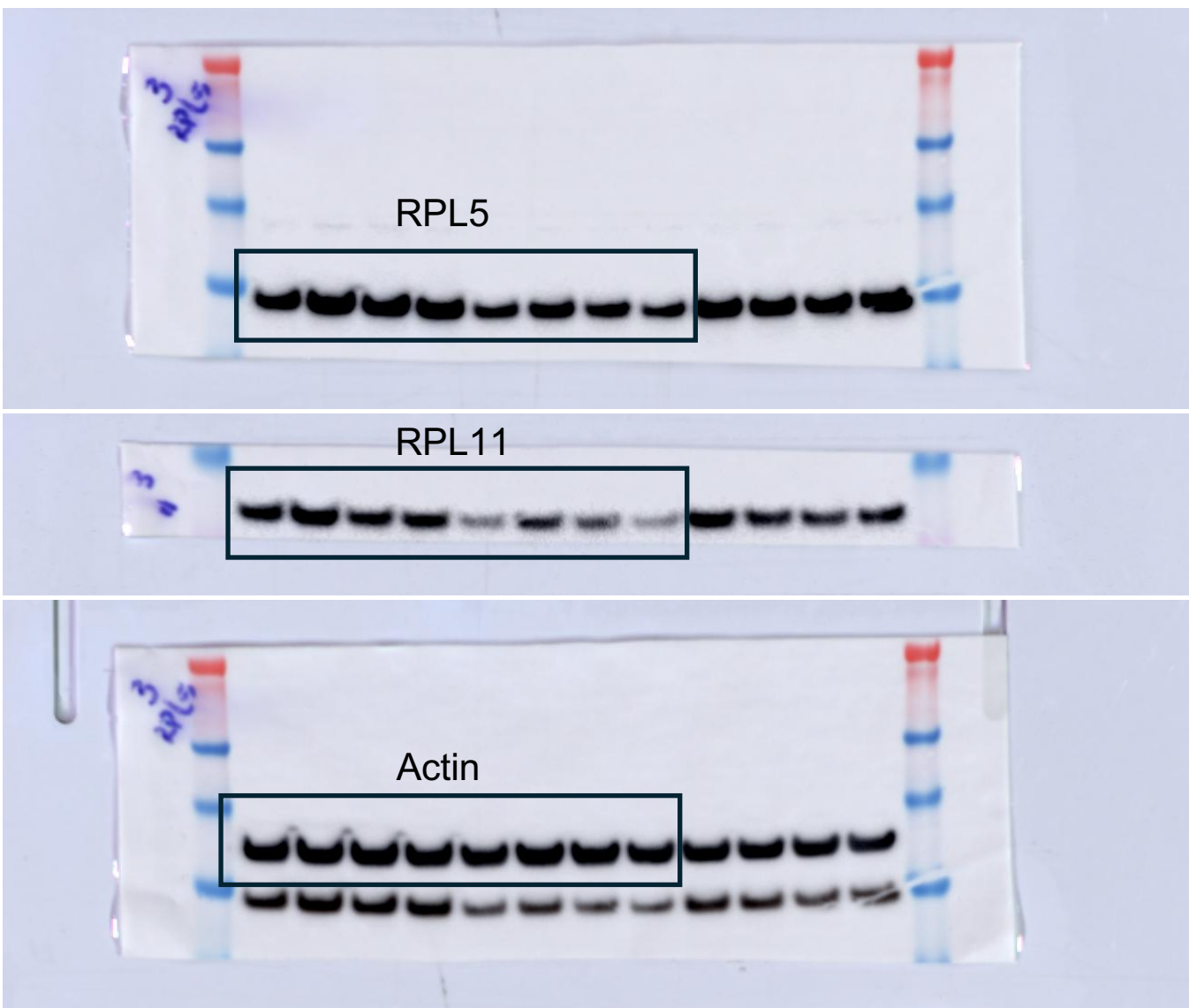

RPL5 and RPL11 – 24h

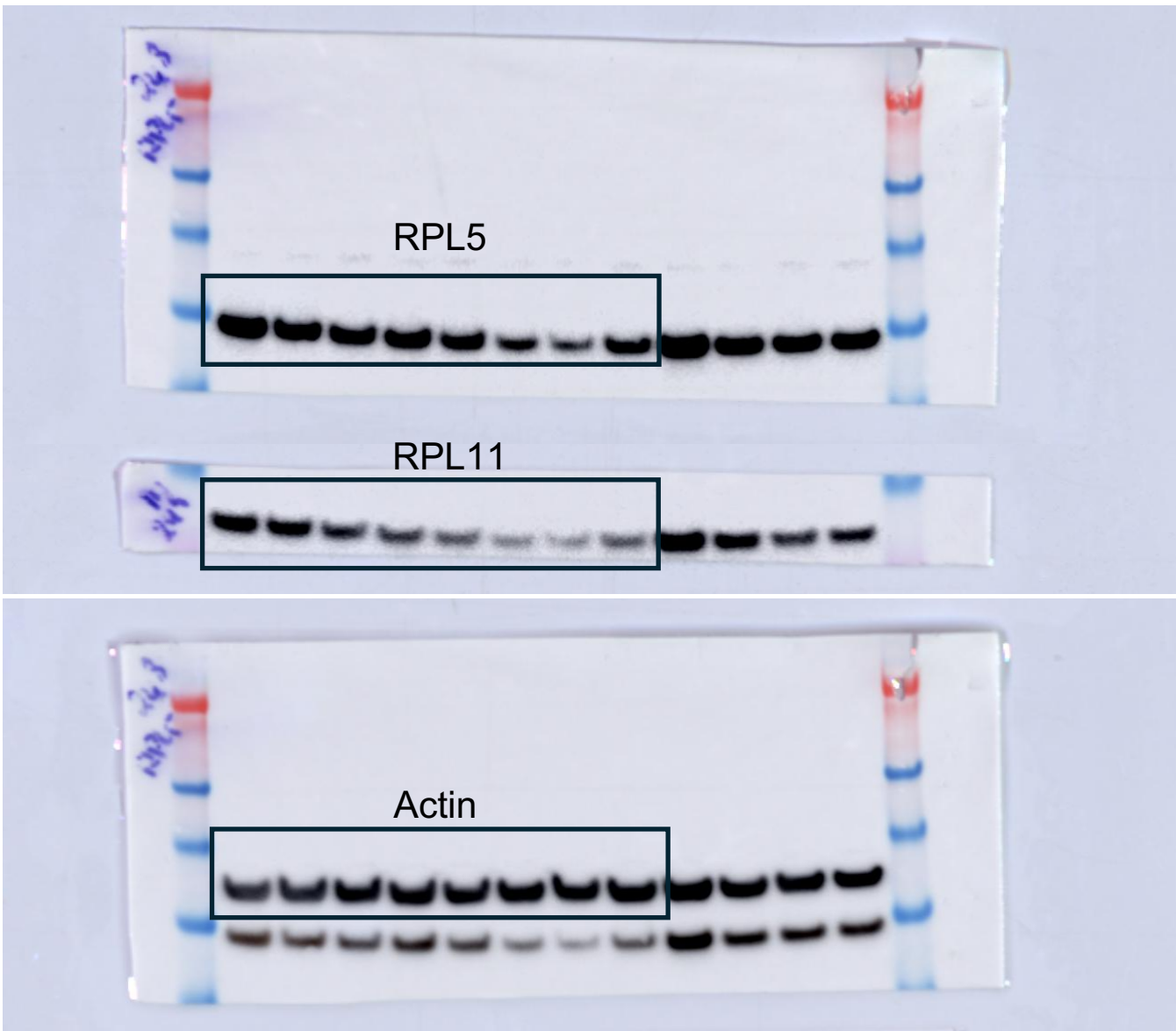

p53 and p21 – 3h

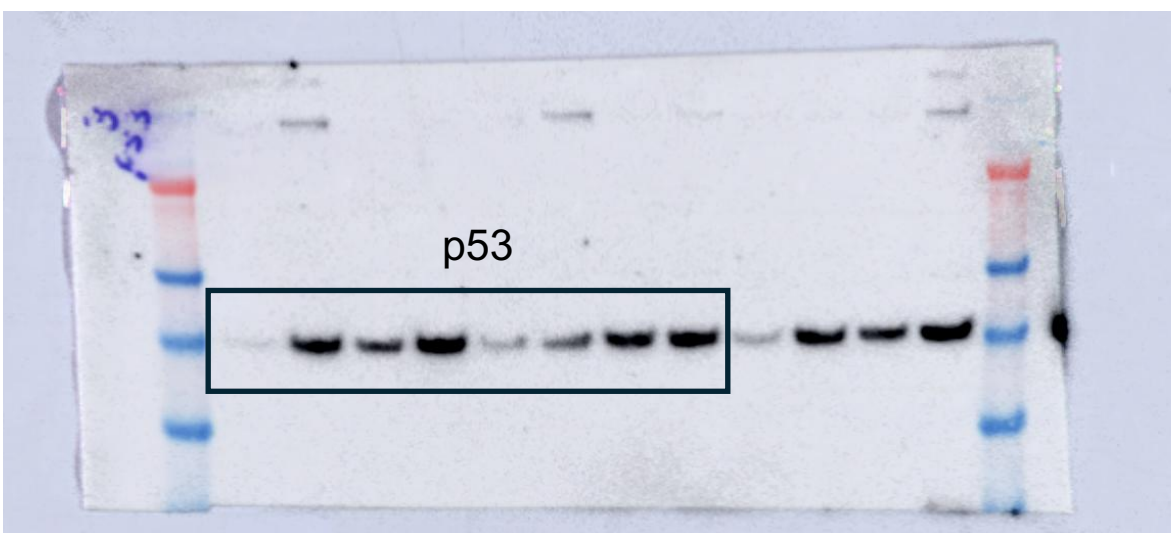

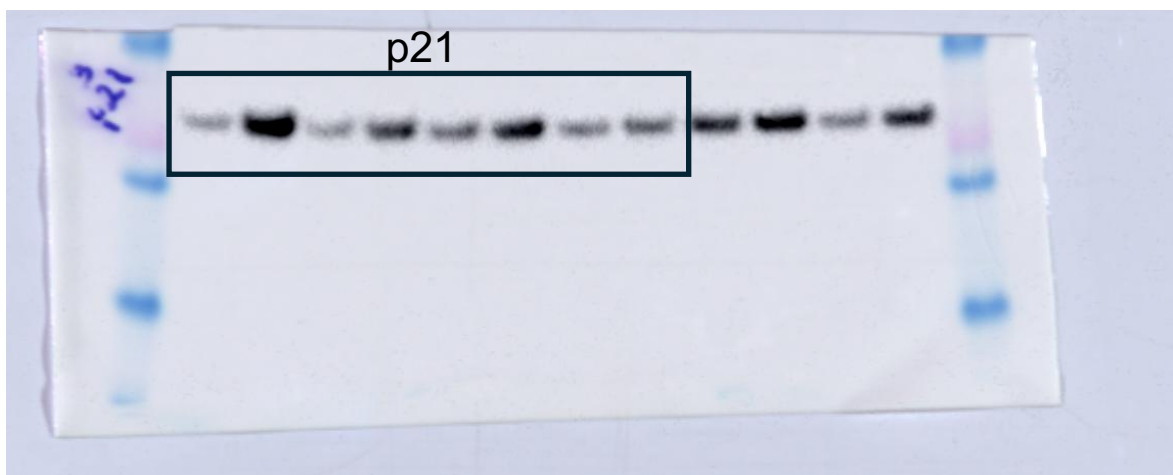

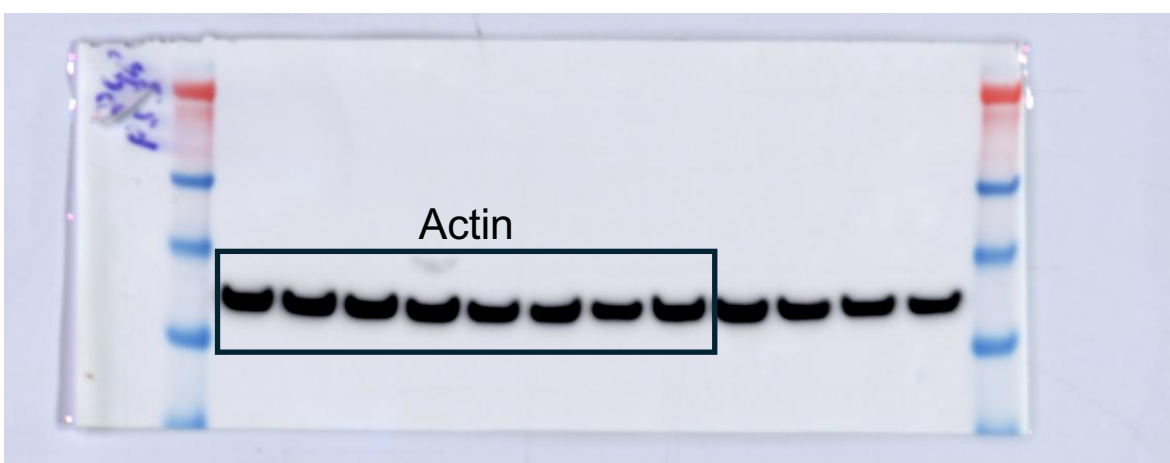

p53 and p21 – 24h

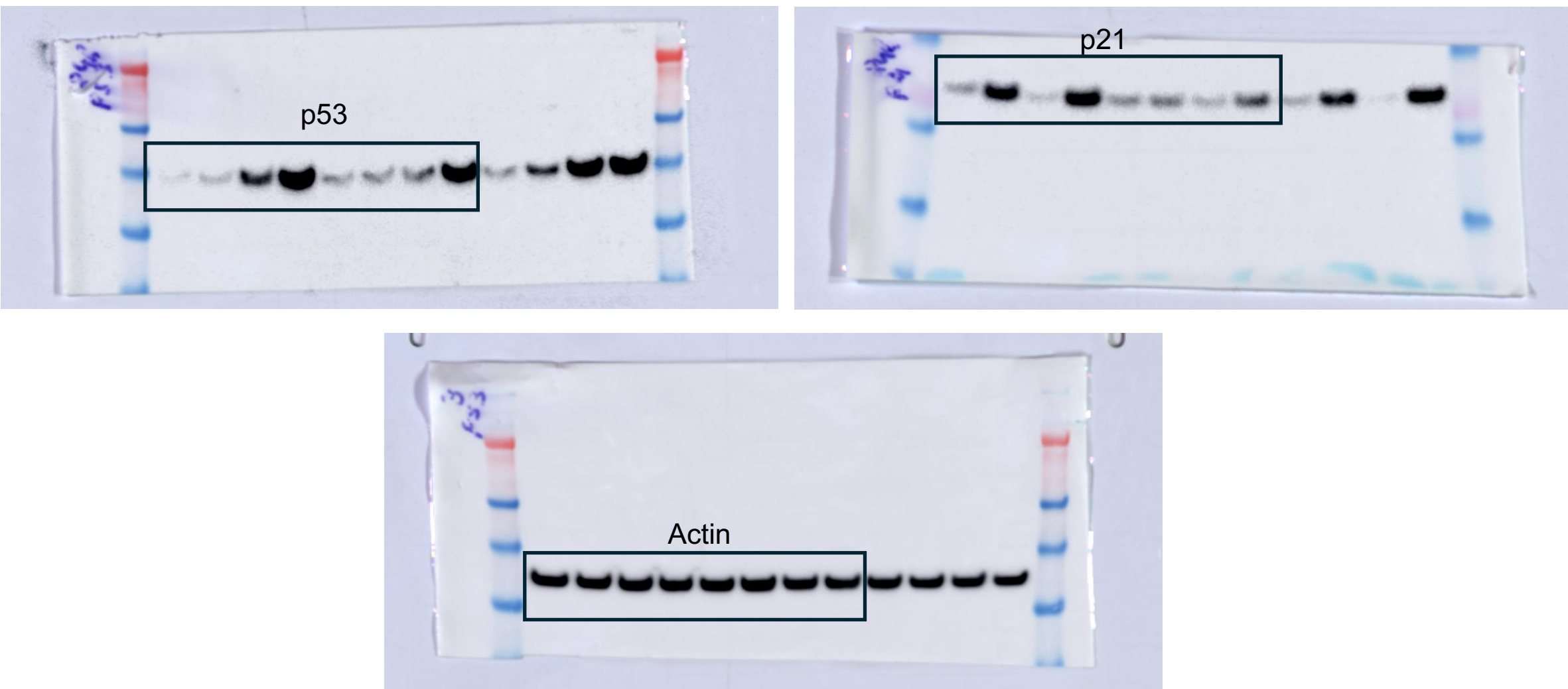

Figure 6A

Phospho H2A.X 3h

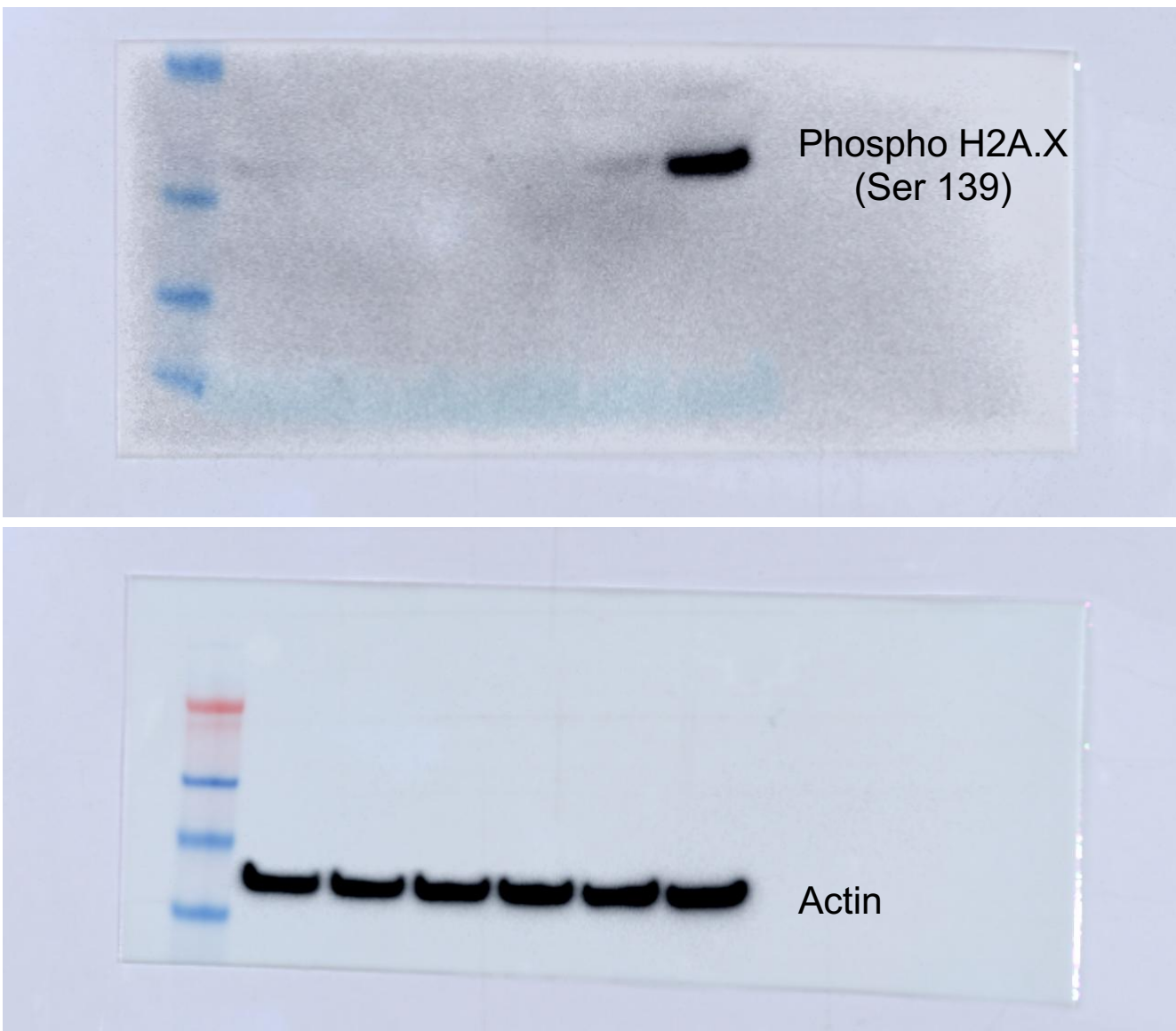

Phospho H2A.X 24h

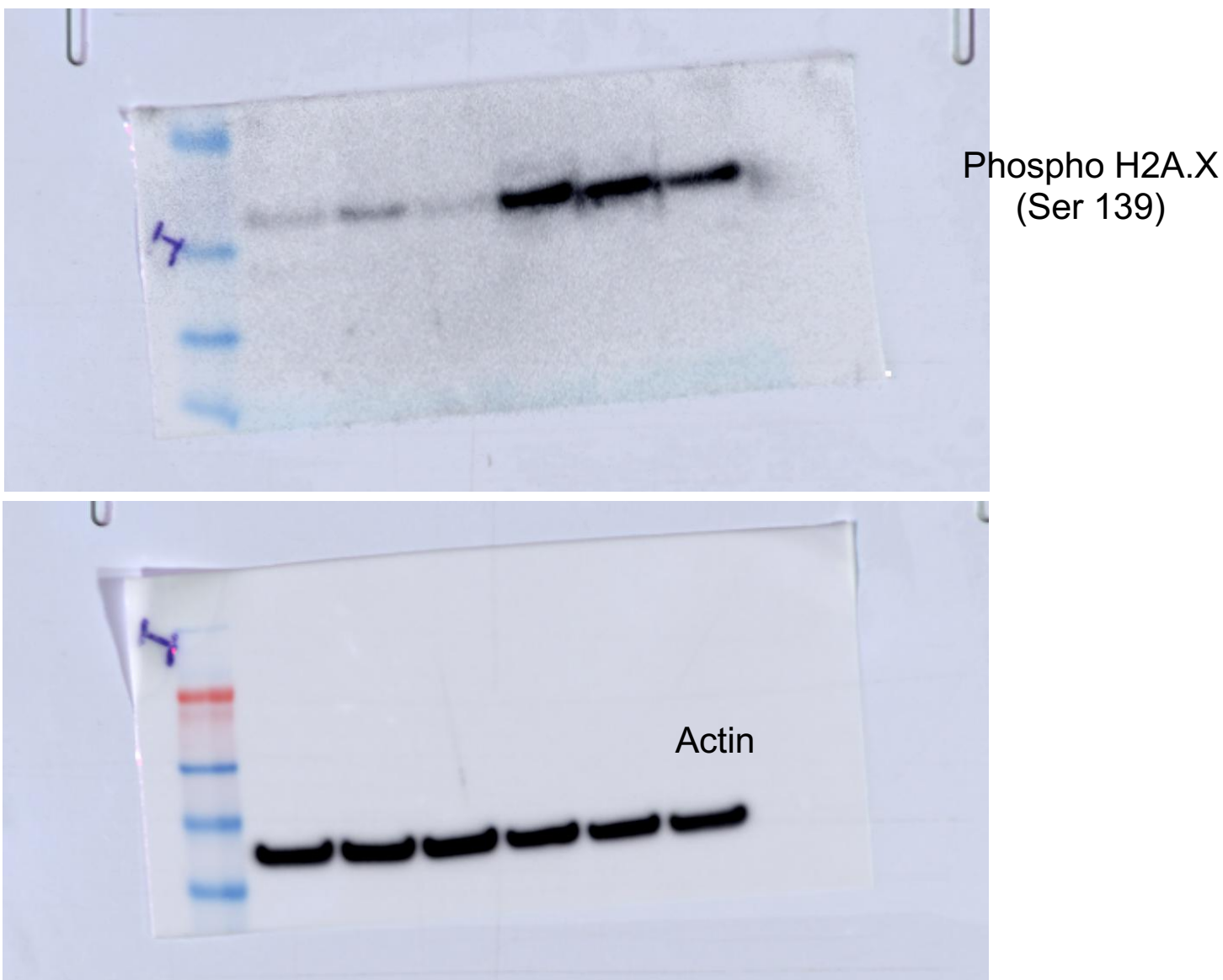

Figure 6C
RADAR assay

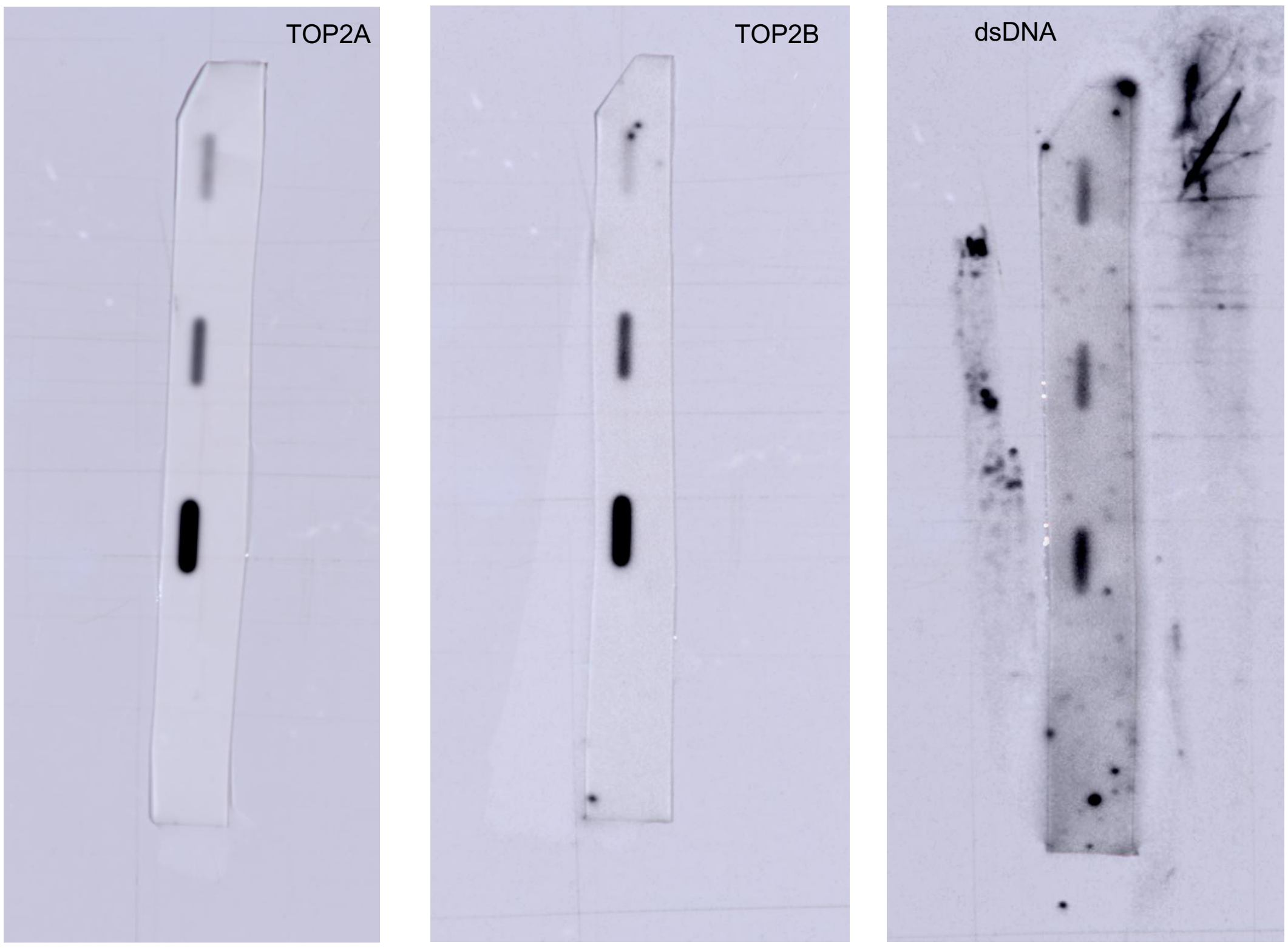

Figure 6D
Subcellular Fractionation

TOP2A – Whole cell lysate

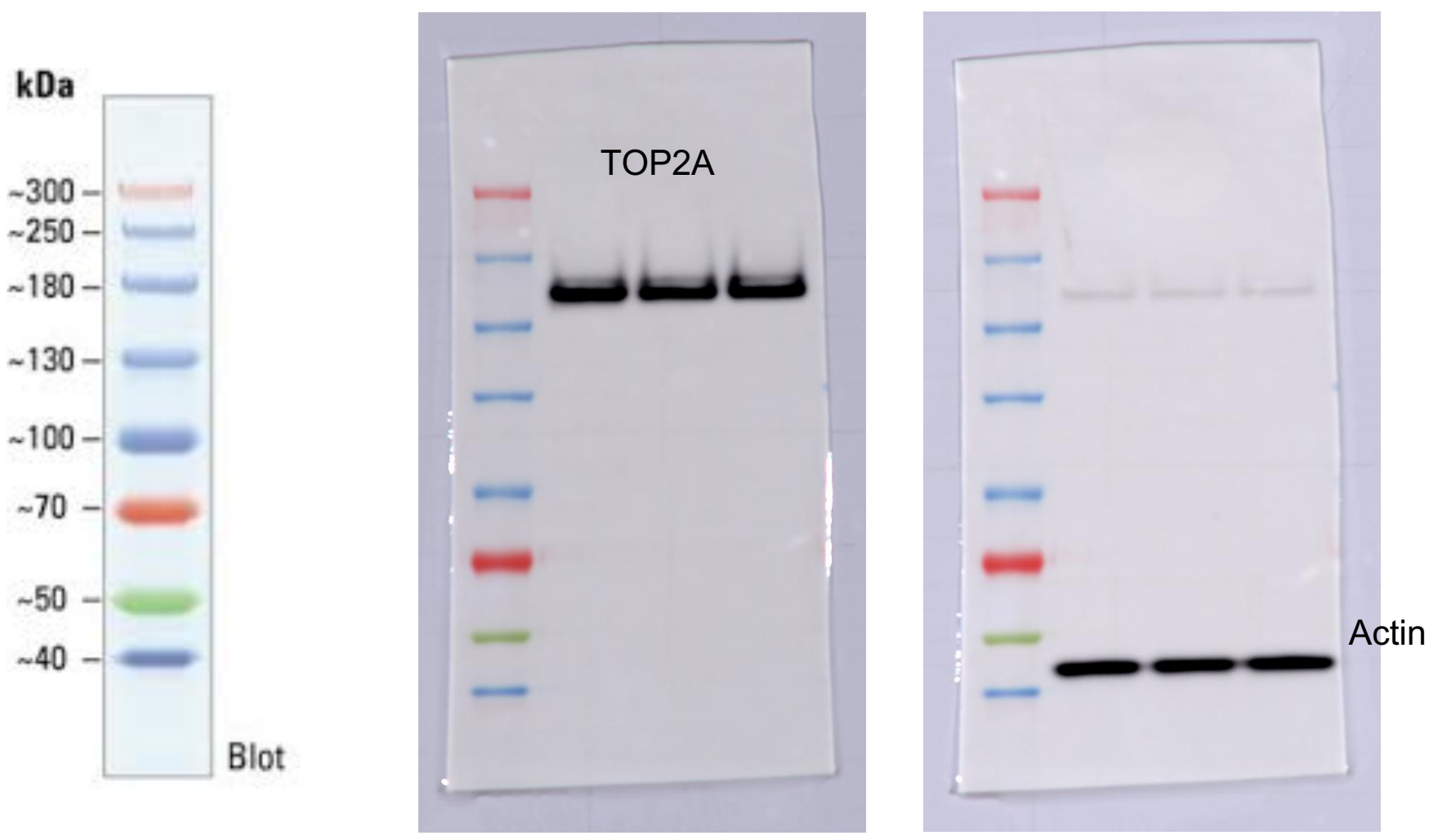

TOP2A – Chromatin fraction

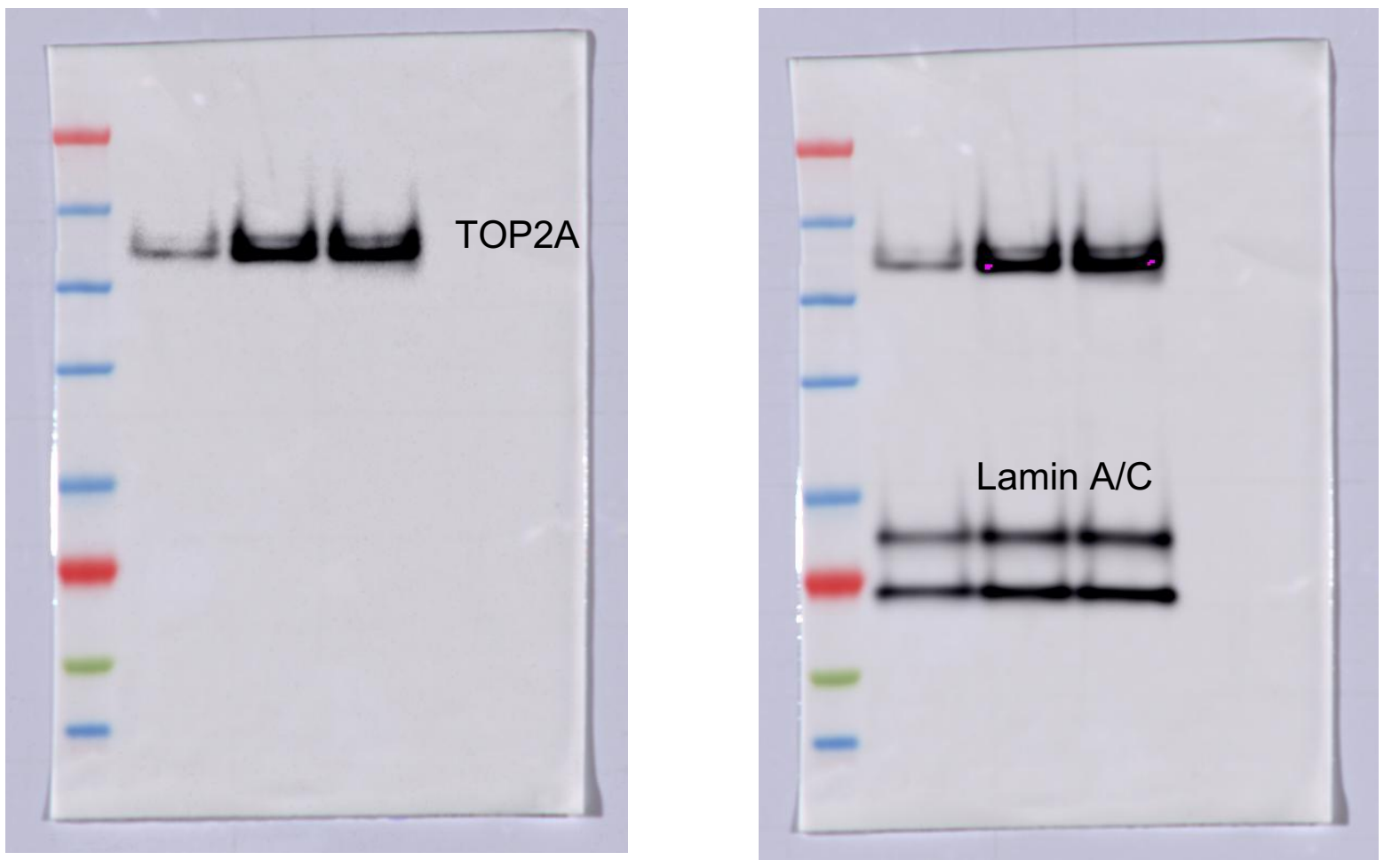

TOP2B – Whole cell lysate

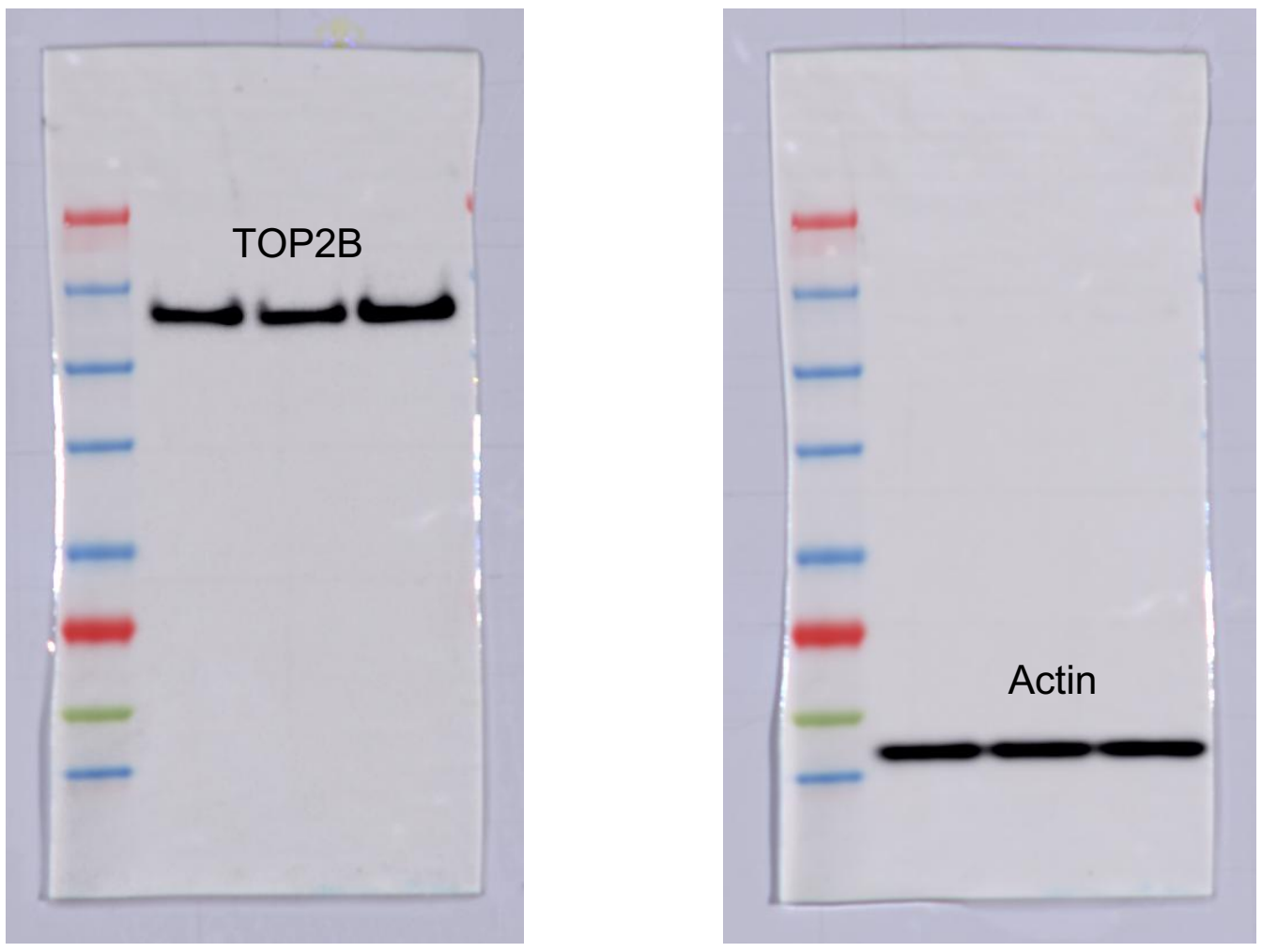

TOP2B – Chromatin fraction

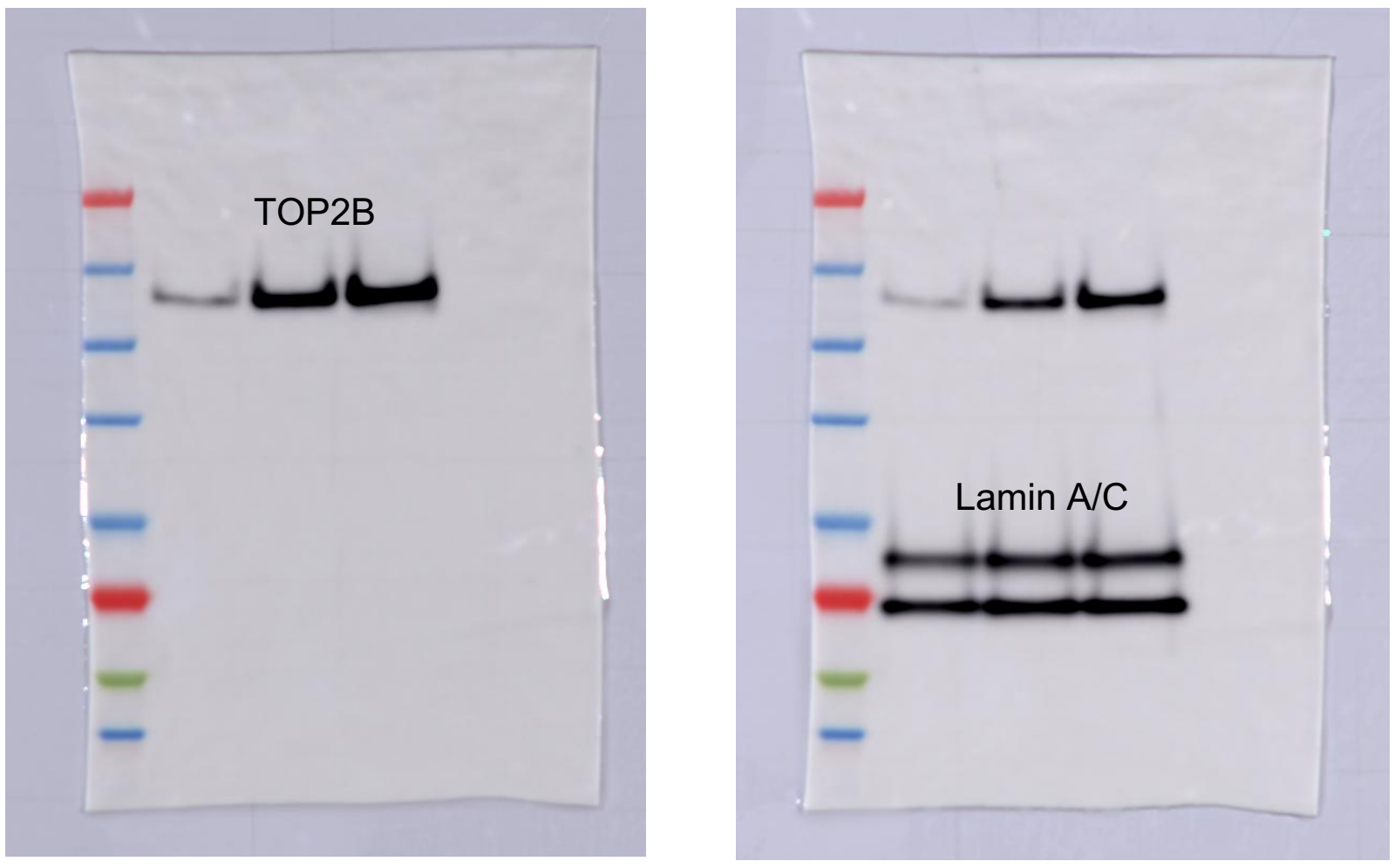

SSRP1 and SPT16

Whole cell lysate

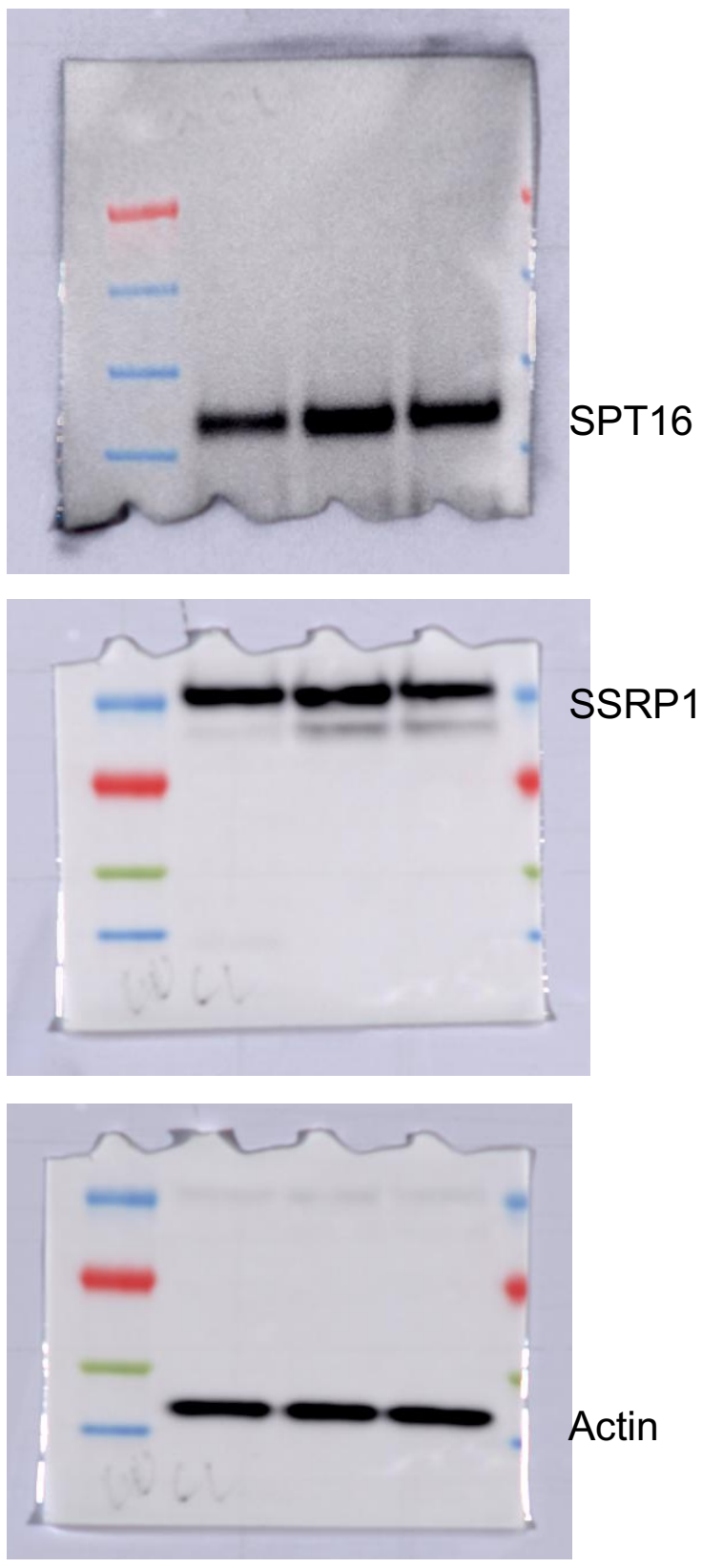

Chromatin fraction

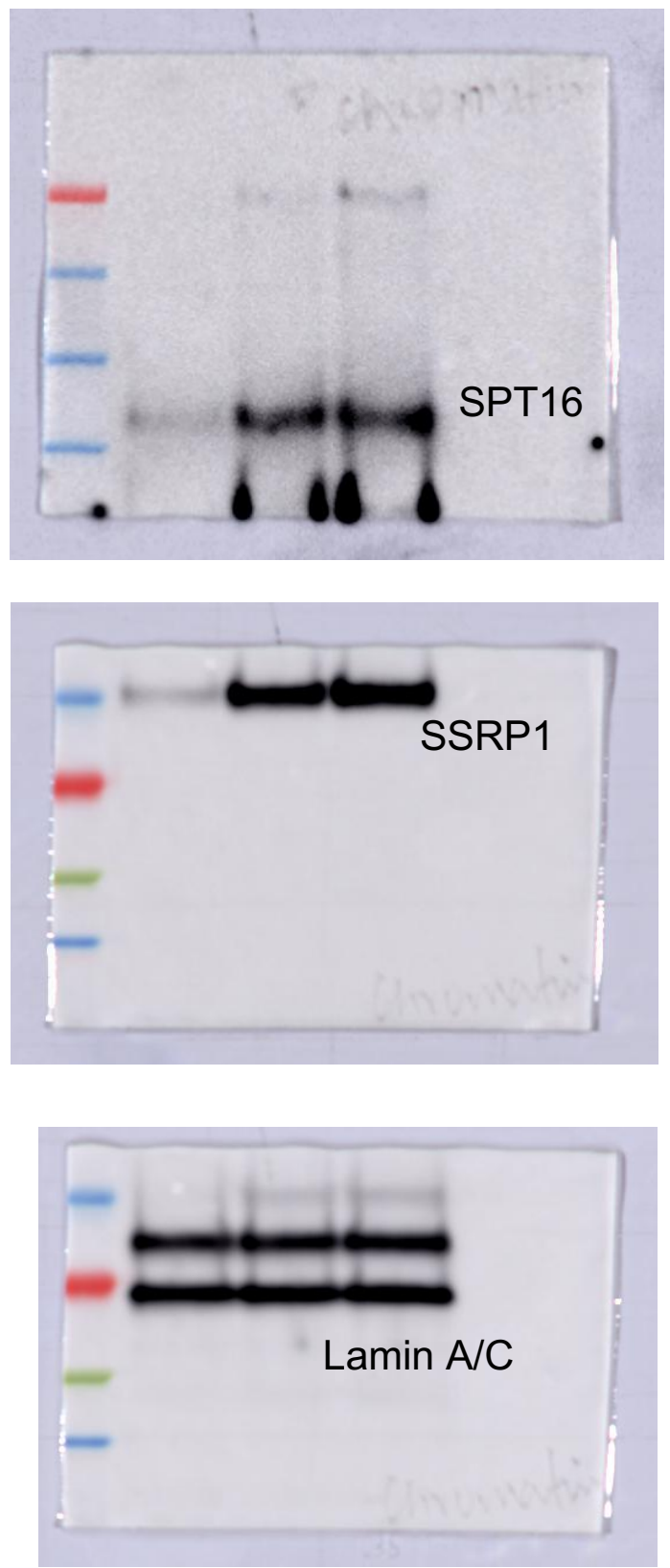

TOP1

Whole cell lysate

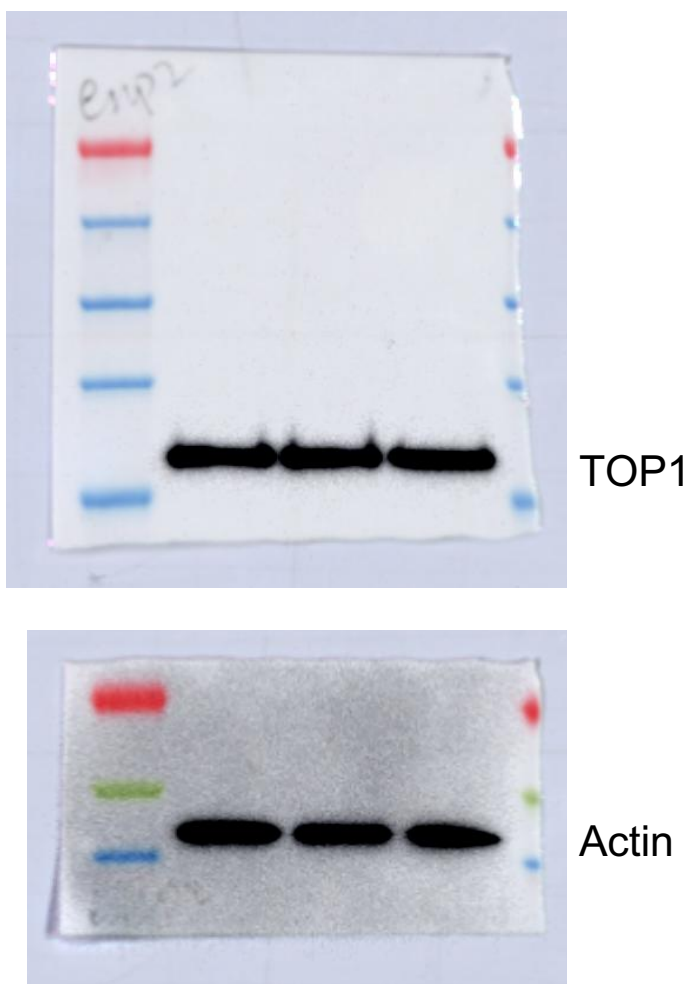

Chromatin fraction

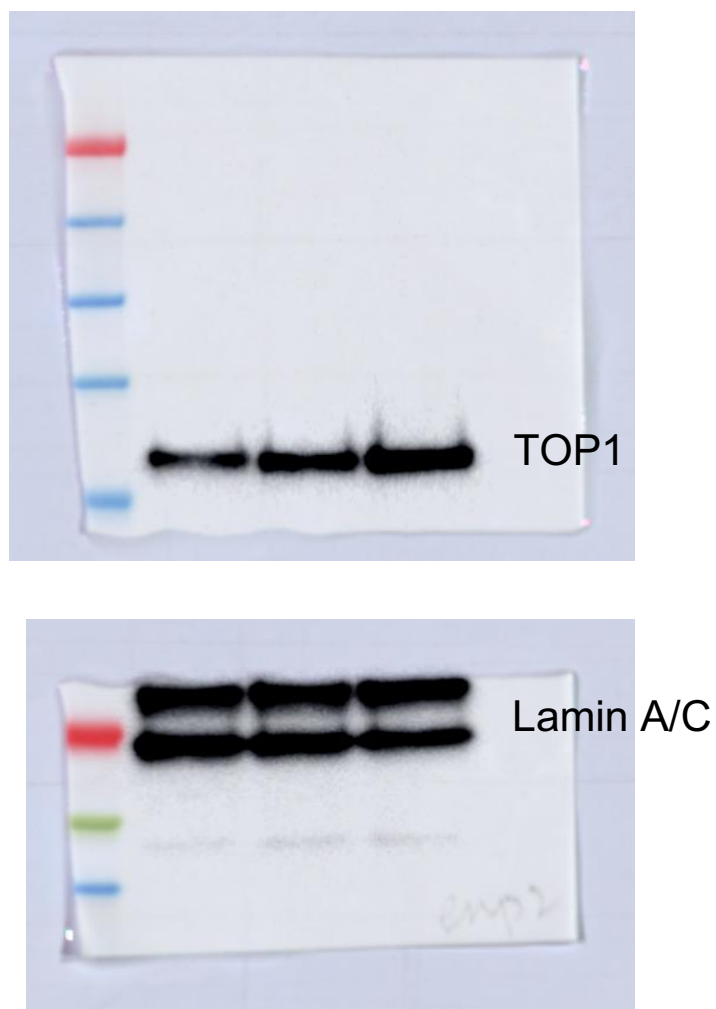

Supplementary Figure 2

A549 POLR1A 3h

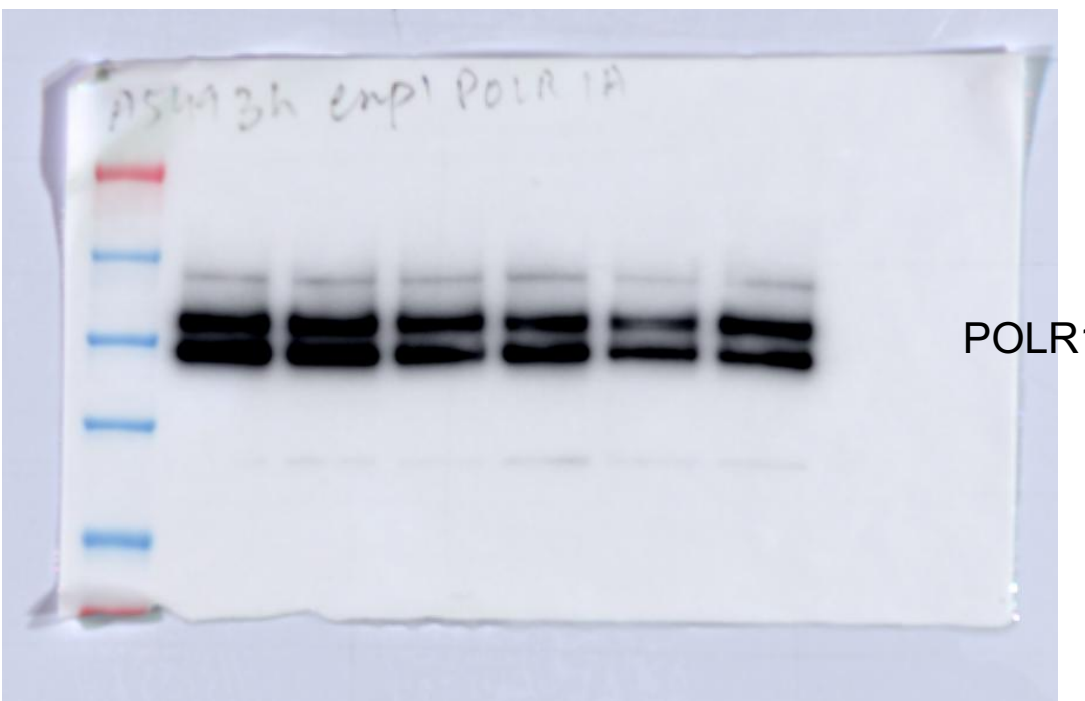

POLR1A

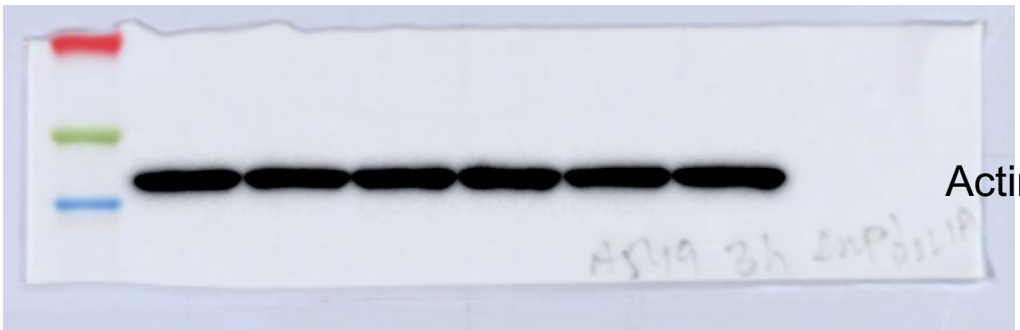

Actin

A549 POLR2A 3h

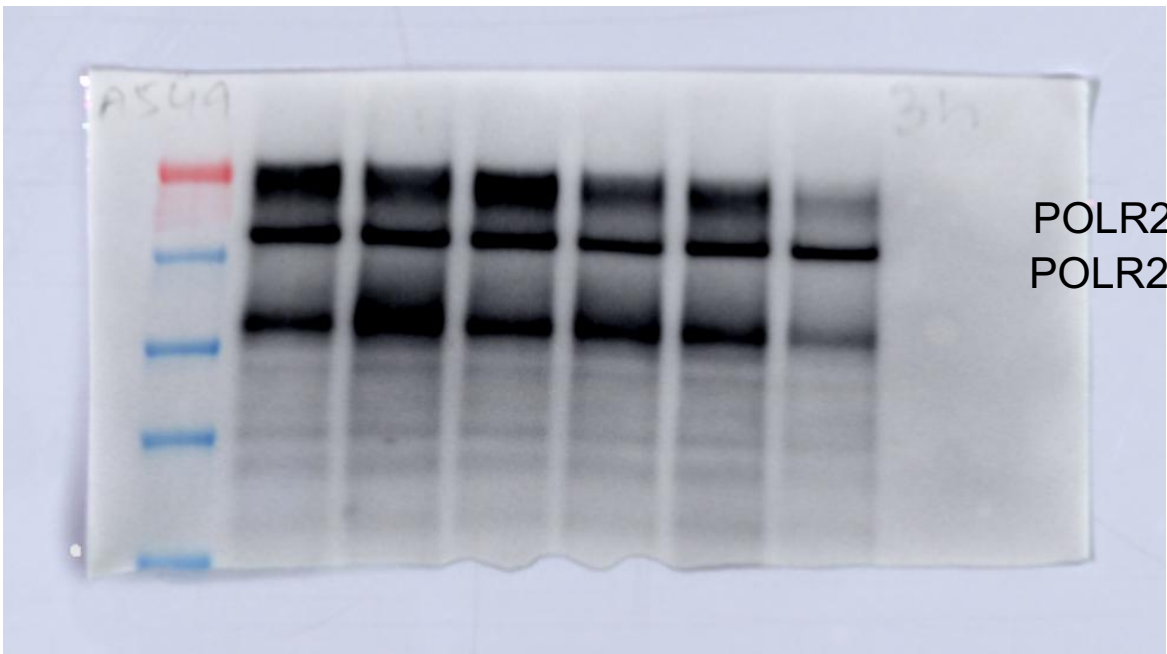

POLR2A IIo
POLR2A IIa

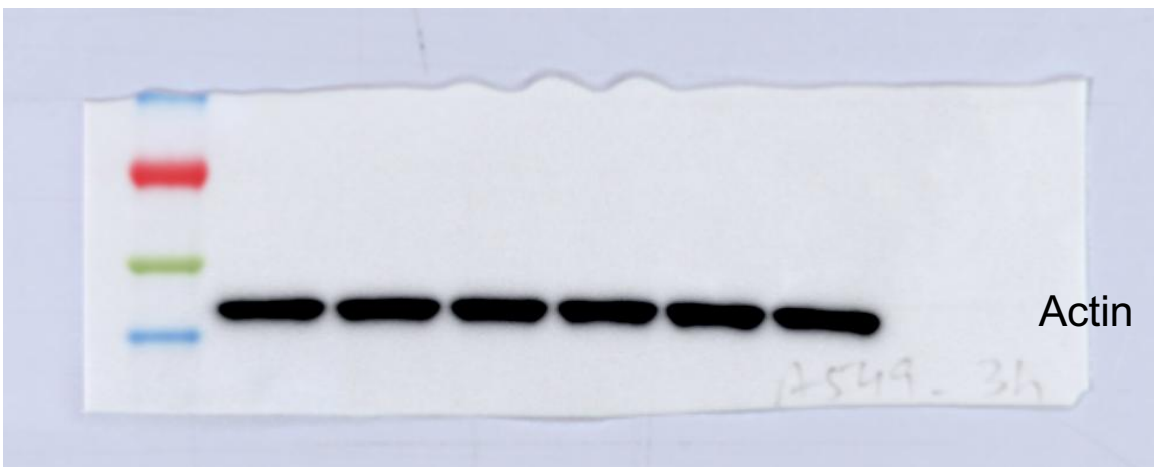

Actin

A549 POLR1A 24h

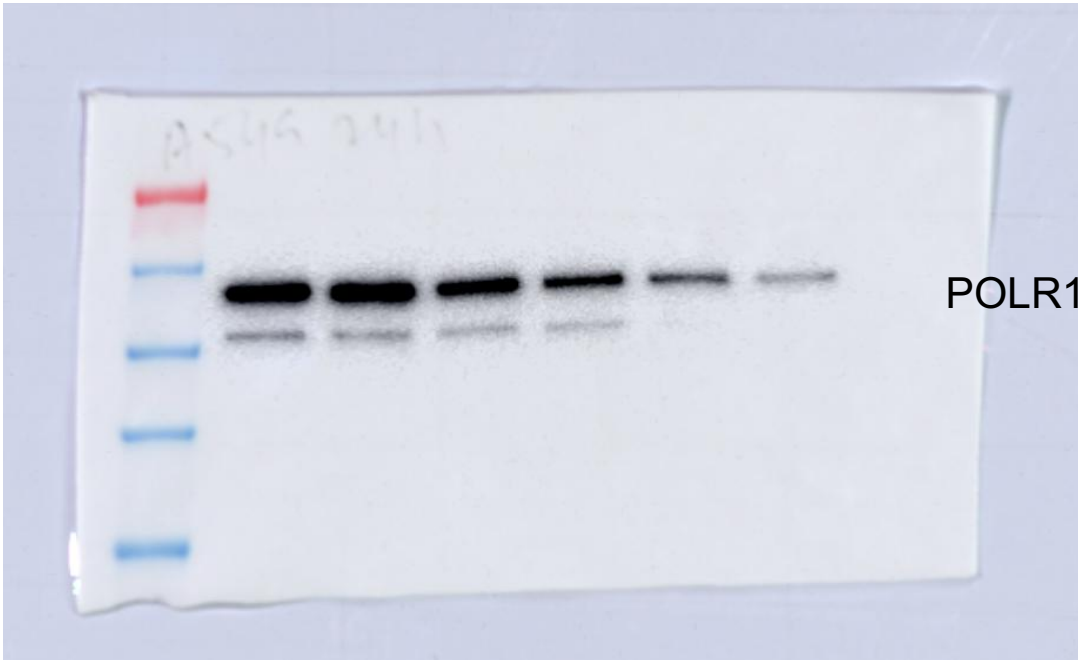

POLR1A

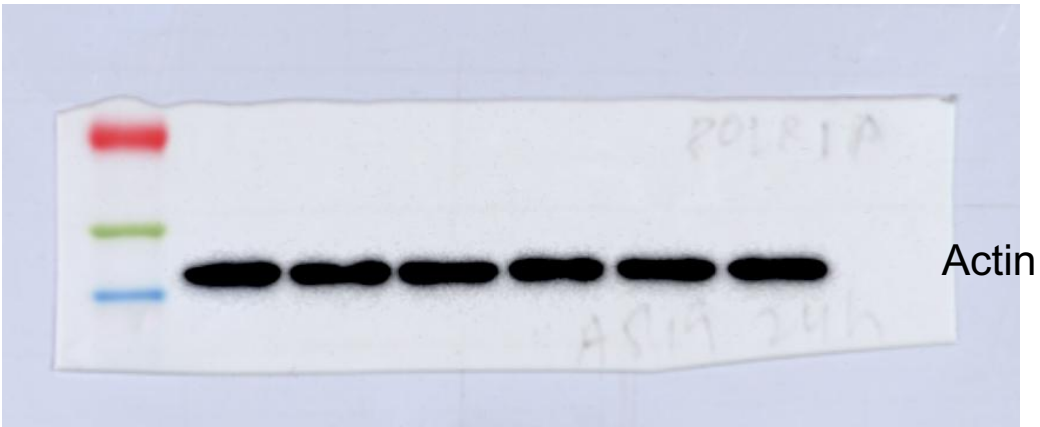

Actin

A549 POLR2A 24h

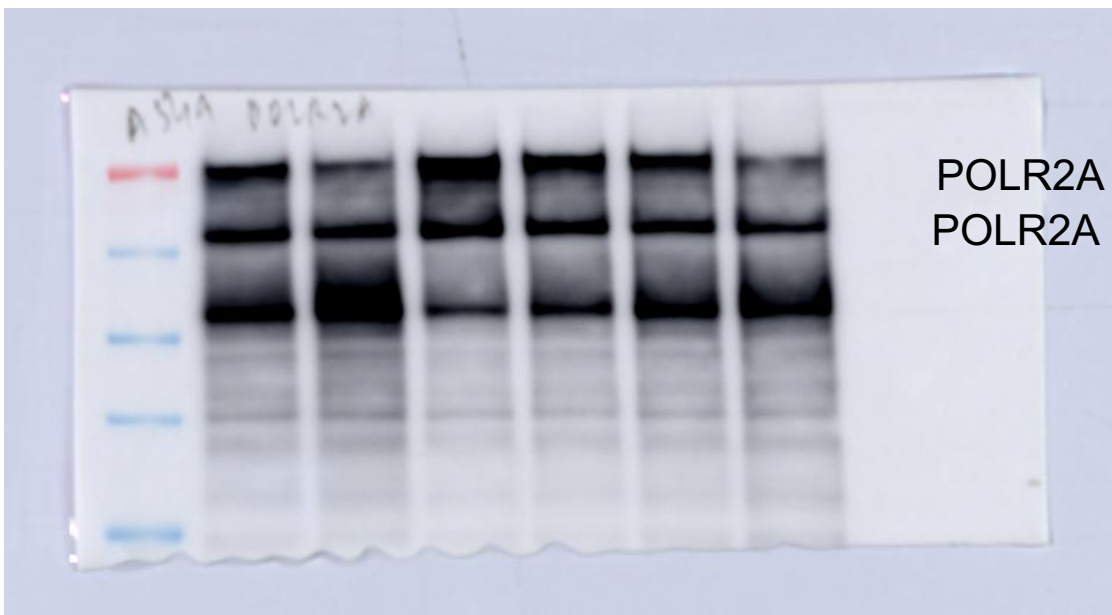

POLR2A IIo
POLR2A IIa

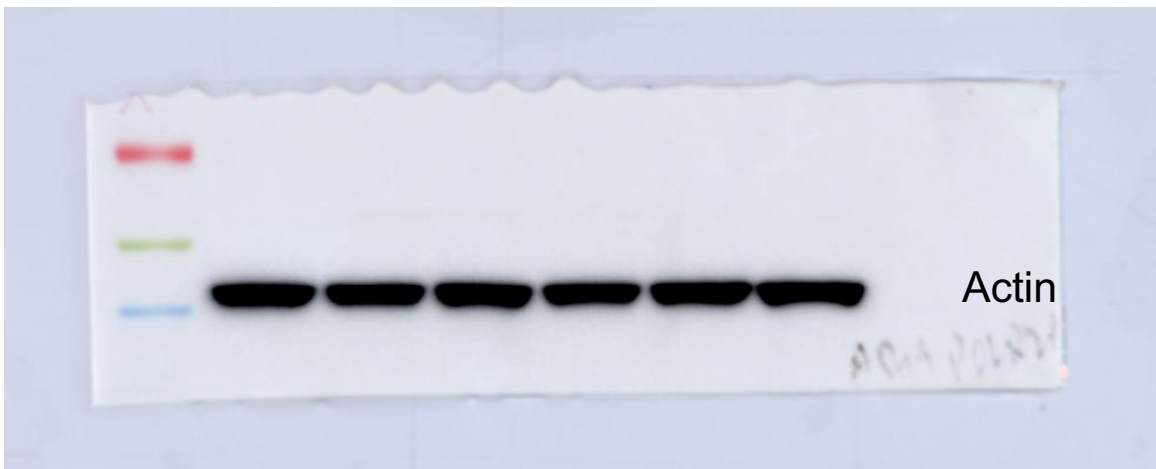

Actin

MCF-7 3h POLR1A

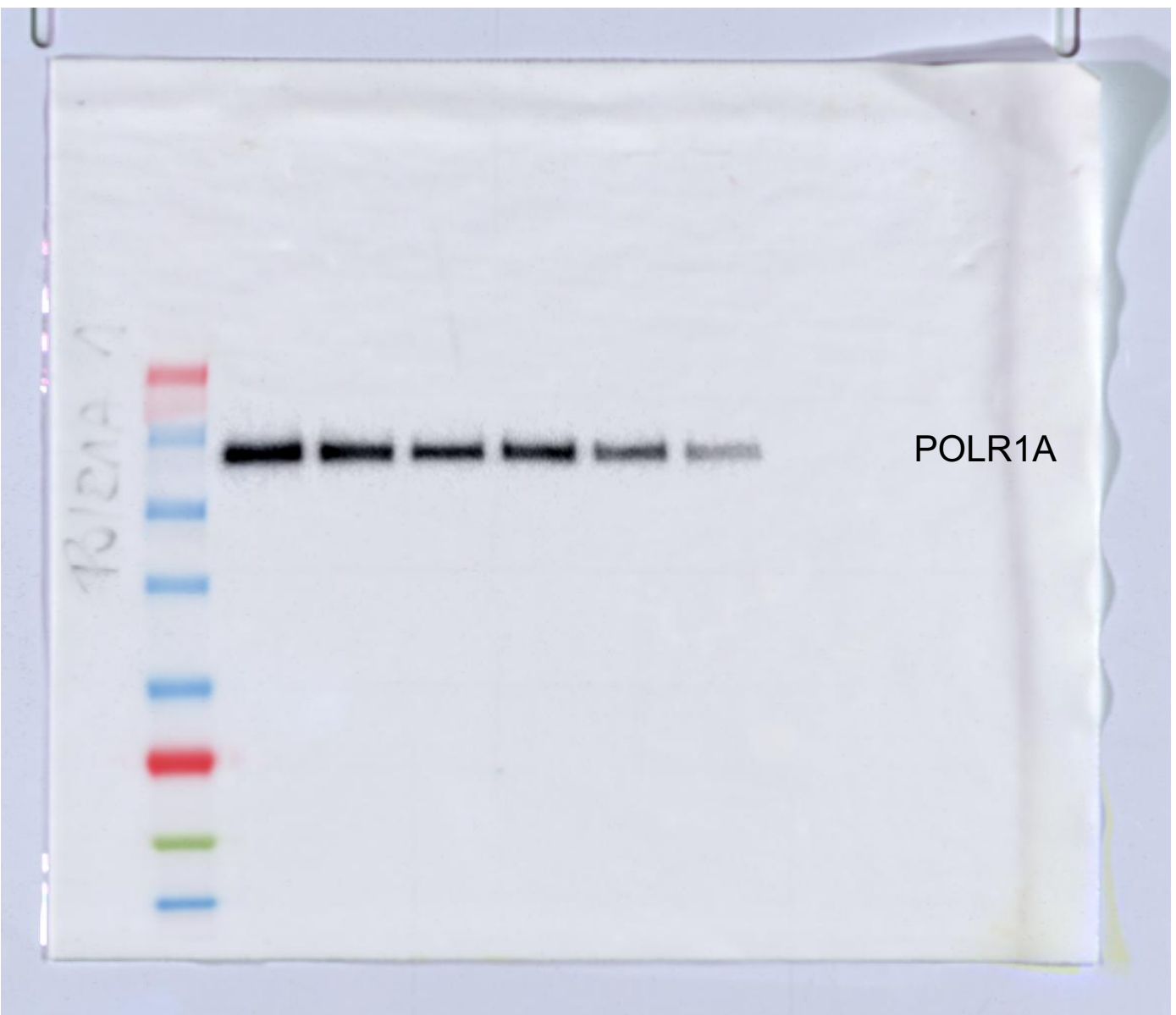

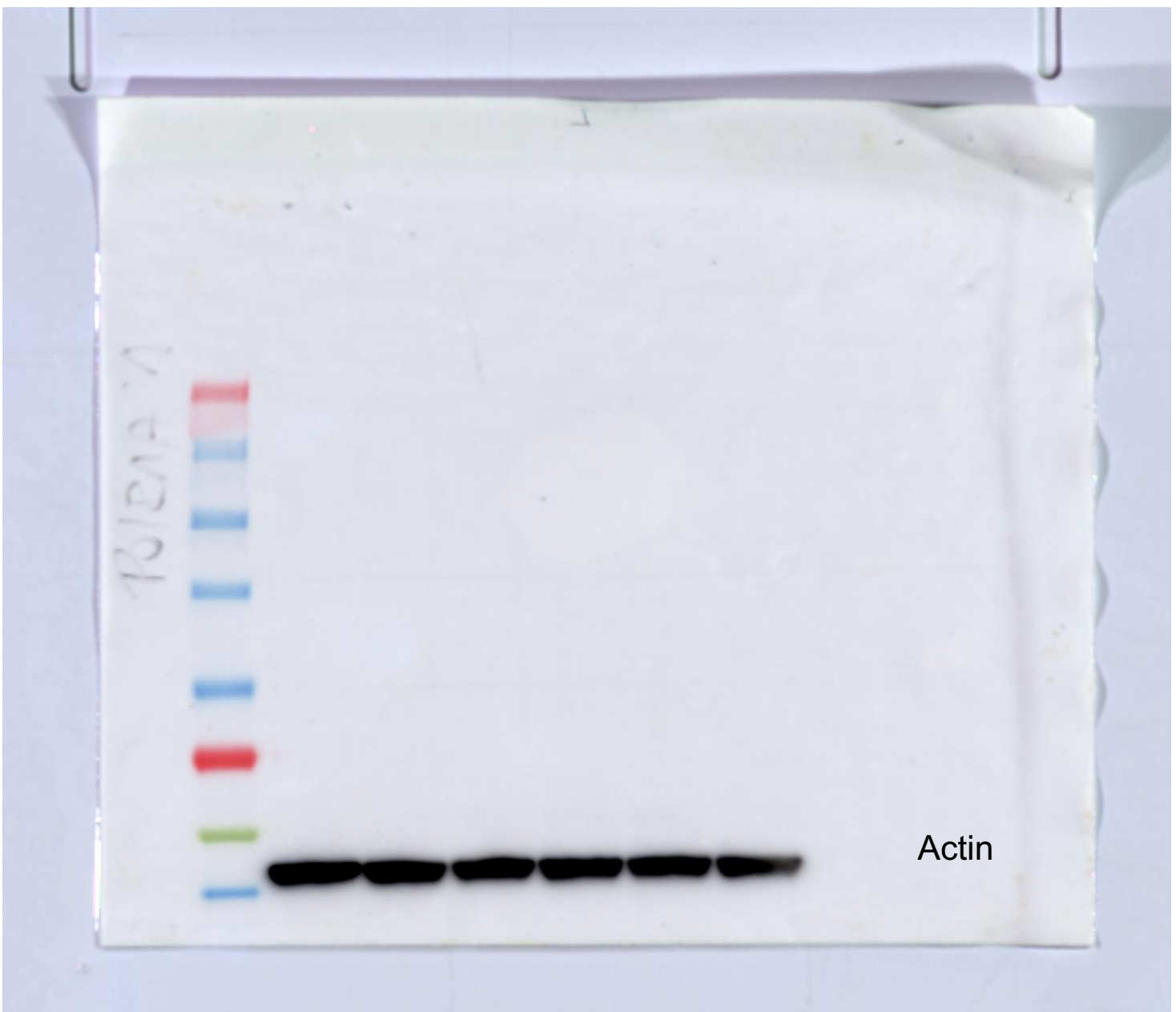

MCF-7 3h POLR2A

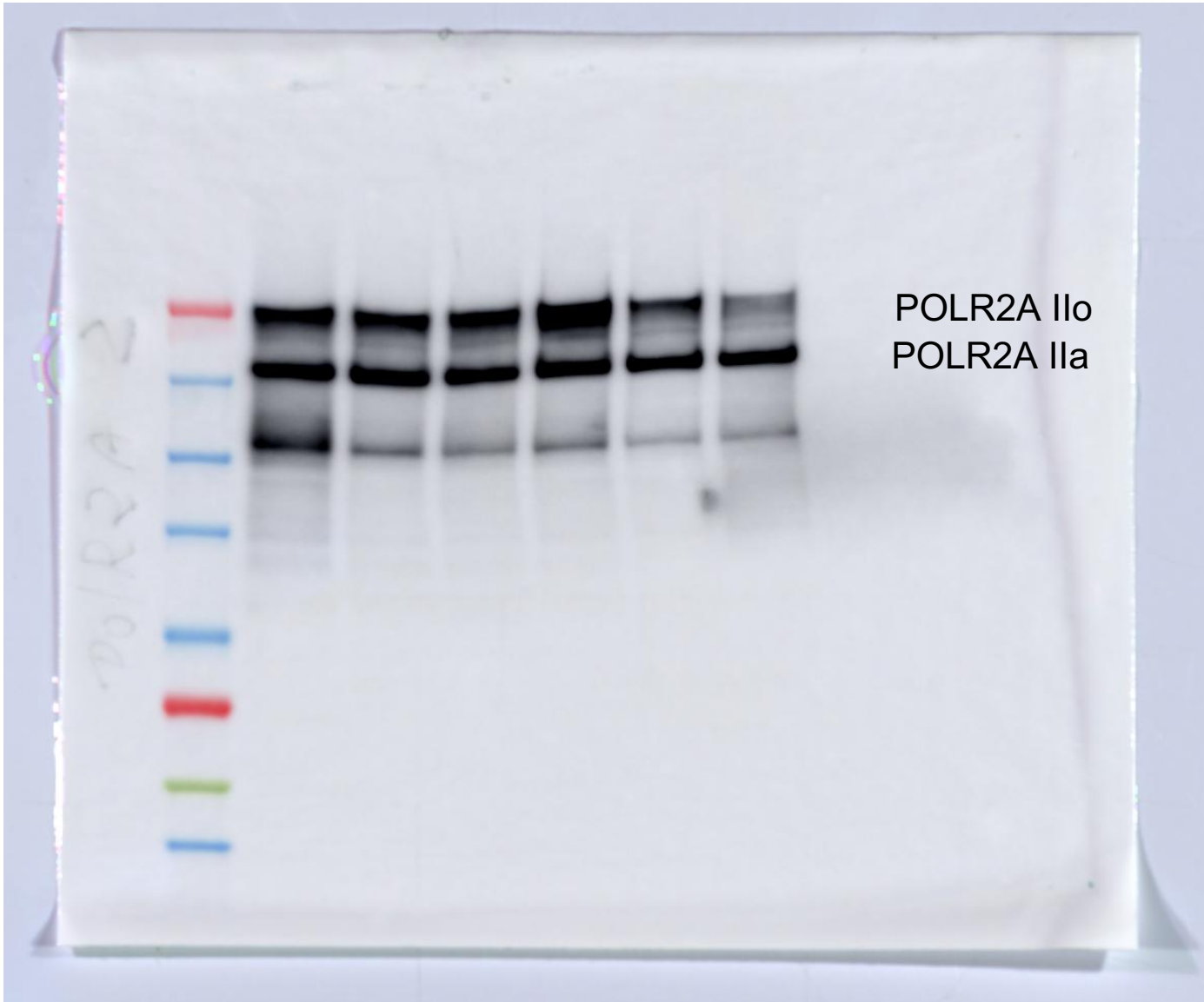

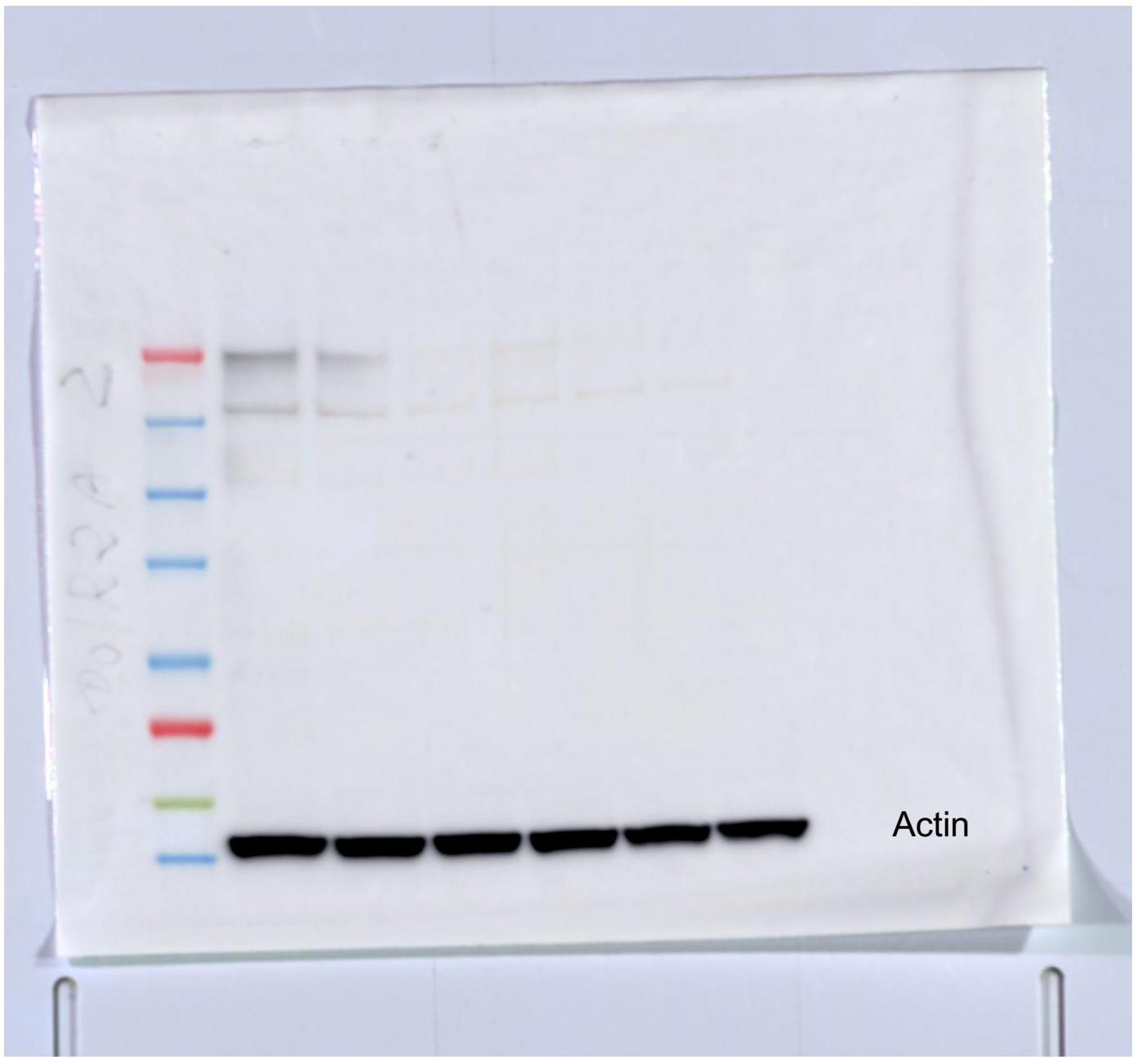

MCF-7 24h POLR1A

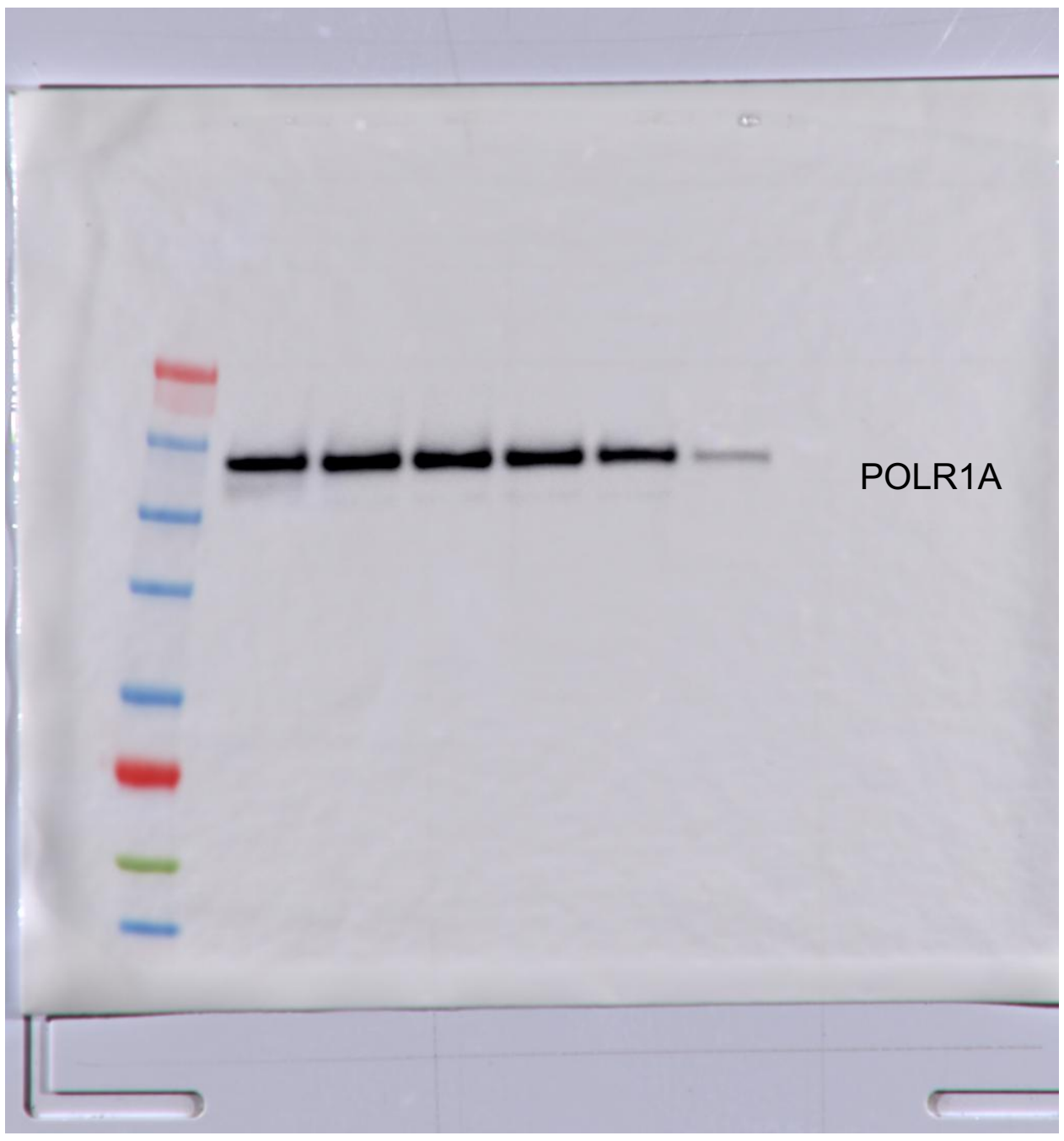

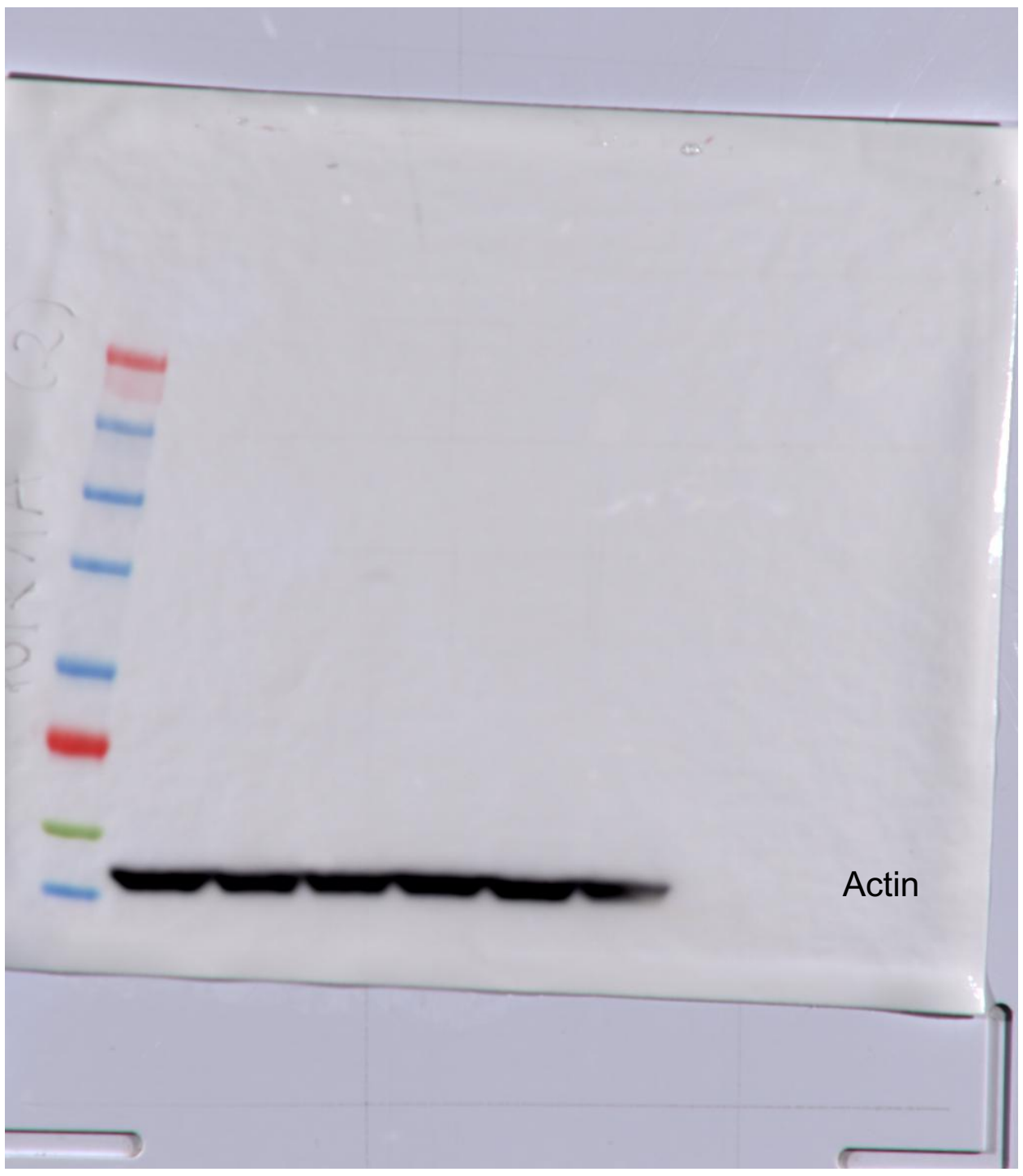

MCF-7 24h POLR2A

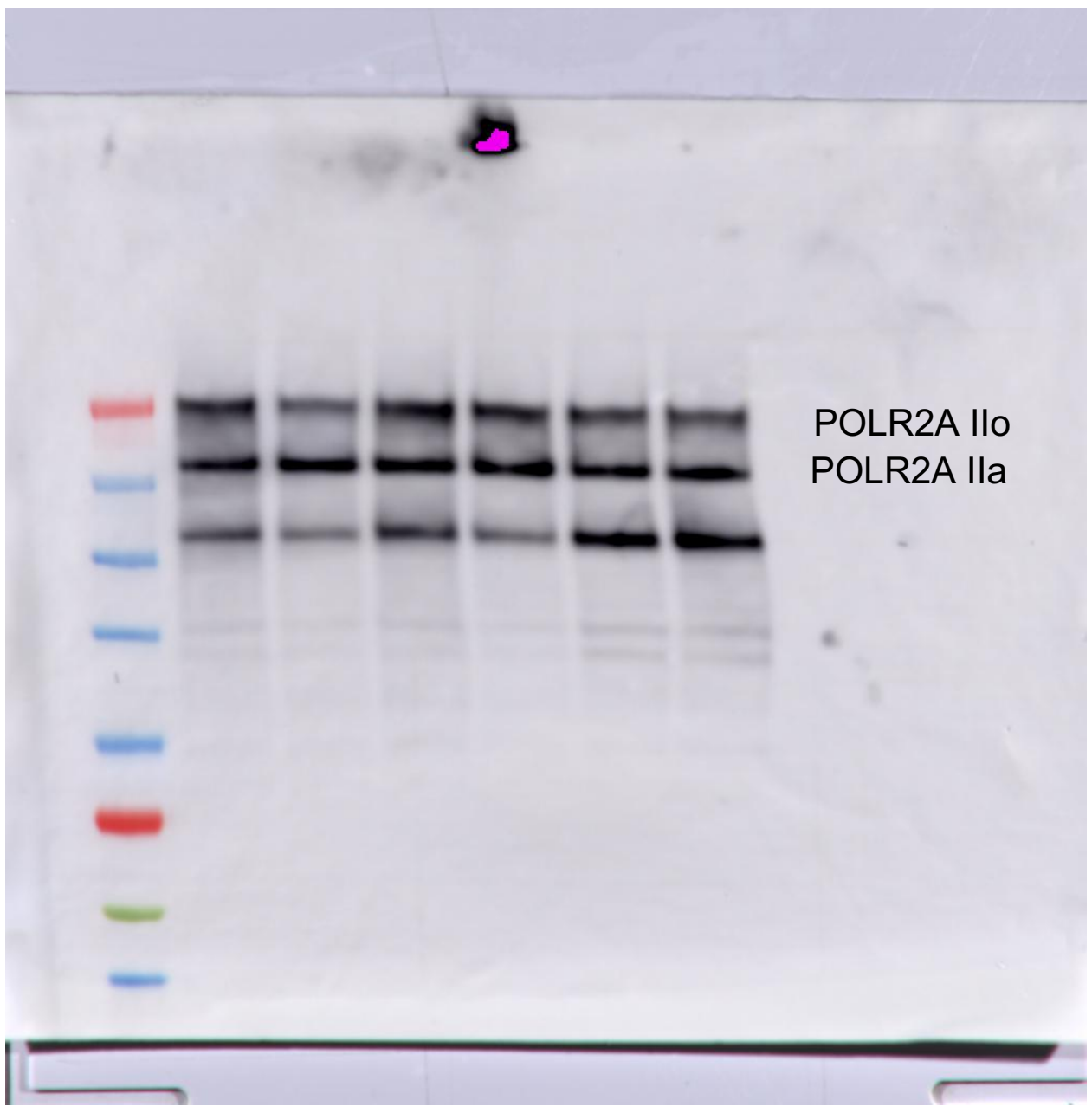

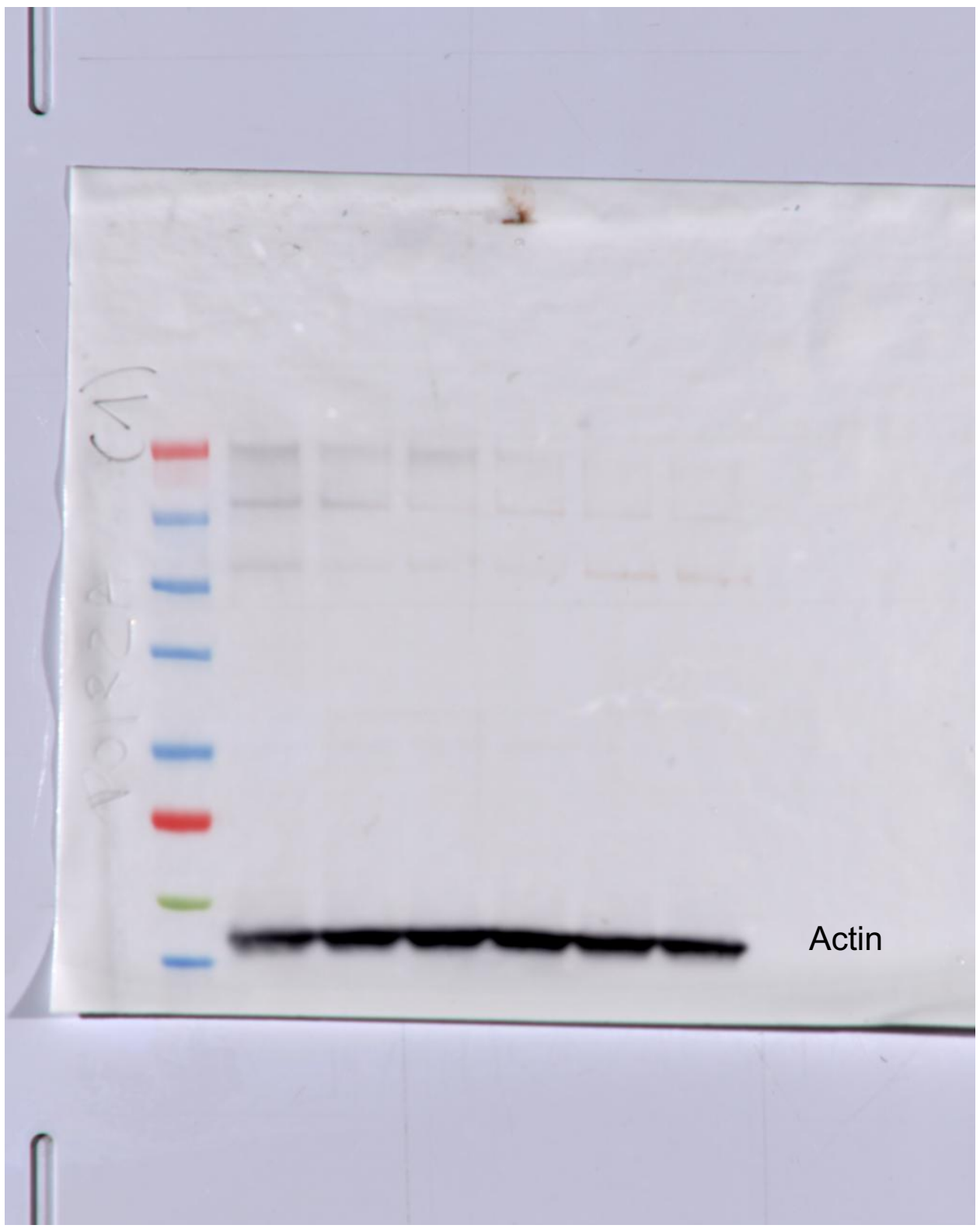

H4 3h POLR1A

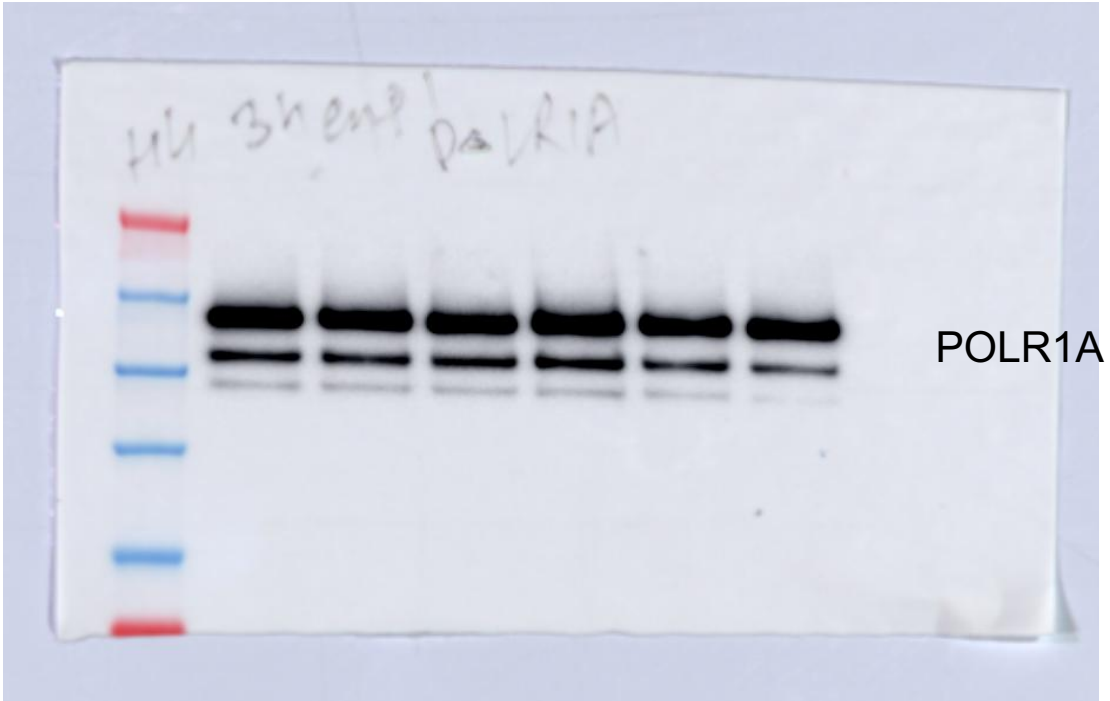

POLR1A

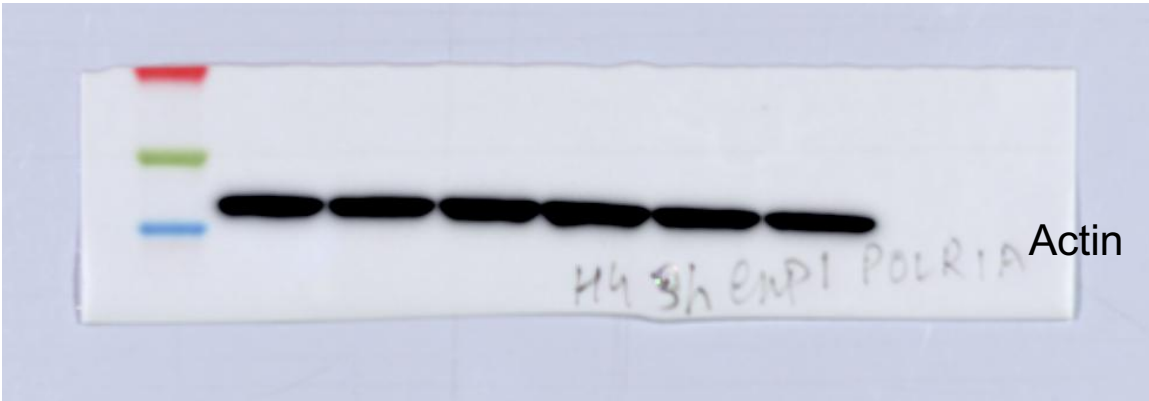

Actin

H4 3h POLR2A

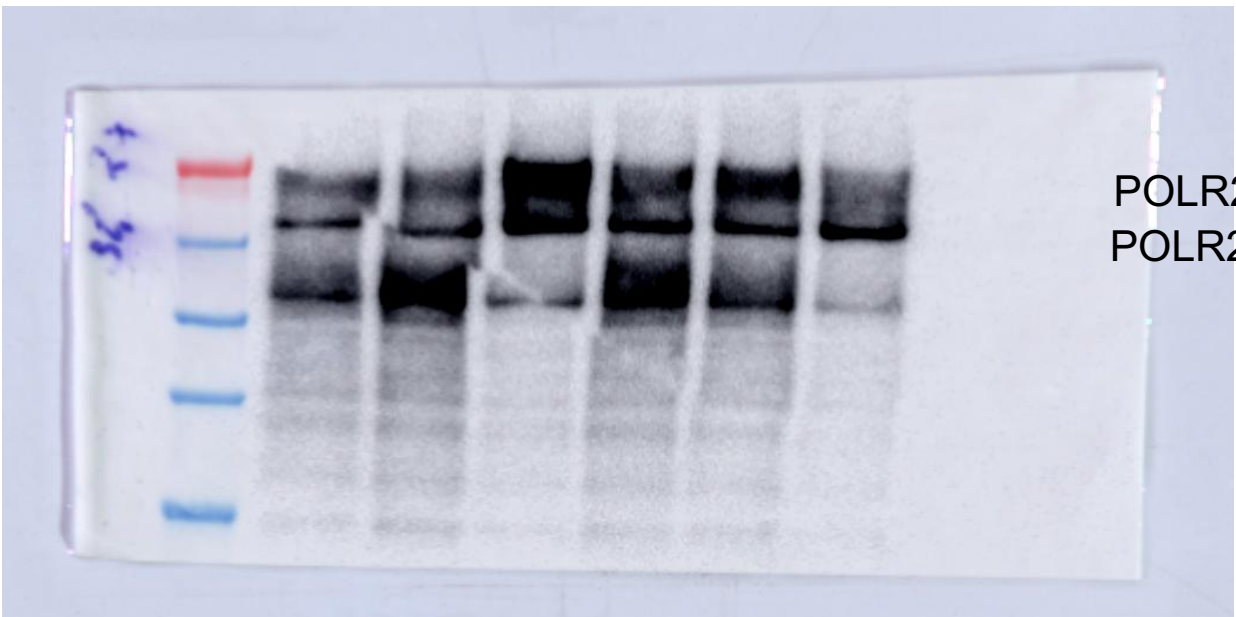

POLR2A IIo
POLR2A IIa

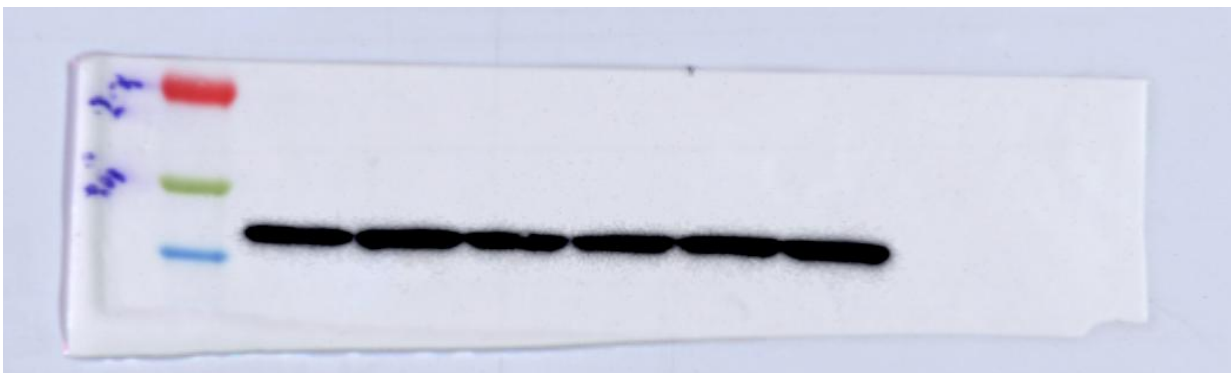

Actin

H4 24h POLR1A

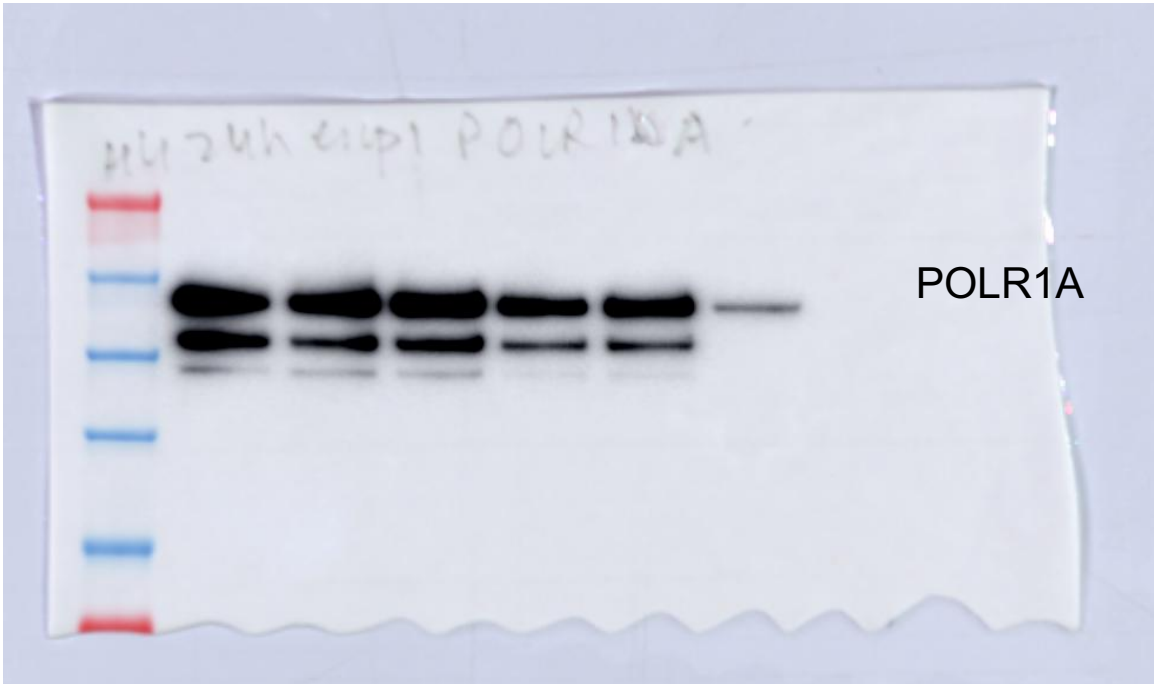

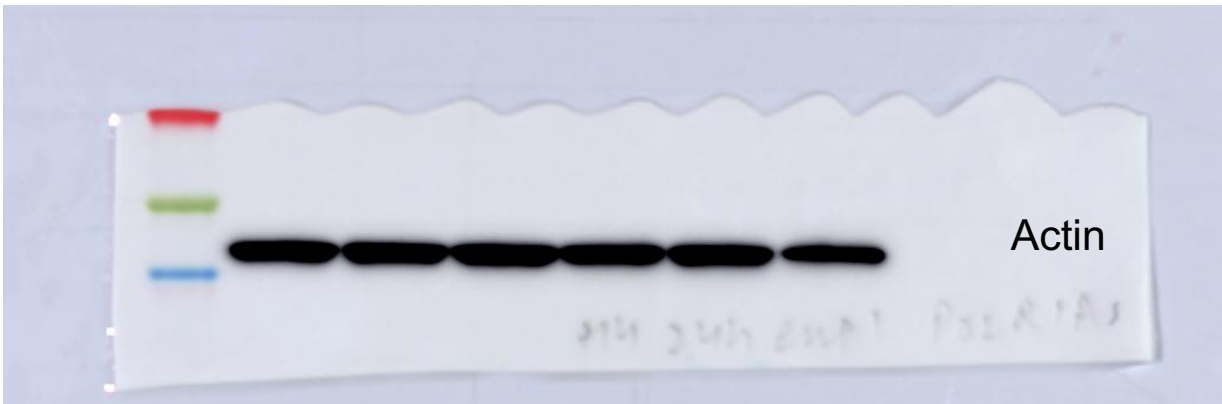

H4 24h POLR2A

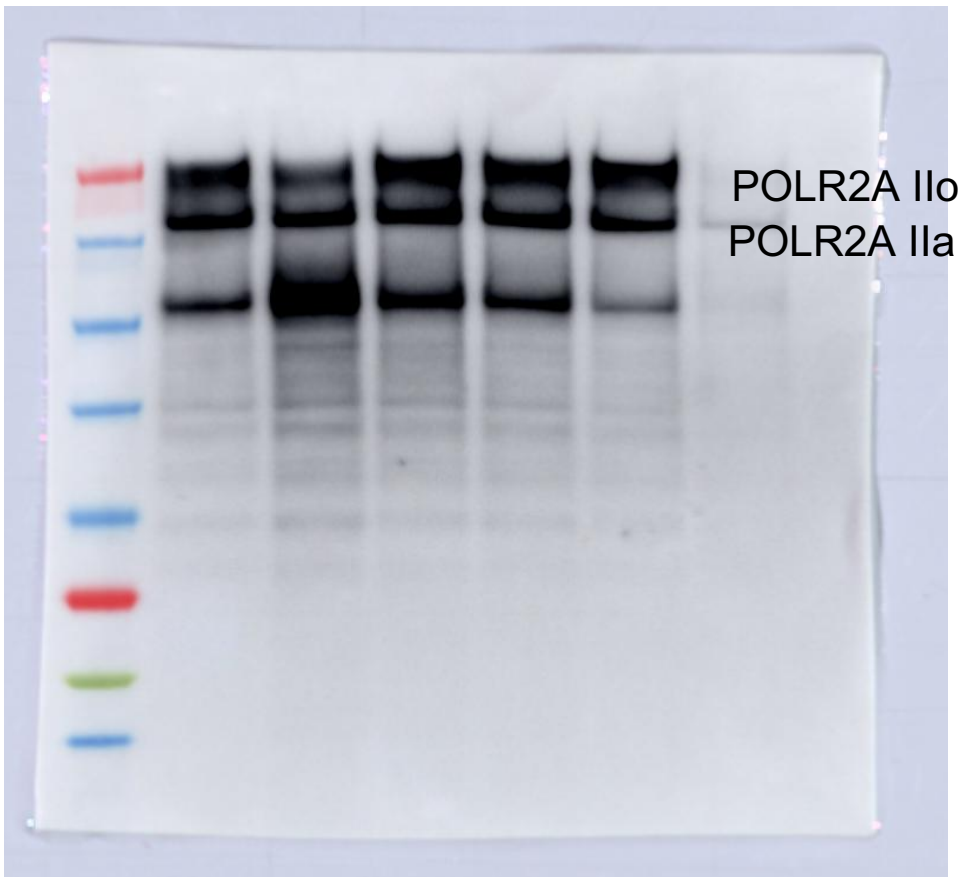

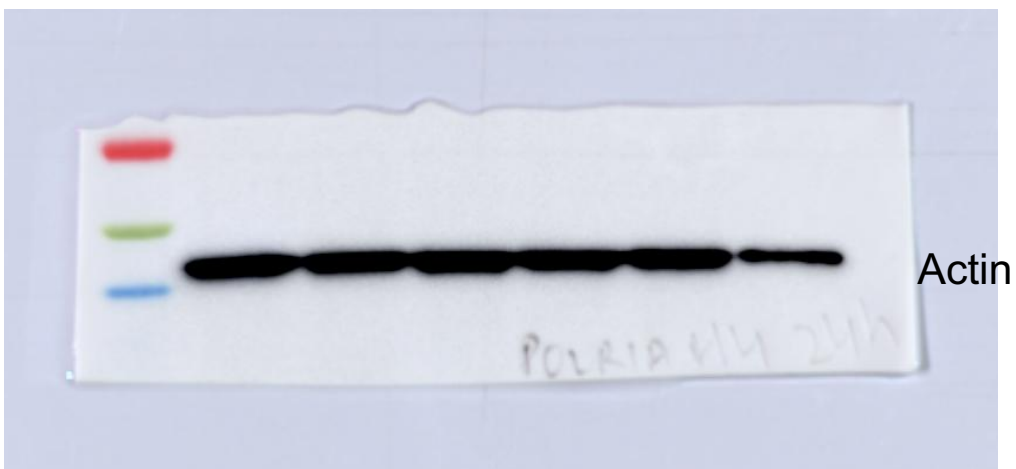

BJ Fibroblasts 3h POLR1A

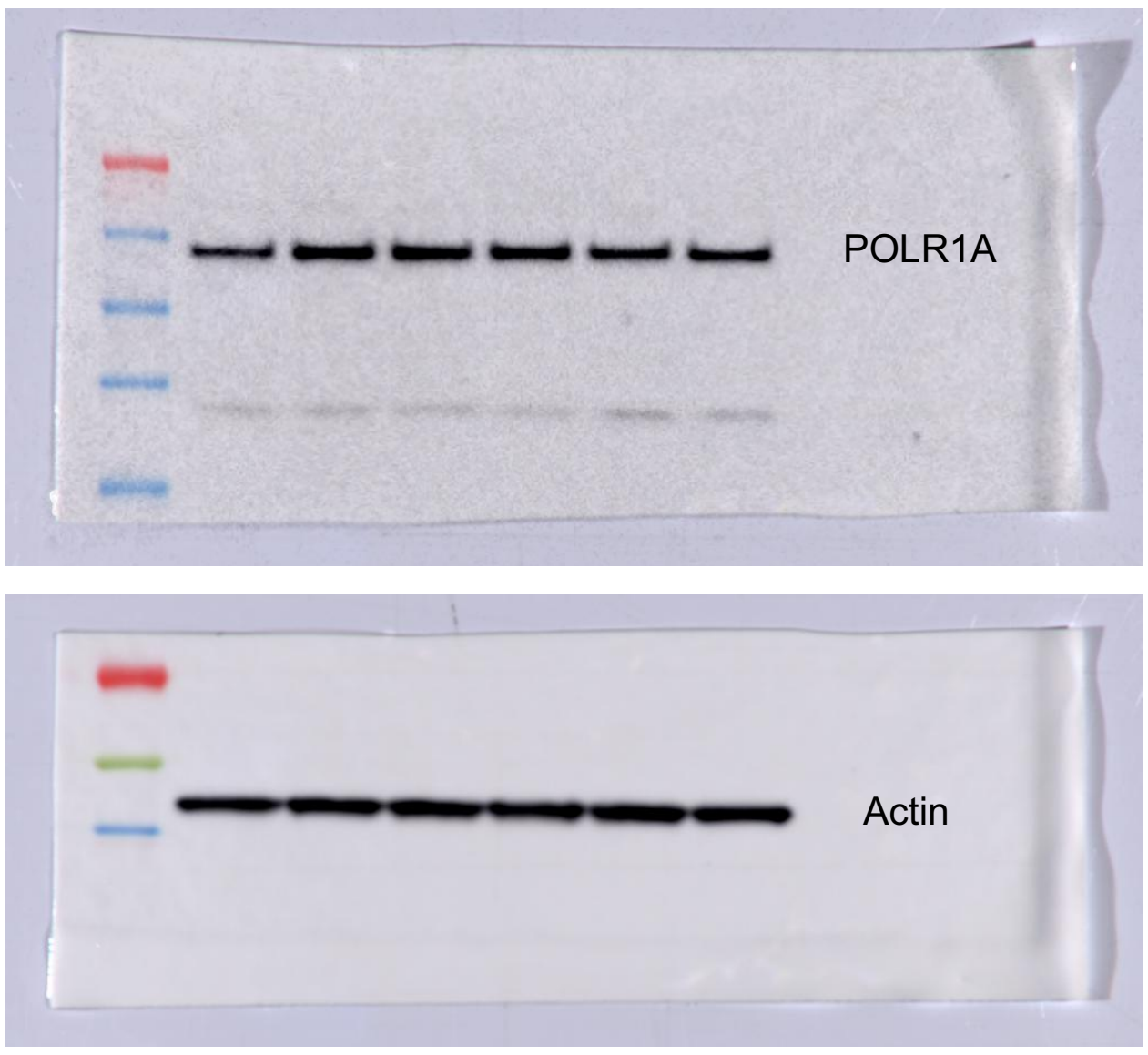

BJ Fibroblasts 24h POLR1A

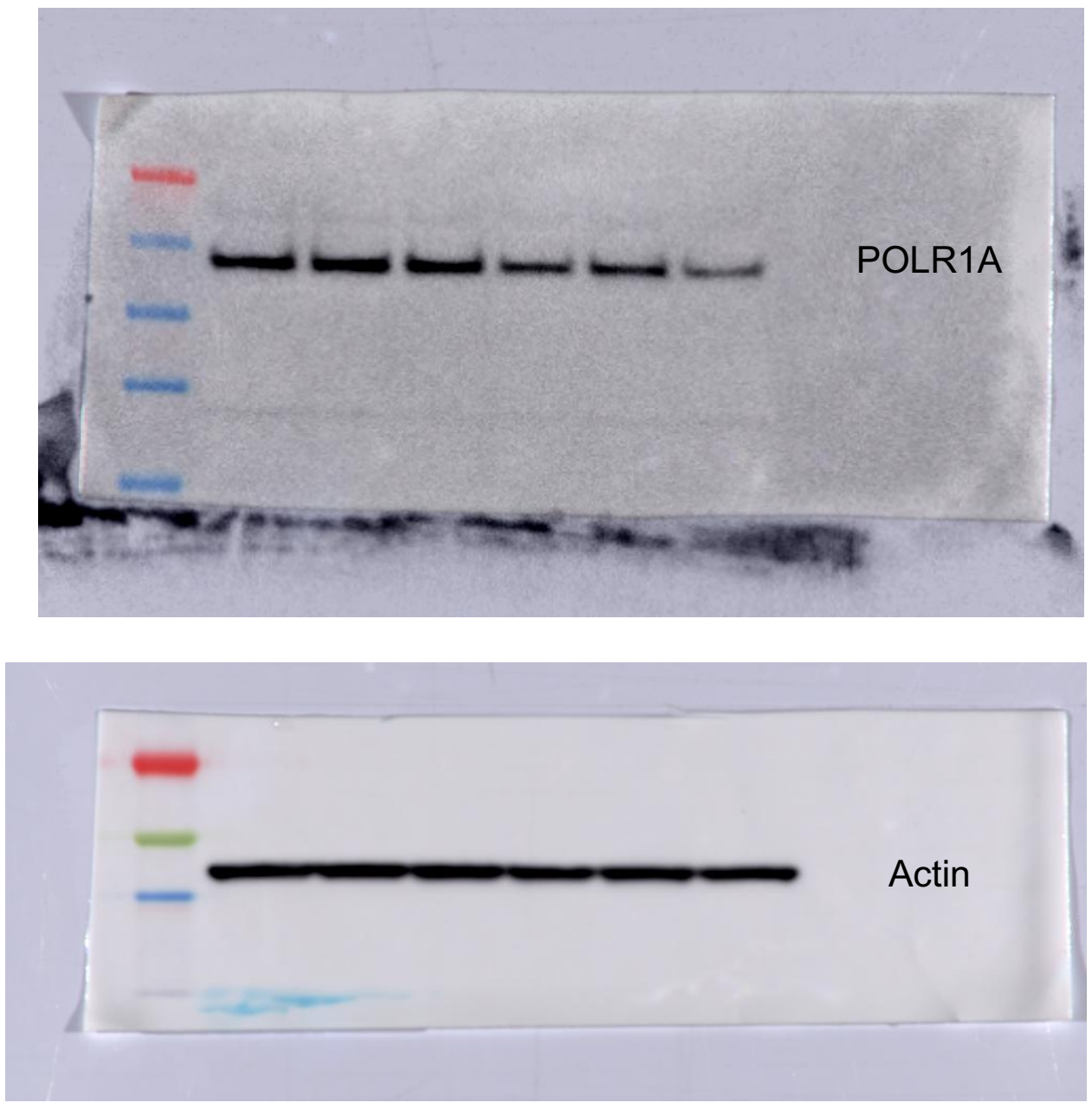

Supplementary Figure S5:

A549 3h

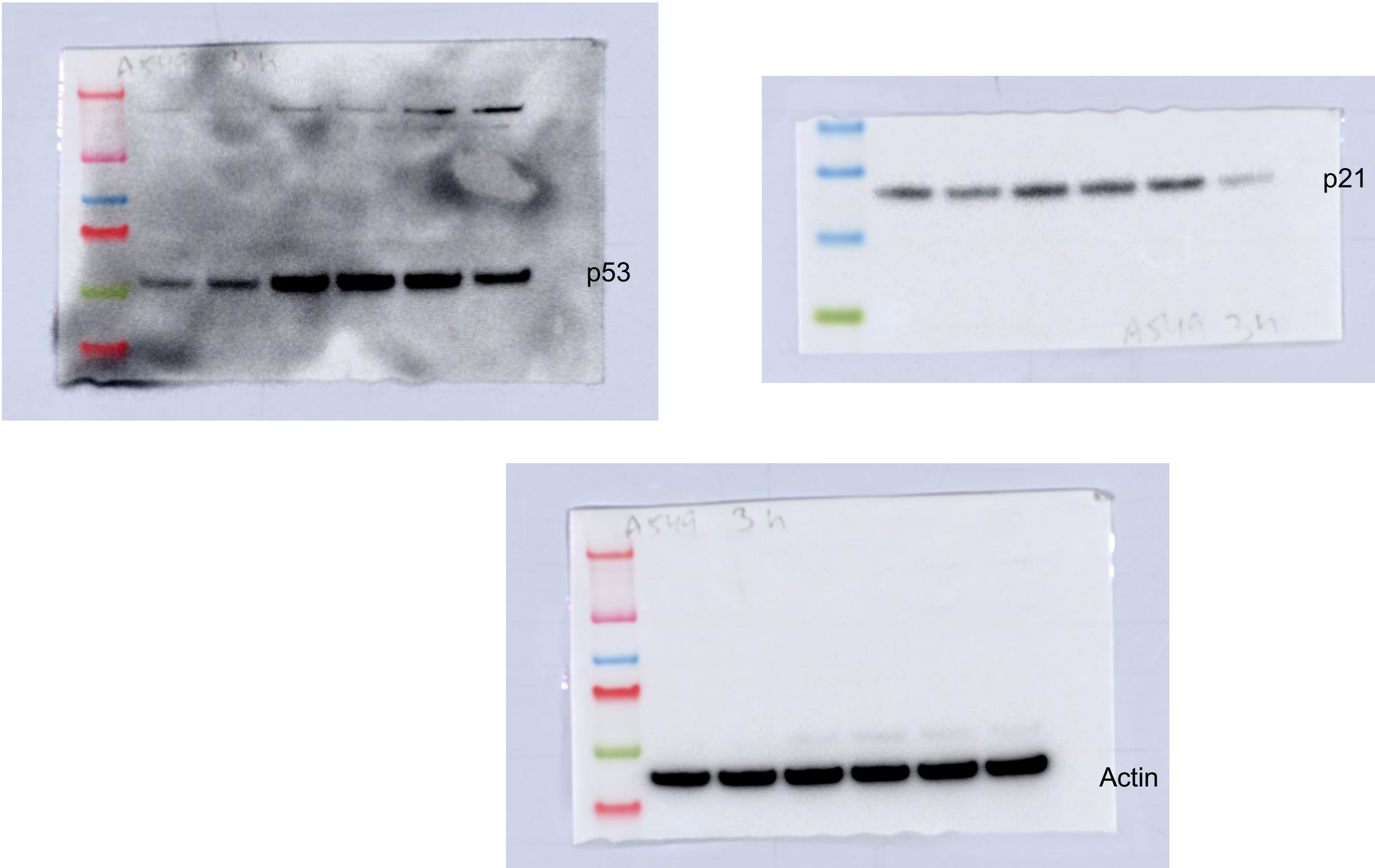

A549 24h

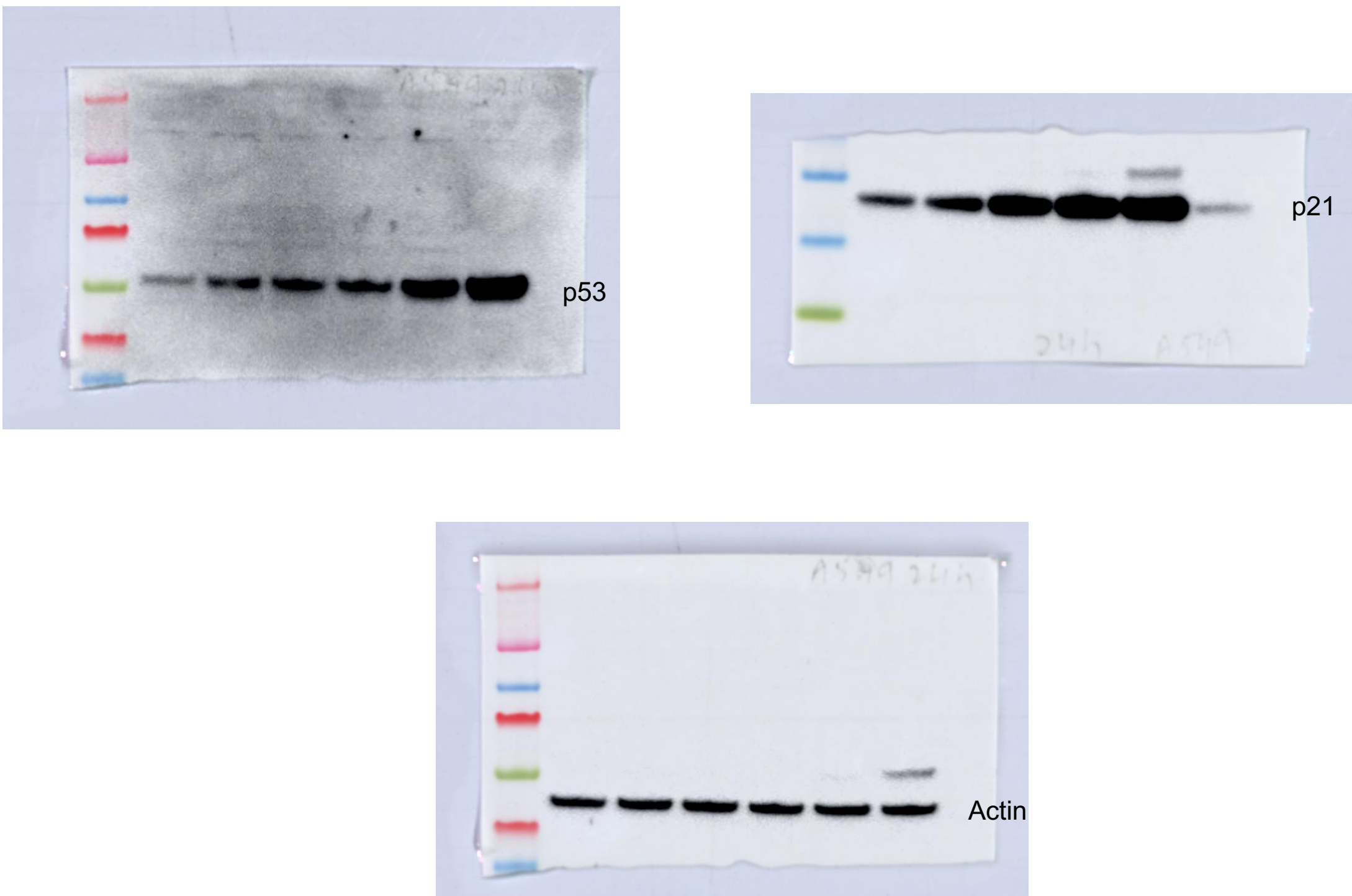

MCF-7 3h

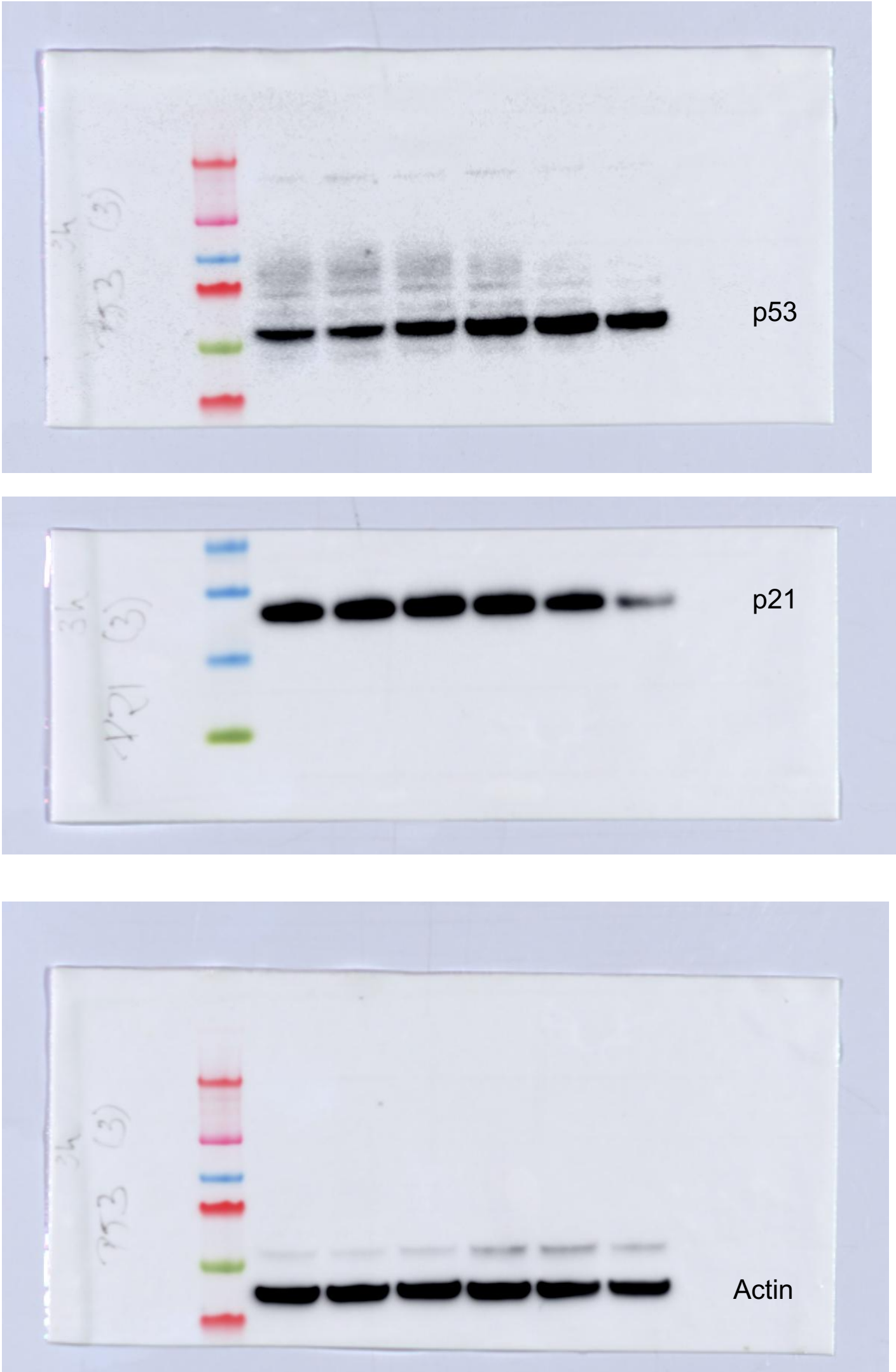

MCF-7 24h

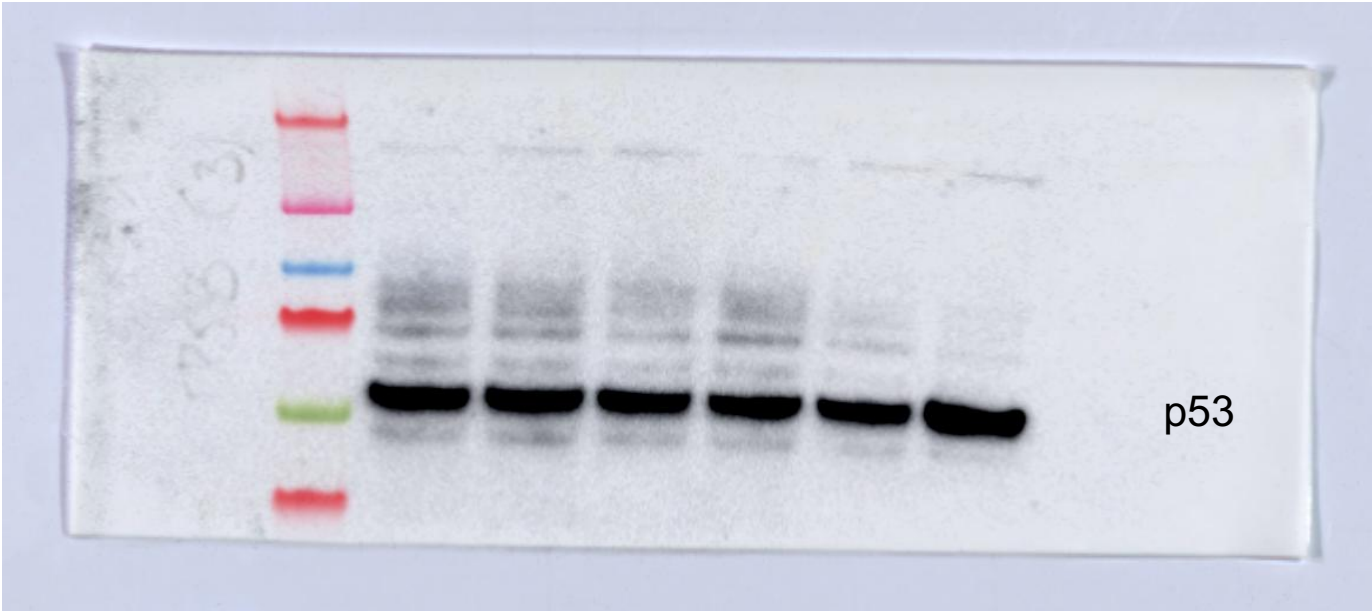

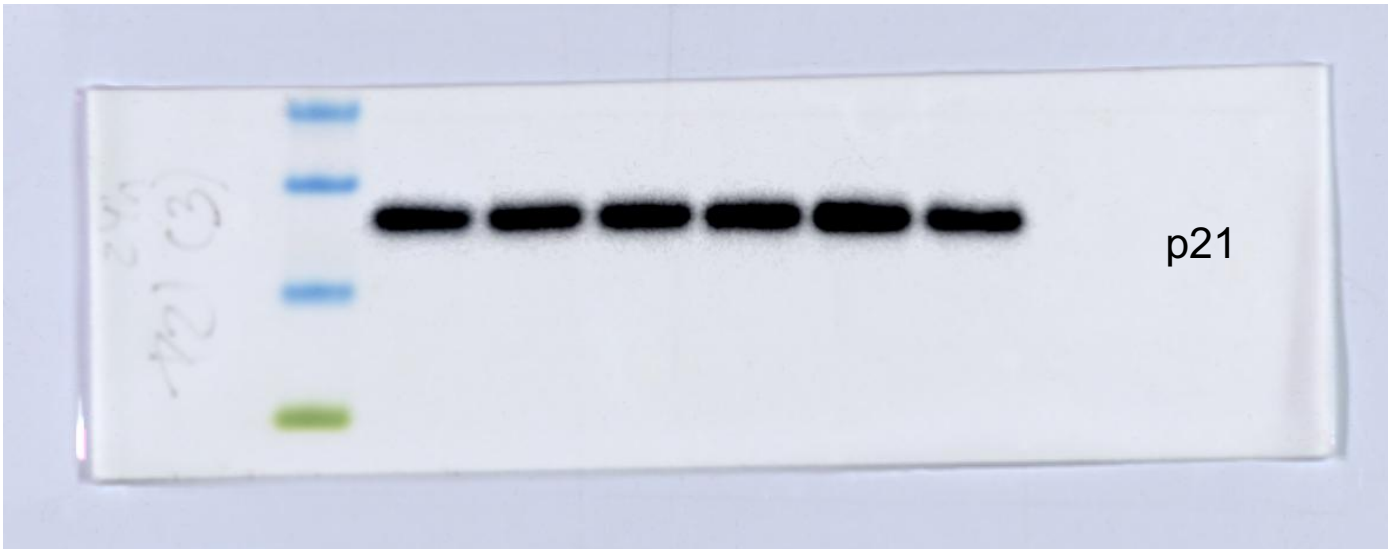

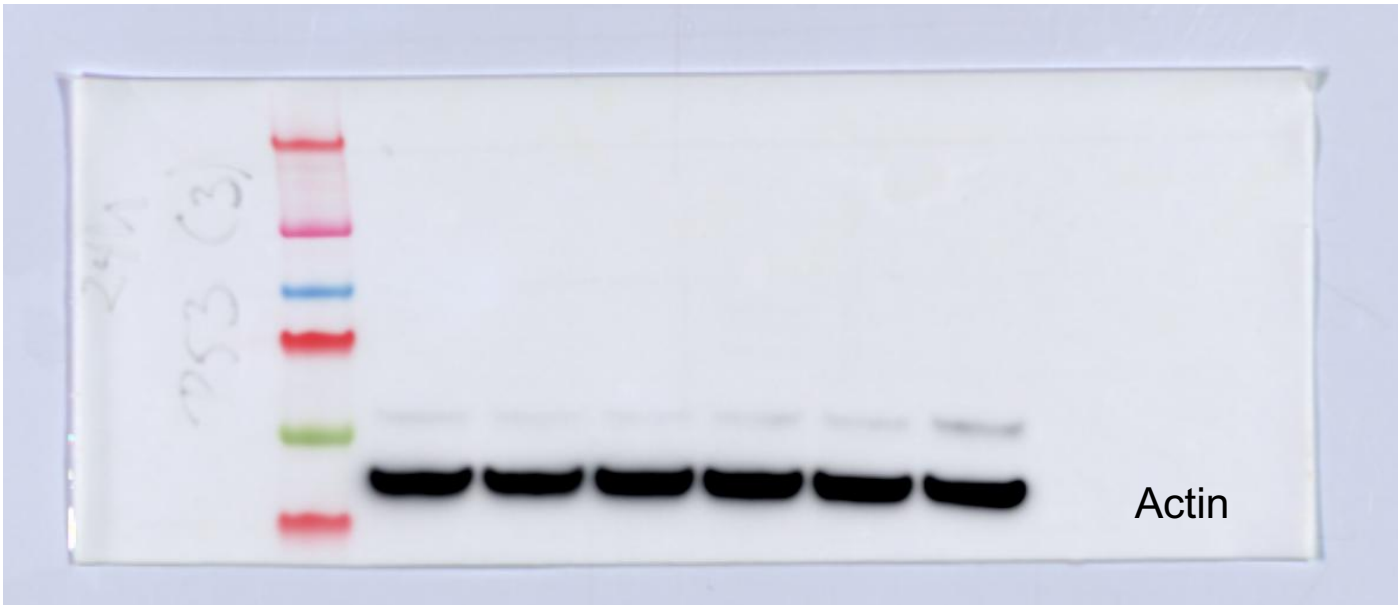

H4 3h

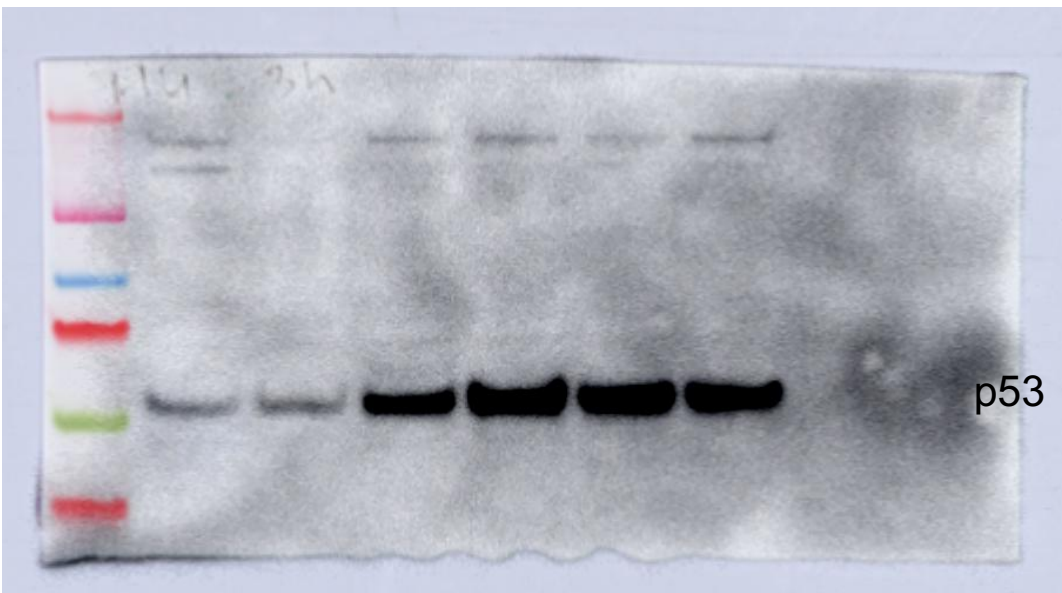

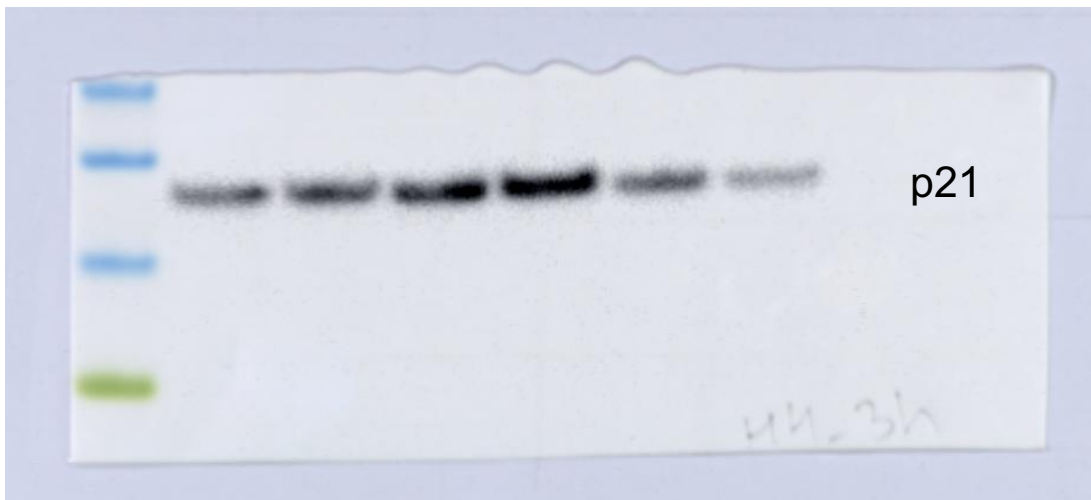

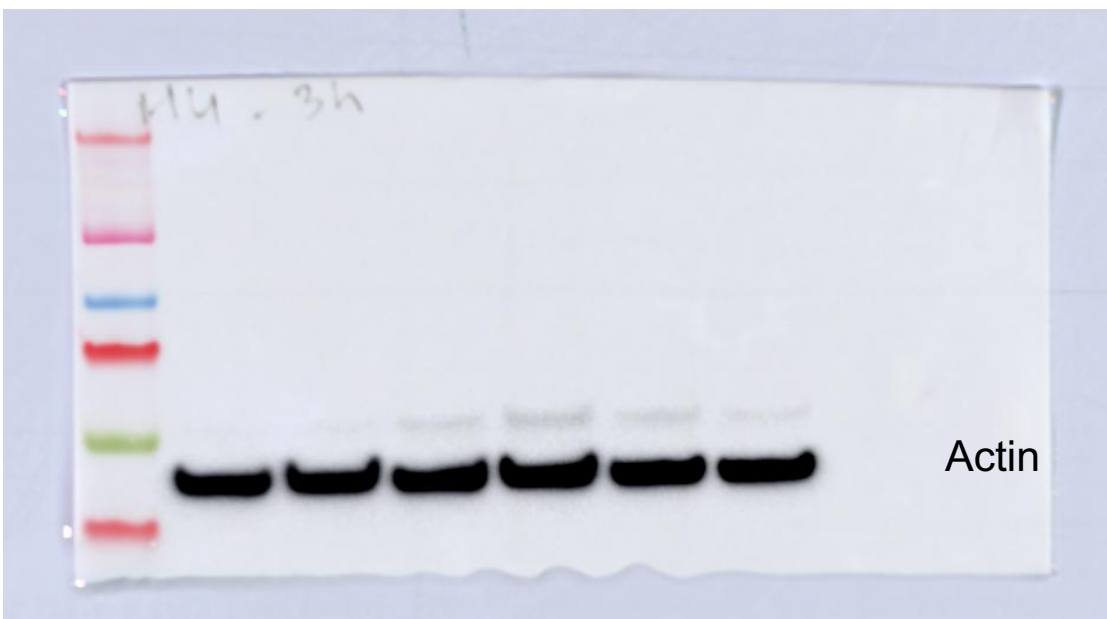

H4 24h

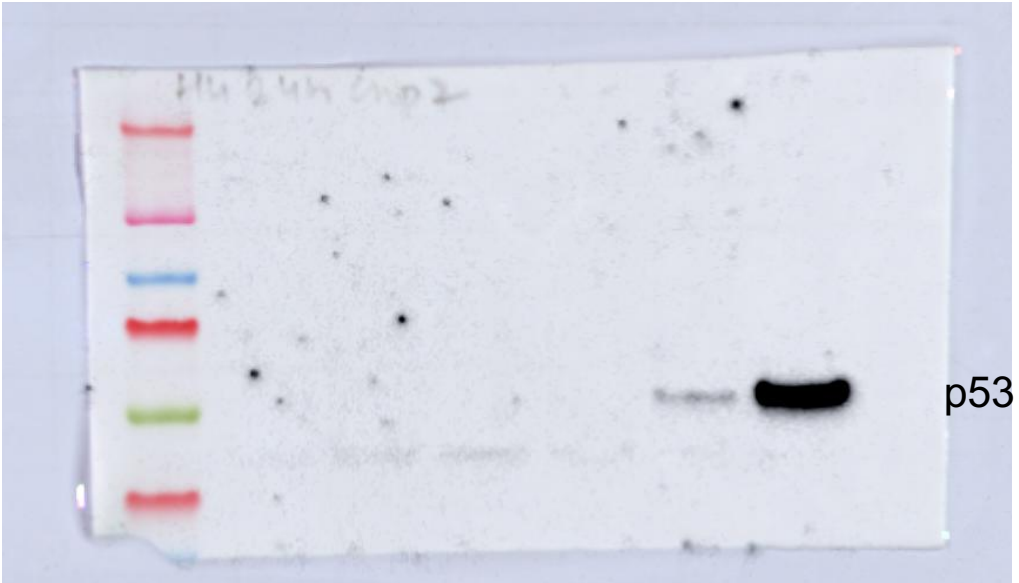

p53

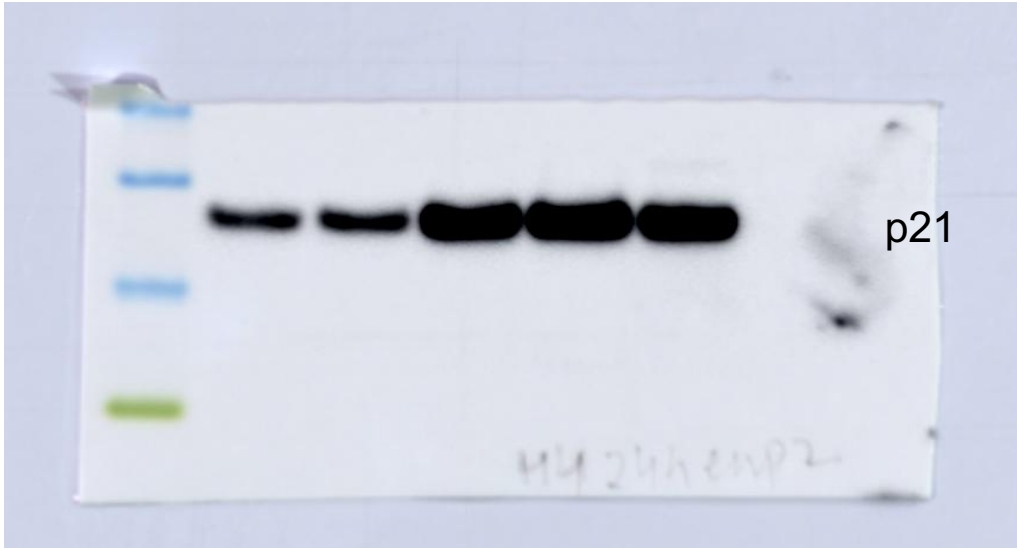

p21

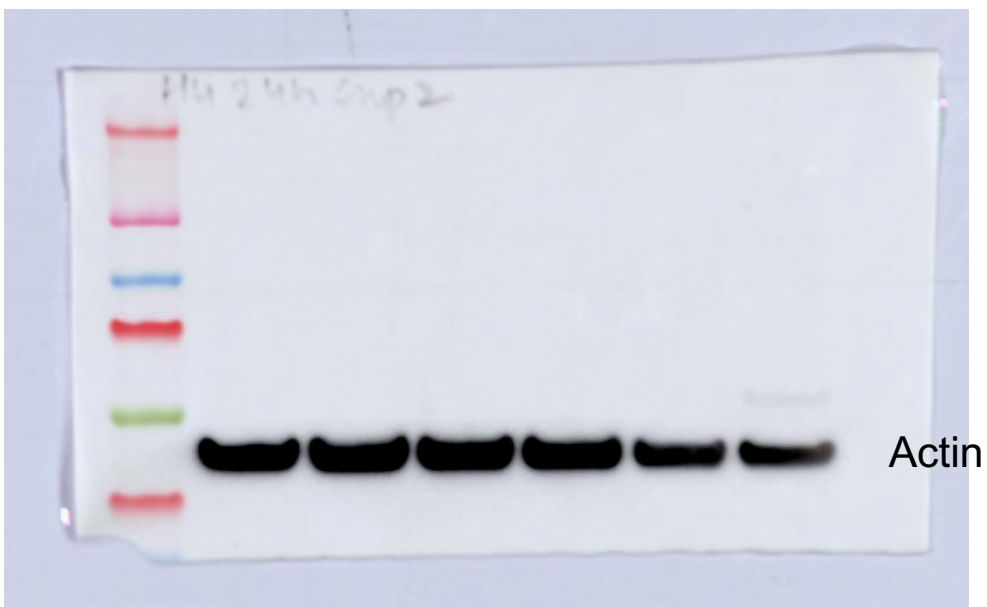

Actin
