# Supplementary material for: JP-1302 inhibits RNA polymerase I transcription and induces nucleolar stress in cancer cell lines
Source: Cell Death Discov. 2026 Sep 30;12:389. doi: 10.1038/s41420-026-03381-8 (PMC13627690; doi:10.1038/s41420-026-03381-8)
Supplement: Supplementary file 2 [file 41420_2026_3381_MOESM2_ESM.pdf]

Supplemental information:

Contents:

1. Supplemental methods
2. Supplemental tables
3. Uncropped Ethynyl Uridine (EU) incorporation and immunofluorescence images
4. Supplemental figures

1. Supplemental methods:

Ethynyl uridine incorporation and immunofluorescence

5000 U2OS cells/well in a 96-well plate were treated with DMSO or varied concentrations of JP-1302 for 3h, followed by labelling with 1 mM ethynyl uridine (EU) for 1h and Click reaction using Click-iT^{TM} RNA Alexa Fluor^{TM} 488 imaging kit (ThermoFisher Scientific). For immunofluorescence imaging, the crosslinked and permeabilized cells were incubated with antibodies at 4°C after blocking for 1 h with 5% BSA in DPBS. For phospho H2A.X immunofluorescence, the crosslinked cells were blocked overnight with 5% BSA in DPBS. The cells were further incubated with fluorescently labelled secondary antibody for 1 h at room temperature and counter-stained with Hoechst 33342 (1:2000 dilution). Images were captured using InCell Analyzer 2200 (GE Healthcare Life Sciences) using 9 evenly separated fields per well, with field positions being constant through all wells. The images were analysed using Cell Profiler 4.2.5. NCL signal was used to mark the nucleolar boundaries and separate nucleolar RNA (rRNA) signal. Statistical differences in EU intensity between groups were calculated using One-way ANOVA, after removing outliers using inbuilt application in GraphPad Prism. Statistical significance for phospho H2A.X foci was calculated using t-test for each treatment condition with control.

Molecular docking

The crystal structure of ellipticine bound to DNA hexamer d(CGATCG)$_2$ (PDB ID: 1Z3F) was retrieved from the RCSB Protein Data Bank and used as the structural template for intercalative docking. The DNA duplex was used as a rigid receptor, retaining the ellipticine-induced unwinding and unstacking of the target CpG step. Ellipticine, cobalt ions and water molecules were removed. Polar hydrogens were added and Gasteiger partial charges were assigned using Open Babel. The 2D structure of JP-1302 was obtained as the dihydrochloride salt (SMILES: CN1CCN(CC1)C2=CC=C(C=C2)NC3=C4C=CC=CC4=NC5=CC=CC=C53.Cl.Cl).

The chloride ions were removed and the distal N-methylpiperazine nitrogen was protonated to generate the physiologically relevant mono-cationic species. 3D conformers were generated with RDKit. The ligand was converted to PDBQT format with Meeko v0.7.1, which assigned Gasteiger-type partial charges, AutoDock atom types. Docking was performed with AutoDock Vina v1.2.5 (Eberhardt et al., 2021, J. Chem. Inf. Model. 61:3891–3898; Trott & Olson, 2010, J. Comput. Chem. 31:455–461). The search space was centered on the centroid of the crystallographic ellipticine ligand at the A1:B6/A2:B5 CpG step (x = 0.87, y = 17.49, z = 46.28 Å), using a cubic grid of 22 × 22 × 22 Å. Docking parameters were set to exhaustiveness = 32, num_modes = 20, energy_range = 4 kcal/mol, random seed = 2026. The DNA receptor was held rigid and all rotatable bonds of JP-1302 were sampled as flexible. Vina returned 19 output poses and the top-ranked pose (–7.65 kcal/mol) was used for structural analysis. Figures were generated in PyMOL v2.5.0. The docked pose was validated by comparing its position and orientation relative to the flanking base pairs against the crystallographic ellipticine reference.

Fluorescence Intercalator Displacement (FID) Assay

FID assay was performed using a panel of deoxynucleotide hairpins (obtained from Espinoza et al., 2019, Cell Death Differ. 27:773-789) spanning a range of GC content (0, 12.5, 25, 37.5, 50, 62.5, 75 and 87.5%). Assays were carried out in 384-well plates in Tris reaction buffer (0.1 M Tris-HCl, pH 8.0). Ethidium bromide (EtBr; final concentration 6 µM) was dispensed into each well and plates were protected from light for the remainder of the assay. The deoxyoligonucleotide hairpins (final concentration 1.5 µM in reaction buffer with 2% DMSO) were then added, followed by the addition of JP-1302 or the reference GC-selective intercalator actinomycin D (ActD). JP-1302 was tested at 10, 20 and 50 µM. Plates were incubated for 30 min or 3 h at RT and fluorescence was measured at excitation/emission 545/595 nm. For each oligonucleotide hairpin, the mean fluorescence of the 0% control was subtracted from all wells, and results were expressed as percentage of retained EtBr–DNA fluorescence: (compound-treated well / mean 100% control for the corresponding oligonucleotide) × 100.

Chromatin Immunoprecipitation assay

Chromatin immunoprecipitation (ChIP) assay was performed using SimpleChIP® Enzymatic Chromatin IP kit (Cell Signalling Technology, Massachusetts, USA). 1×10^7 U2OS cells were treated with 1 µM JP-1302 or DMSO for 3 h. The cells were then crosslinked using 3.7% formaldehyde followed by quenching using glycine. The cells were then lysed for nuclei isolation using nuclease provided in the kit and sonicated for 20 cycles with 30 s ON and 30s OFF using Diagenode Bioruptor. Immunoprecipitation was performed using manufacturer's protocol. Primer sequence for rDNA promoter is provided in the **Supplementary Table S4**.

2. Supplemental Tables

Supplementary Table S1: Details on cell lines used in the study.

Cell line	Source	TP53 status	Culture conditions	Authentication	Mycoplasma testing
U2OS	ATCC	WT	Cultured in DMEM GlutaMAX with 10% FBS and 1% Penicillin-Streptomycin, incubated at 37ºC with 5% CO_2 and 5% O_2.	Authenticated at source and Eurofins cell line authentication services	Every month
BJ	ATCC	WT			
HCT116 p53 wild type	B. Vogelstein (Baltimore, USA)	WT			
HCT116 p53 knockout	B. Vogelstein (Baltimore, USA)	KO			
A549	ATCC	WT		ATCC and Uppsala SciLifeLab NGI	
MCF-7	T. Helleday (Karolinska Institutet, Sweden)	WT		Morphology	
A172	ATCC	WT		Eurofins cell line authentication services	
H4	ATCC	WT		Eurofins cell line authentication services	
RKO	ATCC	WT		Eurofins cell line authentication services	
LN229	ATCC	Mut p53		ATCC	
ddp53 U2OS	Jiri Bartek (DCS, Denmark)	WT ddp53		Expression ddp53 and morphology	
RPE1	ATCC	WT		ATCC	

Supplementary Table S2: Details on drugs used in the study.

Chemical	Company	Catalogue number	Solubility
JP-1302	Cayman chemical	25974	DMSO
MG132	Sigma-Aldrich	M7449	DMSO
BMH-21	Sigma-Aldrich	SML1183	DMSO
Etoposide	TopoGen	TG4140	DMSO
Doxorubicin	Sigma-Aldrich	D1515	DMSO
Actinomycin D	Sigma-Aldrich	A1410	DMSO

Supplementary Table S3: Details on antibodies used in the study.

Antibodies	Company	Clone	Catalogue number	Application (dilution)
POLR1A	Cell Signalling Technology	D6S6S	24799	WB (1:1000) ChIP (7 µg)
Rpb1 NTD (POLR2A)	Cell Signalling Technology	D8L4Y	14958	WB (1:1000)
TOP2A (Topo II Alpha)	Bethyl Laboratories	Polyclonal	A300-054A	WB (1:1000) Slot blot (1:1000)
TOP2B (Topo II Beta)	Bethyl Laboratories	Polyclonal	A300-950A	WB (1:1000) Slot blot (1:1000)
SSRP1	Cell Signalling Technology	E1Y8D	13421S	WB (1:1000)
SPT16	Cell Signalling Technology	D7I2K	12191S	WB (1:1000)
Double-stranded DNA	Abcam	35I9	ab27156	Slot blot (1:1000)
p53	Abcam	DO-1	ab1101	WB (1:1000)
p21 Waf1/Cip1	Cell Signalling Technology	12D1	2947	WB (1:1000)
ZMAT3	Proteintech	Polyclonal	10504-1-AP	WB (1:1000)
MDM2	Merck	4B11	OP143	WB (1:1000)
c-MYC	Cell Signalling Technology	D84C12	5605S	WB (1:1000)
RPL5	Abcam	Polyclonal	ab86863	WB (1:1000)
RPL11	Abcam	Polyclonal	ab79352	WB (1:1000)
Nucleolin	Abcam	Polyclonal	ab22758	IF (1:500)
β-actin	Abcam	AC-15	ab6276	WB (1:1000)
Lamin A/C	Santa Cruz	Polyclonal	sc-20681	WB (1:1000)
H2A.X (phospho-Ser139)	Merck Millipore	JBW301	05-636	WB (1:1000) IF (1:500)
Rabbit IgG H&L (Cy5®) - Secondary	Abcam		ab97077	IF (1:1000)
Mouse IgG H&L (Alexa Fluor 488)-Secondary	Life Technologies		A11001	IF (1:1000)
Anti-Rabbit IgG HRP	Sigma-Aldrich		A6154	WB (1:10,000)

Anti-Mouse IGG HRP	Sigma-Aldrich		A9044	WB (1:10,000)
POLR1B	Santa Cruz	4H6	sc-293272	WB (1:500)
POLR1E	Sigma-Aldrich	Polyclonal	HPA022527	WB (1:1000)
POLR1F (TWISTNB)	Sigma-Aldrich	Polyclonal	HPA022416	WB (1:1000)
TAF1C	Abcam	EPR11298	ab178690	WB (1:1000)
UBTF (UBF)	Santa Cruz	F9	sc-13125	WB (1:1000)<br>IF (1:500)
RRN3	Santa Cruz	D9	sc-390464	WB (1:1000)
TOP1	Sigma-Aldrich	1.1A	MABE1084	WB (1:1000)
Fibrillarin	Abcam	Polyclonal	ab5821	IF (1:500)
ADRA2C	Novus Biologicals	Polyclonal	NB100-93554	WB (1:1000)

Supplementary Table S4: Primer sequences

Gene	Forward	Reverse
47S_5´ETS	GAACGGTGGTGTGTCGTT	GCGTCTCGTCTCGTCTCACT
POLR1A mRNA	TTTGCCGTGTATGGCATCGC	TGTCATCTGCTGTAGCGGGG
Beta Actin mRNA	TCACAATGTGGCCGAGGACTTT	AGAAGTGGGGTGGCTTTTAGGA
P21/CDKN1A mRNA	AATCGTCCAGCGACCTTCCT	CTGACTCCTTGTTCCGCTGC
rDNA promoter	AGGAGCGCGGCCGGCTAGCC	CCCTGCGTGTGGCACGGGC
c-MYC mRNA	ATCCCACGCTCTGAACGC	CTCGCTAAGGCTGGGGAAAG
GAPDH mRNA	ACCTGACCTGCCGTCTAGAAAA	CAAAGTGGTCGTTGAGGGCAAT
QARS mRNA	ACCTGAACCTGGCATCACTACA	CCAAGACGCTCAAACTGGAACT
5S rRNA	GGCCATACCACCCTGAACGC	CAGCACCCGGTATTCCCAGG

3. Uncropped Ethynyl Uridine (EU) and Immunofluorescence images:

Figure 3A: Uncropped images

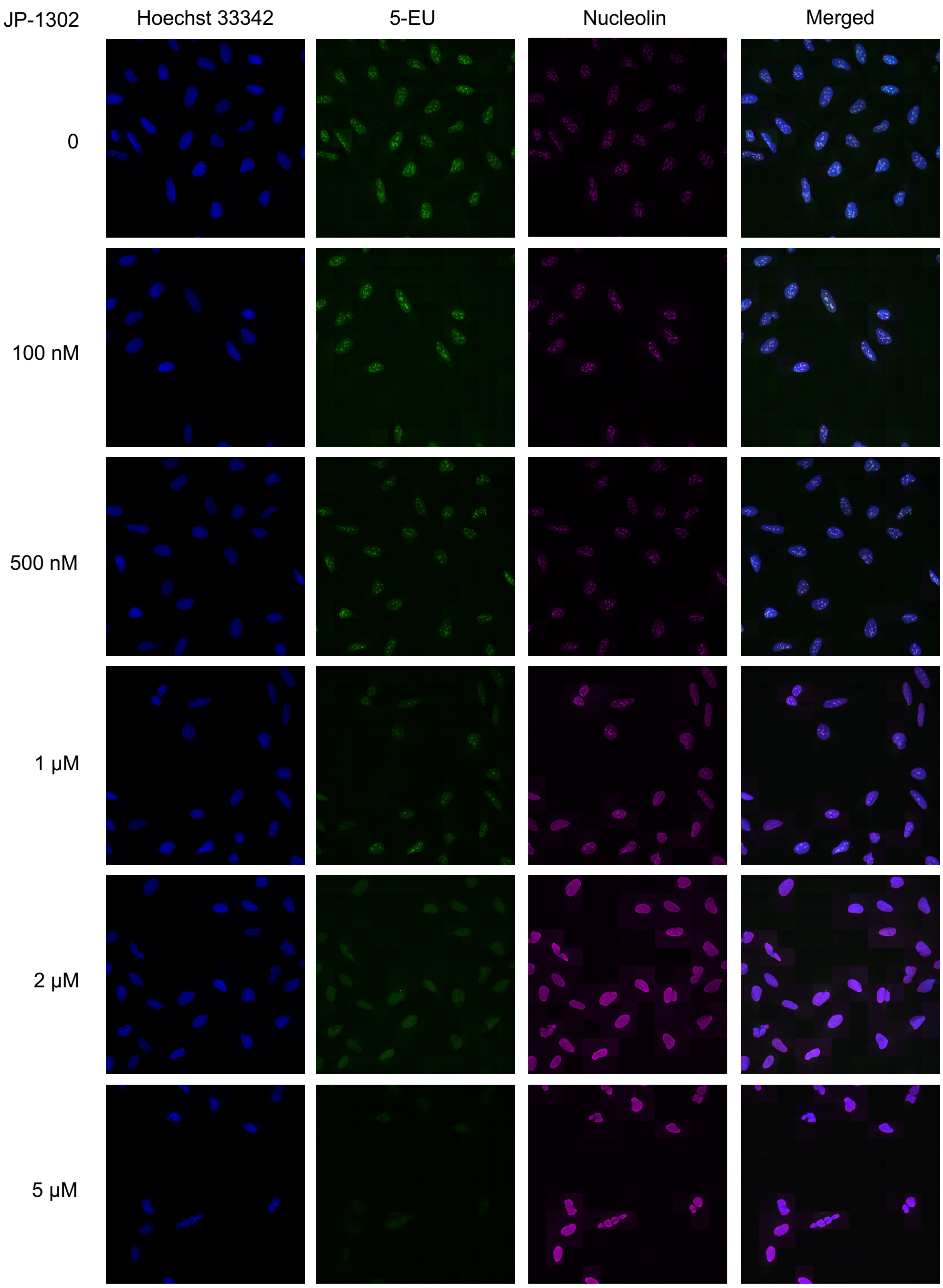

Figure 3B: Uncropped images

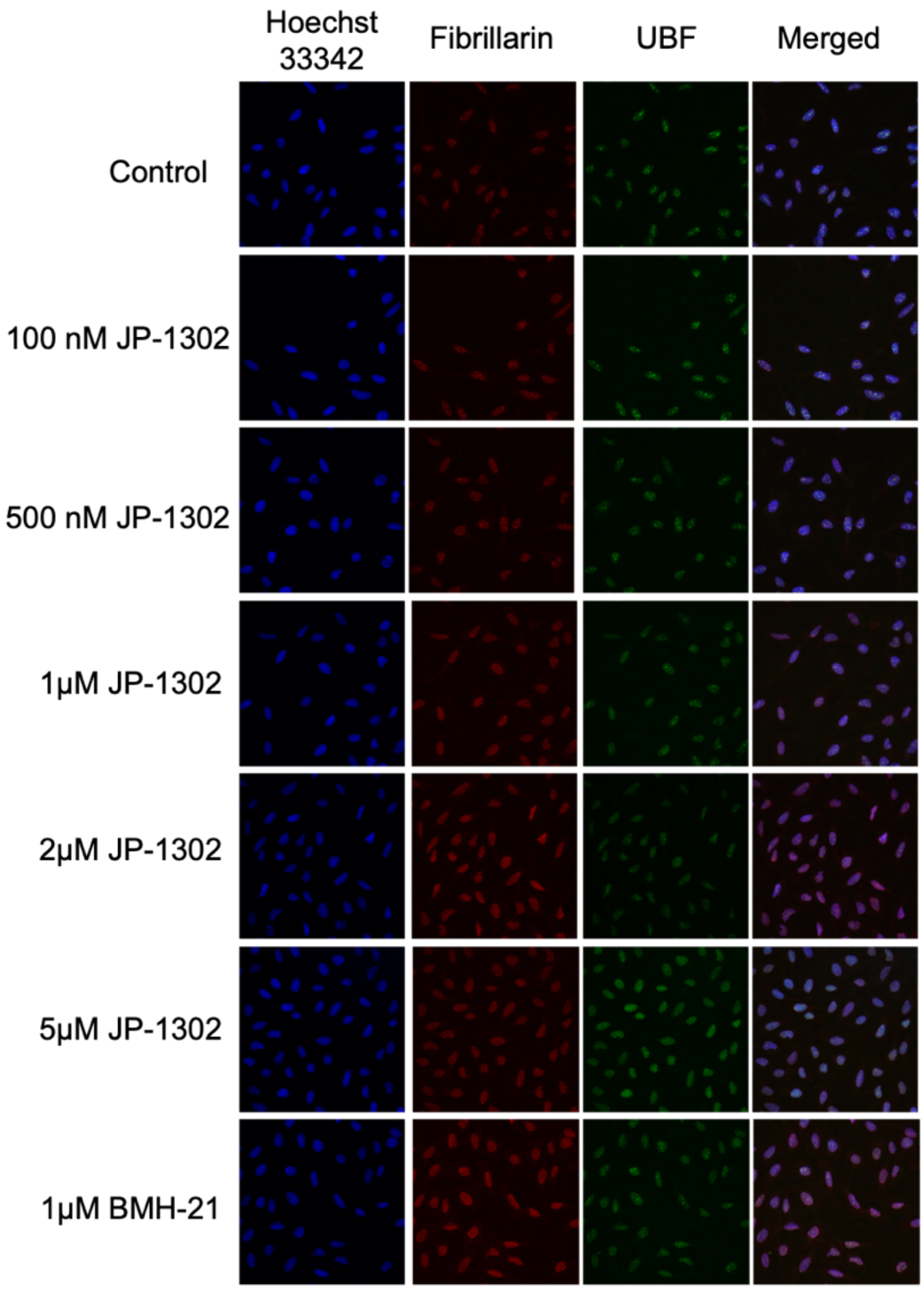

Figure 6B: Uncropped images

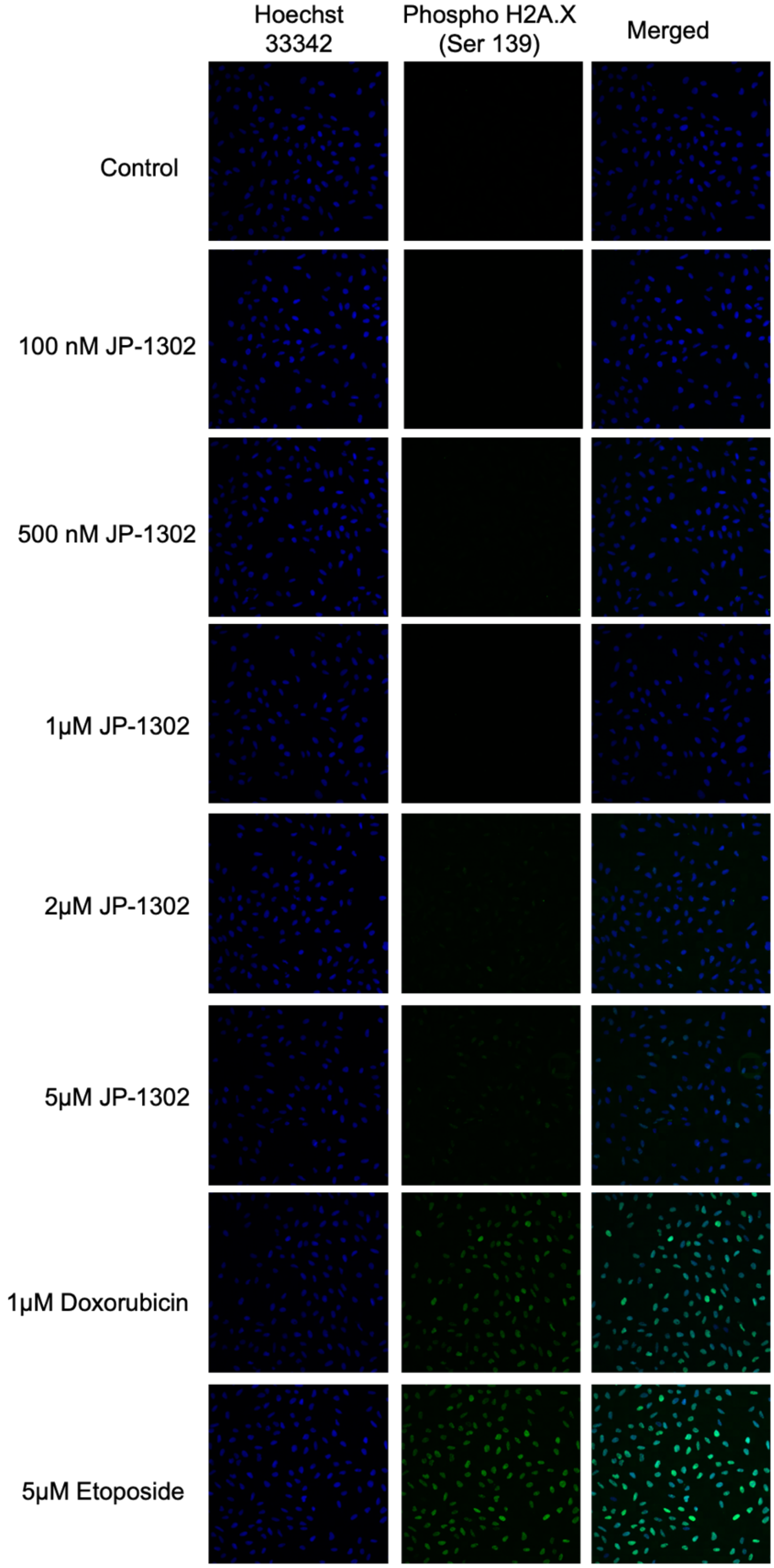

4. Supplemental figures:

Supplementary Figure S1: IC50 curves of cancer and non-cancerous cell lines treated with JP-1302

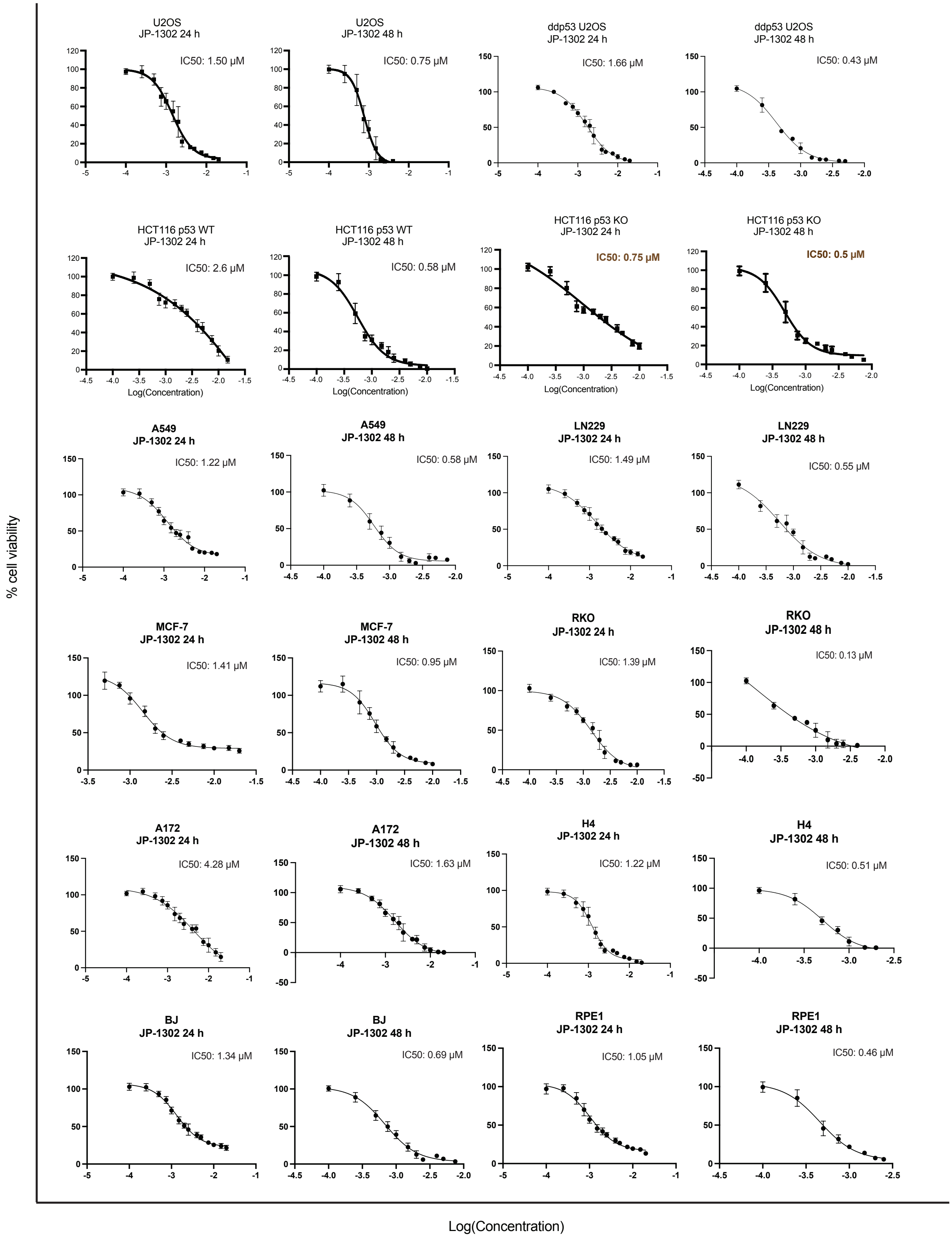

Supplementary Fig. S1. Dose response curves and IC50 values of JP-1302 in cancer and non-cancerous cell lines, as determined by Resazurin metabolic activity assay. The experiments for all the cell lines were performed in biological triplicates. The data is shown as mean ± SD.

Supplementary Figure S2: JP-1302 degraded POLR1A in cancer cell lines

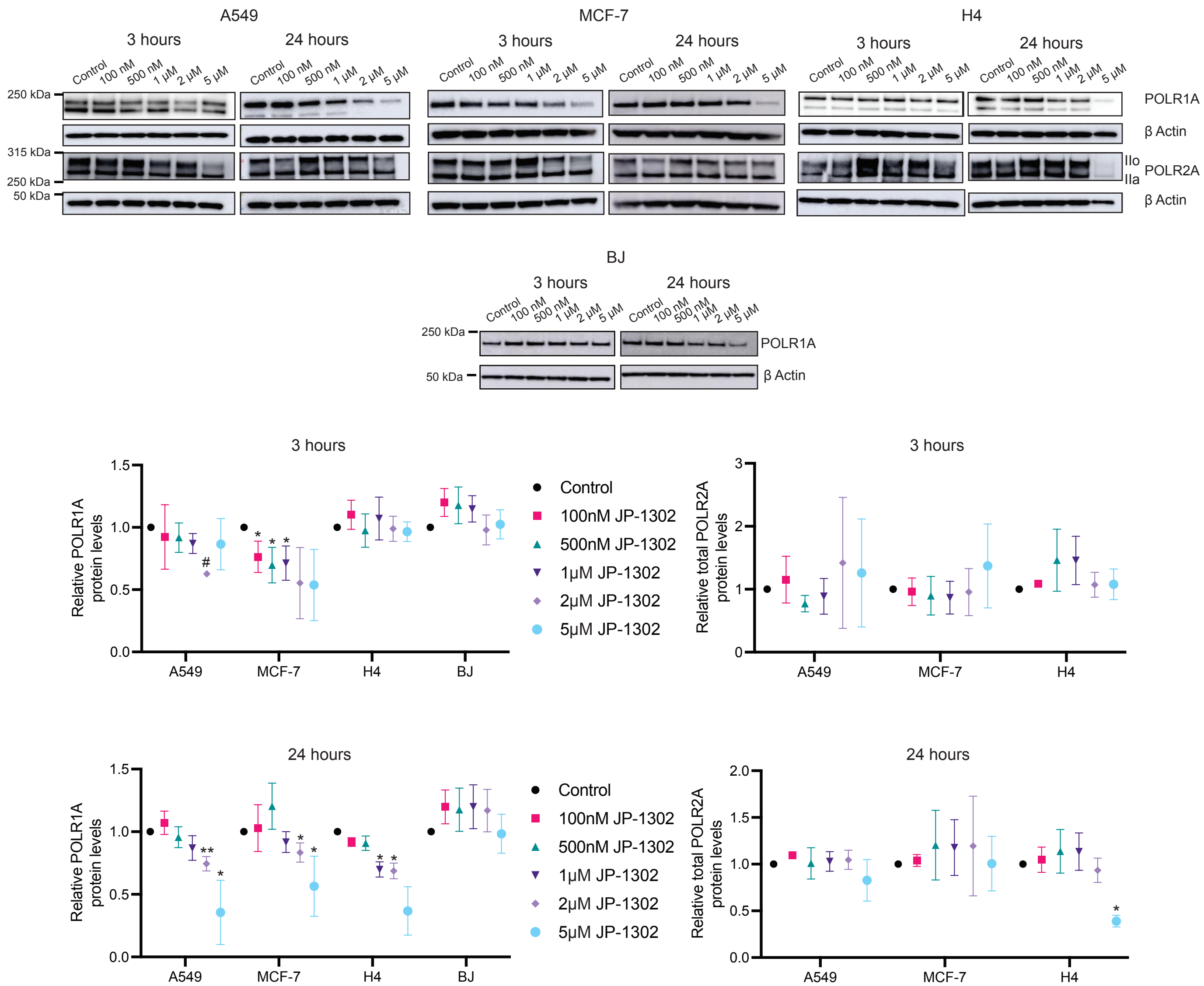

Supplementary Fig. S2. Immunoblots showing reduced POLR1A levels whereas total POLR2A levels were largely unaffected except at the highest concentration at 24 h in cancer cell lines. POLR1A levels were unaffected in non-cancerous, BJ cells. Lower panel shows immunoblot quantifications of biological triplicates presented as mean ± SD. The experiments for all the cell lines were performed in biological triplicates. Statistical significance was assessed using a two-tailed unpaired t-test. *$p < 0.05$, **$p < 0.01$, #$p < 1 \times 10^{-5}$.

Supplementary Figure S3: Relative POLR1A mRNA levels in response to JP-1302 treatment

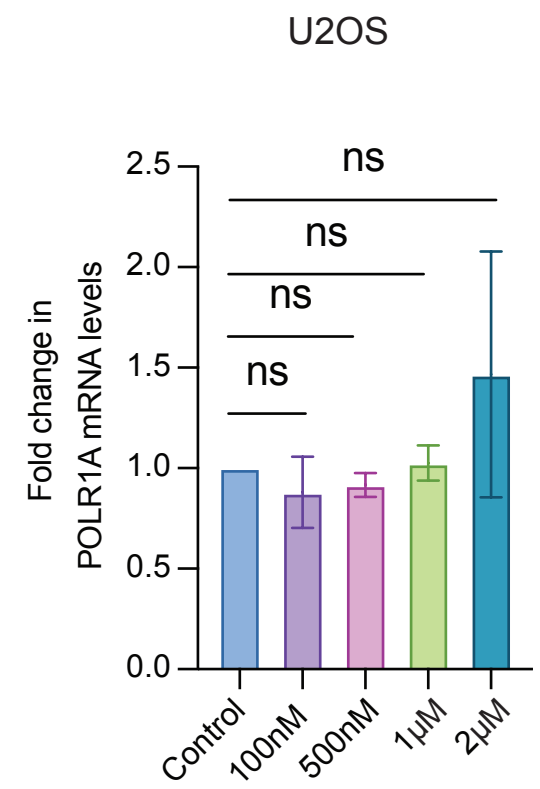

Supplementary Fig. S3. Bar plot showing fold change in *POLR1A* mRNA levels in response to varied concentrations of JP-1302. The data is shown as mean ± SD of biological triplicates. Statistical significance was calculated using two-tailed unpaired t-test. ns = not significant.

Supplementary Figure S4: Knockdown of alpha-2C adrenergic receptor (ADRA2C) did not affect POLR1A protein levels

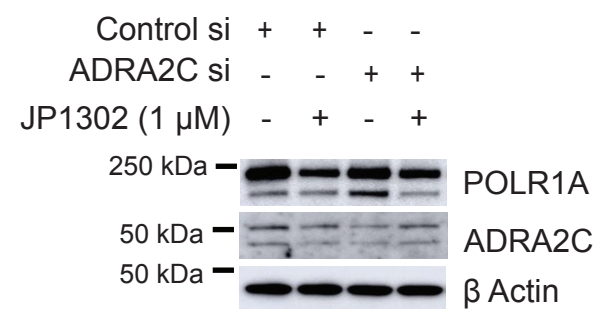

Supplementary Fig. S4. Immunoblot showing unchanged POLR1A protein levels in response to alpha-2C adrenergic receptor knockdown. The experiment was performed in biological triplicates.

Supplementary Figure S5: Effect of JP-1302 on p53 and p21 protein levels in cancer cell lines

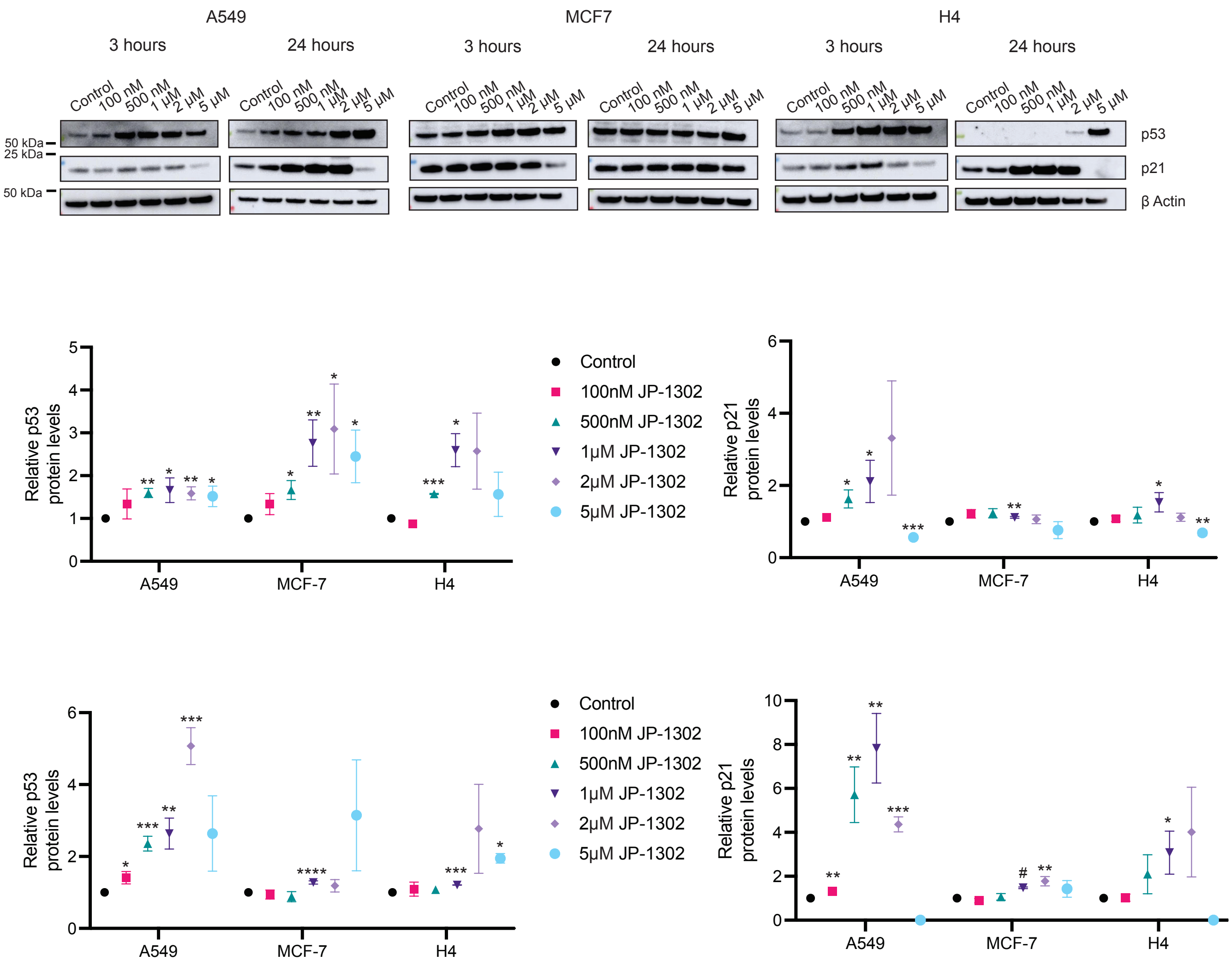

Supplementary Fig. S5. Immunoblots showing increased p53 and p21 levels in cancer cell lines in response to JP-1302 treatment. High concentration of JP-1302 (5 μM) reduced p21 levels. Lower panel shows immunoblot quantifications of biological triplicates presented as mean ± SD. The experiments for all the cell lines were performed in biological triplicates. Statistical significance was calculated using two-tailed unpaired t-test. **p < 0.05, **p < 0.01, ***p < 0.001, ****p < 0.0001, #p < 1 x 10⁻⁵ .*

Supplementary Figure S6: Structural model of JP-1302 intercalated into DNA hexamer duplex

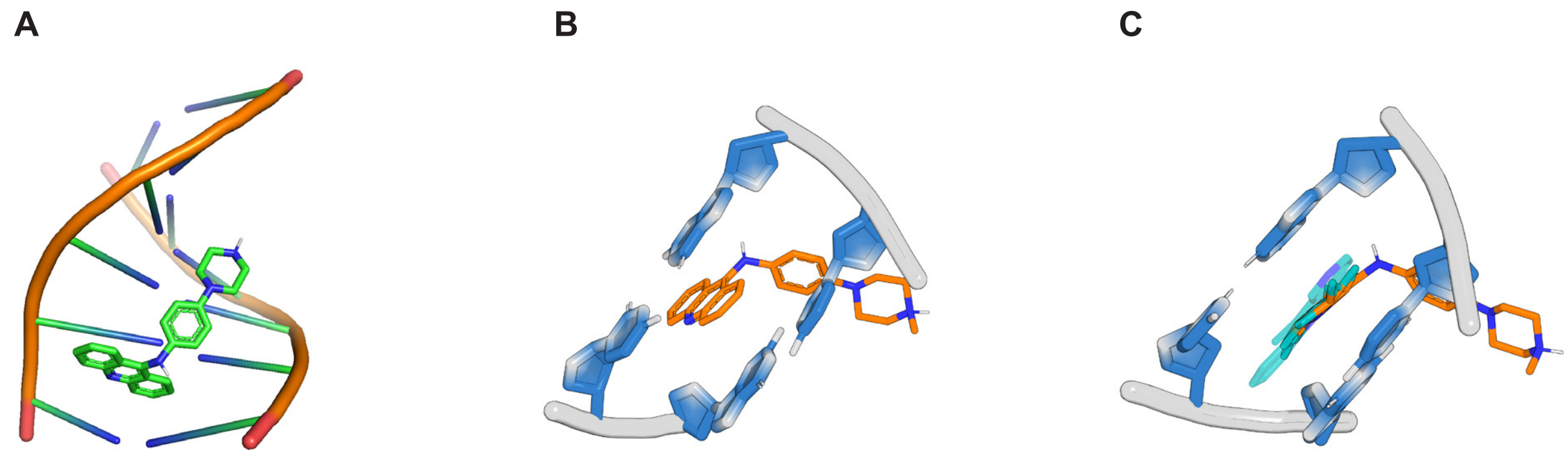

Supplementary Fig. S6. Structural model of JP-1302 intercalated into the d(CGATCG)$_2$ DNA hexamer duplex (PDB 1Z3F scaffold). **A., B.** Overview of the duplex with the top-ranked AutoDock Vina pose of JP-1302 (ΔG = -7.65 kcal/mol). **C.** Intercalation of JP-1302 (in orange) at the terminal CpG step, showing the acridine chromophore of JP-1302 sandwiched between the flanking base pairs in near-superposition with the experimental ellipticine reference (green, semi-transparent) at the same site.

Supplementary Figure S7: JP-1302 displaces EtBr across the tested DNA GC-content range

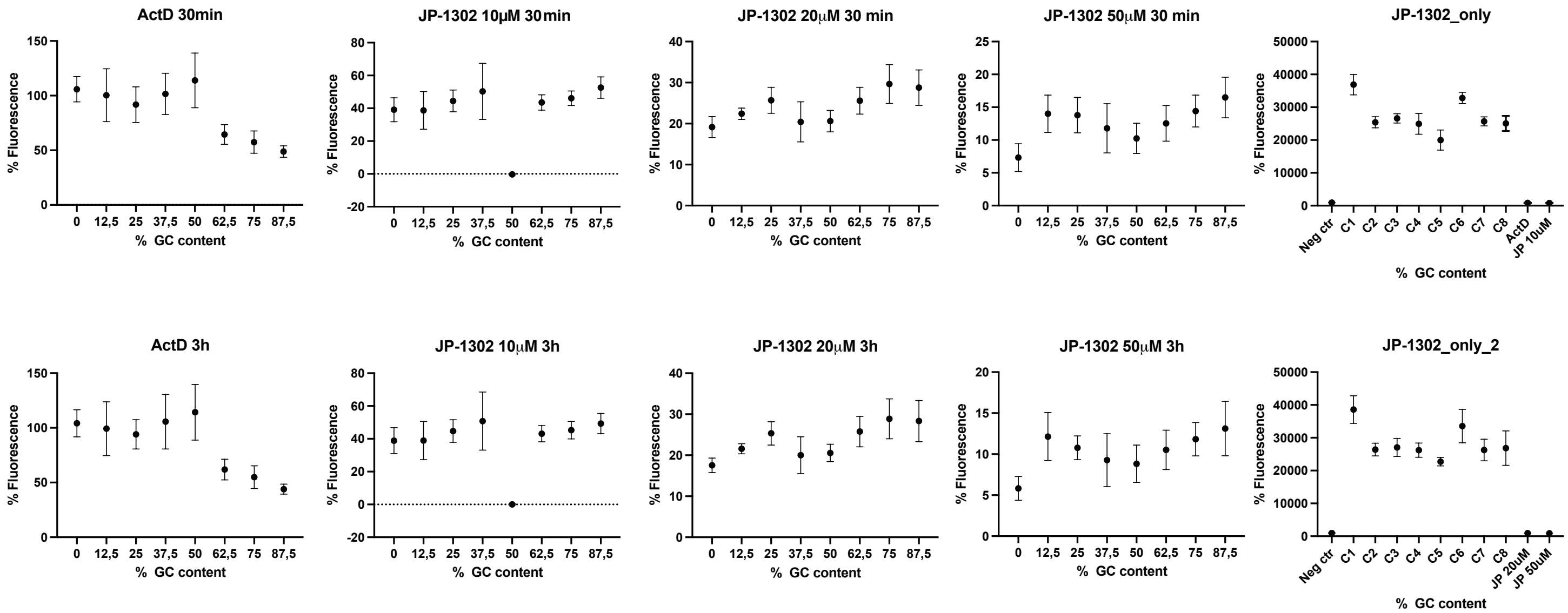

Supplementary Fig. S7. Fluorescence intercalator displacement (FID) assay showing displacement of ethidium bromide (EtBr) from oligonucleotide hairpins spanning a range of GC content (0-87.5%) by the reference GC-selective intercalator Actinomycin D (Act D, 2 µM) or by JP-1302. Data is presented as percentage of maximal EtBr-oligonucleotide fluorescence (100% control) after subtraction of background (0% control) fluorescence for each oligonucleotide. The experiment was performed in three replicates.

Supplementary Figure S8: JP-1302 depletes topoisomerase II and FACT from rDNA promoter

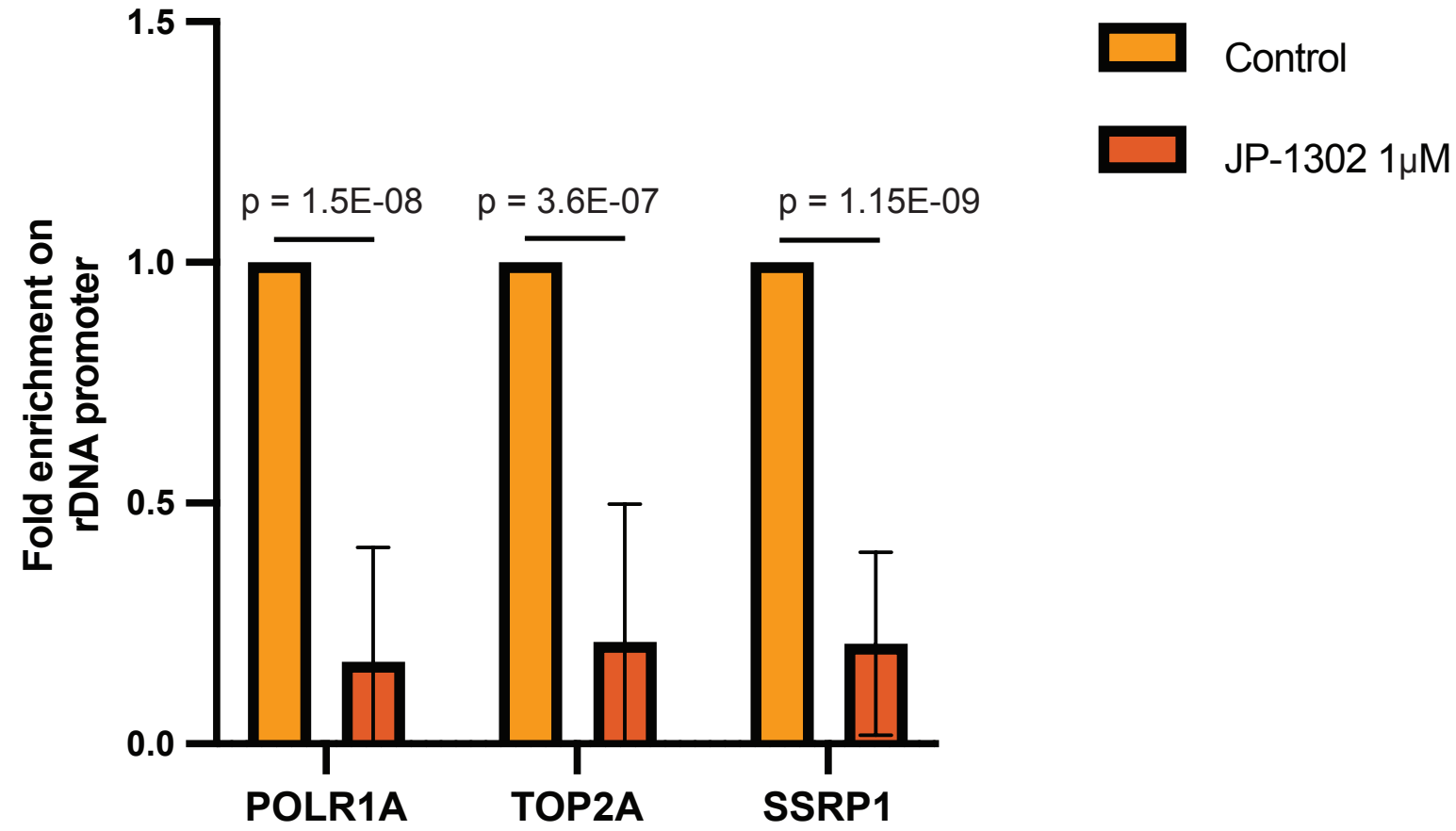

Supplementary Fig. S8. Chromatin immunoprecipitation (ChIP) assay showing reduced occupancy of POLR1A, topoisomerase II subunit TOP2A, and FACT component SSRP1 from ribosomal DNA (rDNA) promoter at region -250 to +102. The experiment was performed in three biological replicates. Bar graphs represent mean ± SD. Statistical significance was calculated using two-tailed unpaired t-test.
